# Electrooxidative *para*-selective C–H/N–H cross-coupling with hydrogen evolution to synthesize triarylamine derivatives

**DOI:** 10.1038/s41467-019-08414-8

**Published:** 2019-02-07

**Authors:** Kun Liu, Shan Tang, Ting Wu, Shengchun Wang, Minzhu Zou, Hengjiang Cong, Aiwen Lei

**Affiliations:** 1 College of Chemistry and Molecular Sciences, Institute for Advanced Studies (IAS), Wuhan University, Wuhan, 430072 P.R. China; 2 State Key Laboratory for Oxo Synthesis and Selective Oxidation, Lanzhou Institute of Chemical Physics, Chinese Academy of Sciences, Lanzhou, 730000 P.R. China

## Abstract

Oxidative C–H/N–H cross-coupling is one of the most atom-economical methods for the construction of C–N bonds. However, traditional oxidative C–H/N–H cross-coupling either required the use of strong oxidants or high reaction temperature, which makes it difficult to tolerate redox active functional groups. Herein we describe an external chemical oxidant-free electrooxidative C–H/N–H cross-coupling between electron-rich arenes and diarylamine derivatives. Under undivided electrolytic conditions, a series of triarylamine derivatives are produced from electron-rich arenes and diarylamine derivatives with high functional group tolerance. Both of the coupling partners are redox active in oxidative C–H/N–H cross-coupling, which enables high regioselectivity in C–N bond formation. Exclusive *para*-selectivity is observed for the coupling with anilines.

## Abstract (web summary)

Oxidative C–H/N–H cross-coupling is a convenient method to construct C–N bonds, however this is rarely achieved in a sustainable manner. Here, the authors show an oxidant-free electrochemical C–H/N–H cross-coupling between electron-rich arenes and diarylamines to afford triarylamines with exclusive *para*-selectivity.

## Introduction

Aryl C–N bond formation has long been considered to be important since C(sp^2^)–N bonds widely exist in pharmaceuticals, agrochemicals, and materials^1,2^. Normal ways to access C–N bonds are transition metal catalyzed amination of aryl halides or aryl boronic acids^3–6^. Recent achievements have revealed that oxidative C–H/N–H cross-coupling can serve as an atom-economical way to construct C–N bonds since it avoids substrate prefuncationalization steps^7–9^. However, research on oxidative aryl C–N bond formation remains elusive in its early stages when compared with the development of oxidative cross-coupling for C–C bond formation. The developed oxidative aryl C–N bond formation either required the use of strong oxidants^10–17^ or high reaction temperature^18–22^. As a result, redox active functional groups such as amino, hydroxyl, sulfur, and alkynyl are hard to be tolerated under these oxidative conditions.

**Fig. 1:**
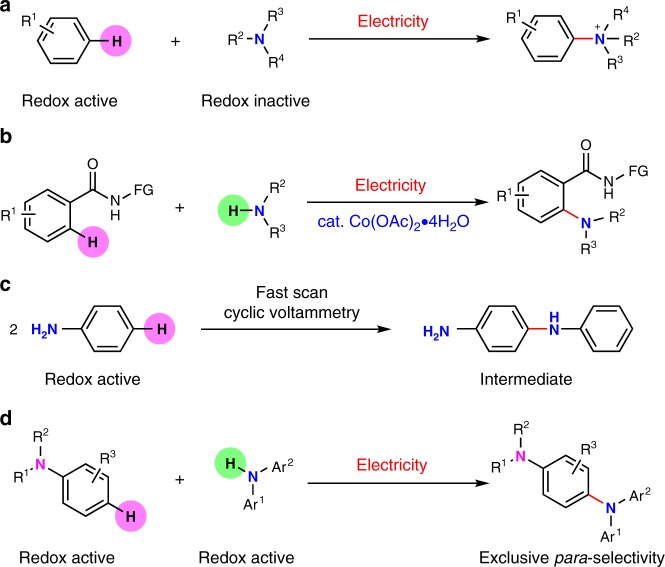
Electrooxidative intermolecular aryl C–H amination. **a** Electrooxidative aryl C–H amination of arenes with redox inactive pyridine and imidazoles. **b** Cobalt-catalyzed electrooxidative *ortho*-selective aryl C–H amination of arenes with secondary amines. **c** Electrooxidative homo-coupling of aniline in fast scan cyclic voltammetry. **d** Catalyst-free electrooxidative *para*-selective aryl C–H amination of anilines with diarylamine derivatives

An ideal way to solve this problem is to achieve oxidative C–H/N–H cross-coupling without the use of external chemical oxidants under mild conditions^23–26^. In 2016, our group developed an oxidative aryl C–H amination of arenes with azoles by utilizing synergistic cooperation of a photocatalyst and cobalt oxime complex^27^. No chemical oxidants were required and hydrogen gas was the only byproduct in that transformation. However, the amination source was limited to redox inactive azoles. The C–N bond was proposed to be formed through the nucleophilic addition of azoles to the in situ generated arene cation radical^28^. Electrochemistry provides new possibilities for green organic synthesis, which has attracted increasing interests in the area of cross-coupling reactions^29–42^. As early as 2013, Yoshida and co-workers reported analogous mechanism for aryl C–H amination of arenes by utilizing electrochemical oxidation (Fig. 1a)^43,44^. Similarly, the amination sources were limited to redox inactive pyridine and imidazoles. More recently, our group^45^ and Ackermann group^46^ independently studied the cobalt-catalyzed electrooxidative C–H amination of arenes with secondary amines (Fig. 1b). Due to the effect of directing group, only *ortho*-selective C–H amination products were obtained. In this transformation, none of the substrates were directly oxidized by anode. Up to now, there is no precedent on the electrooxidative aryl C–H amination with both redox active arenes and amination sources. Last year, our group have reported an electrooxidative C–H sulfenylation of electron-rich arenes with aromatic thiols^47^. Both of the coupling partners were redox active under the utilized electrolytic conditions. The high regioselectivity for C–S bond formation was believed to be a result of the radical/radical cross-coupling between the two coupling partners. As for electrooxidative C–H amination, we reported an oxidative C–H amination of phenols with phenothiazines recently^48^. Phenols were not oxidized due to relatively higher oxidation potentials. The amination source was limited to phenothiazines and both the *ortho* and *para*-amination products were observed. Aniline and its derivatives have been recognized as redox active substrates by electroanalytical techniques^49^. In 1992, Yang and Bard discovered that a *para*-selective homo-coupling product, *p*-amino diphenylamine, was formed as the dominate intermediate at the initial stage of electropolymerization of aniline in acidic aqueous solution (Fig. 1c)^50^. We envisioned that oxidative amination between two redox active coupling partners such as aniline derivatives might enable different regioselectivity and broaden the compatibility of functional groups in oxidative C–H amination.

Triarylamine derivatives have already demonstrated highly important applications as redox mediators^32^ and photocatalysts^51–54^. Meanwhile, triarylamine-based molecules have dramatically promoted the development of optoelectronic materials during the past decade^55–59^. Conventional approaches toward the installation of triarylamines largely rely on the cross-coupling of diarylamines with prefunctionalized aryl halides^60–63^. Here we report an electrooxidative C–H/N–H cross-coupling between electron-rich arenes and diarylamine derivatives under catalyst- and external chemical oxidant-free conditions. This method provides an atom-economical and easy-to-handle way to access a series of triarylamine derivatives with potential electronic and optical properties^64–66^. Interestingly, exclusive *para*-selective C–H amination of anilines is observed in this transformation (Fig. 1d). Since both of the substrates are redox active, C–N bonds are proposed to be formed through the radical/radical cross-coupling between in situ generated aniline radical cations and nitrogen radicals.

## Results

### Investigation of reaction conditions and substrate scope

**Fig. 2:**
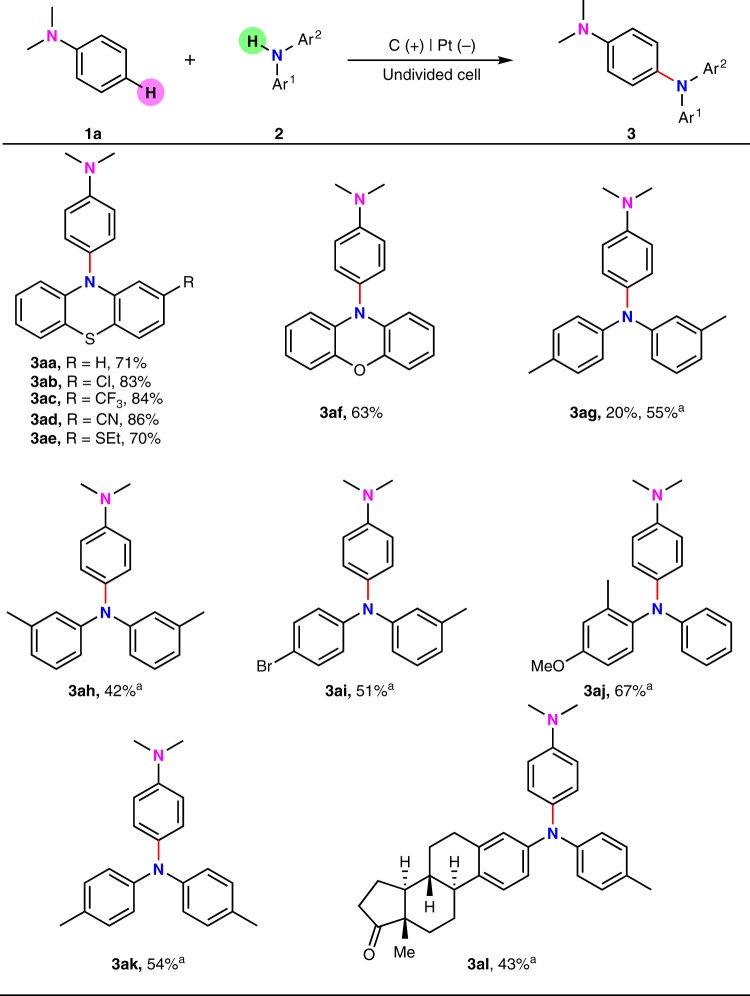
*para*-Selective C–H amination of *N,N*-dimethylaniline with different diarylamine derivatives. Reaction conditions: graphite rod anode (*ϕ* 6 mm), platinum plate cathode (15 mm × 15 mm × 0.3 mm), constant current = 7 mA (*J*_anode_ ≈ 7.8 mA cm^−2^), **1** (0.30 mmol), **2** (0.20 mmol), ^*n*^Bu_4_NBF_4_ (0.15 mmol), MeCN/MeOH (7.0 mL/3.0 mL), room temperature, N_2_, 2.0 h (2.6 F). Isolated yields were shown. ^a^Constant current = 12 mA (*J*_anode_ ≈ 13.3 mA cm^−2^), HFIP/MeCN (5.0 mL/5.0 mL)

Direct mixing *N,N*-dimethylaniline (**1a**) with phenothiazine (**2a**) under 7 mA constant current for 2 h in a simple undivided cell could furnish a selective *para*-selective oxidative C(sp^2^)–H/N–H cross-coupling product **3aa** in 71% yield (Fig. 2). Effect of reaction parameters was shown in Supplementary Information (Supplementary Table 1). The major side reaction is the dimerization and decomposition of **2a**^21^. At the same time, the homo-coupling product of **1a** could also be observed. In the next step, different amine substrates were applied to react with 1**a** (Fig. 2). Phenothiazines bearing electron-withdrawing and donating substituents were all able to furnish the desired products in good to high yields (**3ab-3ae**). Phenoxazine could also achieve good reaction efficiency in this oxidative C–H amination reaction (**3af**). Besides phenothiazine derivatives, diarylamines were also tested under the standard conditions. The reaction between **1a** and 3-methyl-*N*-(*p*-tolyl)aniline (**2g**) only afforded corresponding triphenylamine in 20% yield (**3ag**). The major side reaction is the decomposition of 3-methyl-N-(*p*-tolyl)aniline under the standard conditions. By using hexafluoro-2-propanol (HFIP) instead of methanol, the yield of **3ag** could be increased to 55% after certain degree of optimization. Interestingly, the *para*-selective homo-coupling product of **1a** could be isolated in 21% yield. Other diphenylamines with free *para* C–H bond were also able to couple with **1a** in acceptable yields (**3ah-3aj**). The reaction between **1a** and di-*p*-tolylamine afforded corresponding triphenylamine in 54% yield (**3ak**). Highly functionalized diarylamine with four continuous chiral centers could afford the desired triphenylamine in 43% yield (**3al**).

**Fig. 3:**
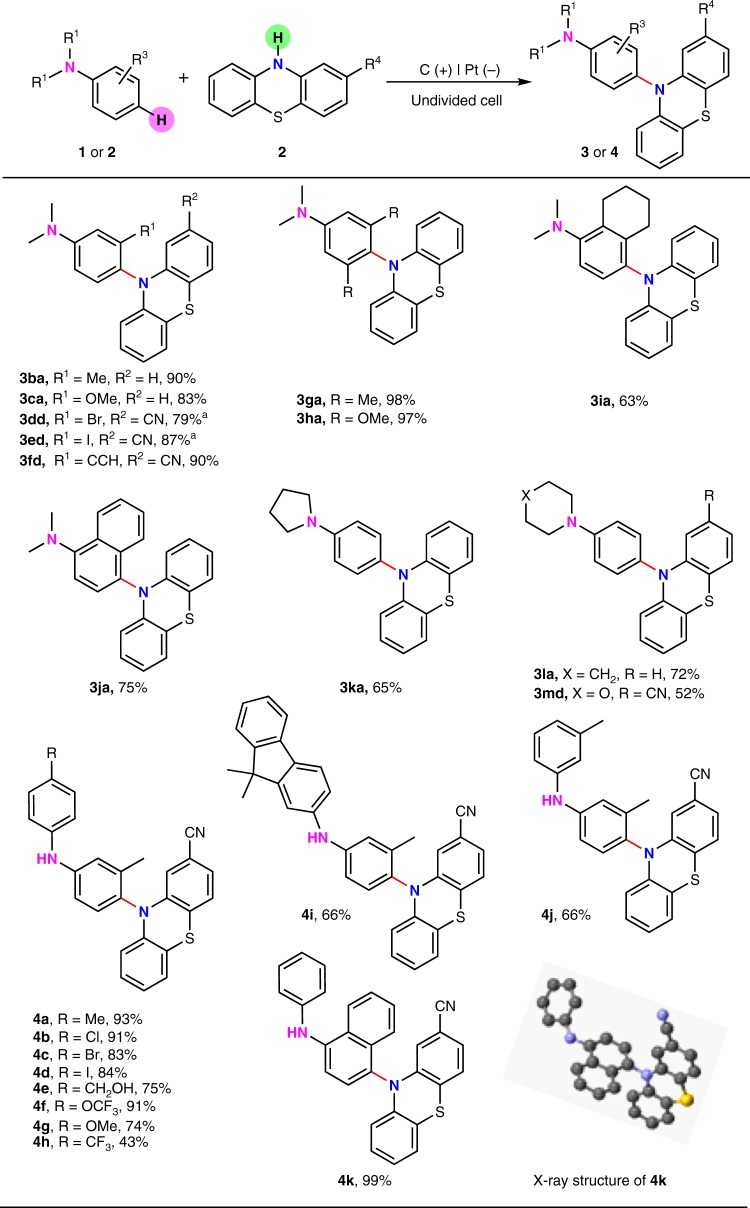
*para*-Selective C–H amination of different anilines. Reaction conditions: graphite rod anode (*ϕ* 6 mm), platinum plate cathode (15 mm × 15 mm × 0.3 mm), constant current = 7 mA (*J*_anode_ ≈ 7.8 mA cm^−2^), ^*n*^Bu_4_NBF_4_ (0.15 mmol), **1** (0.30 mmol), **2** (0.20 mmol), MeCN/MeOH (7.0 mL/3.0 mL), room temperature, N_2_, 2.0 h (2.6 F). Isolated yields were shown. ^a^Constant current = 10 mA (*J*_anode_ ≈ 11 mA cm^−2^)

In the next step, different anilines were applied as substrates in this transformation (Fig. 3). *N,N*-dimethylanilines bearing electron-donating methyl and methoxyl groups at the *meta*-position could afford the amination product in increased reaction yields (**3ba**, **3ca**). Halides including bromide and iodide were well tolerated in this process, which furnished the desired *para*-amination product with good reaction yields (**3dd**, **3ed**). Notably, 3-ethynyl-*N,N*-dimethylaniline was also suitable in this electrooxidative C–H amination reaction and afforded the selective *para*-amination product in 90% yield (**3fd**). *N,N*-dimethylanilines bearing two methyl or two methoxyl groups at the *meta*-position showed excellent reactivity in the amination reaction (**3ga**, **3ha**). As for the reaction with 2,3-disubstituted-*N,N*-dimethylaniline, *N,N*-dimethyl-5,6,7,8-tetrahydronaphthalen-1-amine could afford the *para*-amination product in 63% yield (**3ia**). Moreover, *N,N*-dimethylnaphthalen was compatible in this transformation as well, which afforded the desired amination product at C-4 position in high selectivity (**3ja**). Anilines with cyclic N-alkyl substituents also demonstrated good reactivity in this C(sp^2^)–N bond formation reaction (**3ka**, **3la**, and **3md**). It is worthy of noting that *N,N*-dimethylanilines bearing *para* substituents were unreactive in this transformation.

Diarylamine derivatives with free *para* C–H bonds were also able to react with phenothiazine-2-carbonitrile (Fig. 3). 3-Methyl-*N*-(*p*-tolyl)aniline showed a good reactivity in the synthesis of a *para* C–H amination product **4a**. As for the reaction with N-(4-halogenated phenyl)-3-methylanilines, the amination reaction showed an exclusive selectivity toward the *para* C–H position of the 3-methylaniline moiety and halides including chloride, bromide, and iodide were remained (**4b–4d**). Free hydroxyl group was also well tolerated (**4e**). Substrates bearing electron-donating substituents could still afford the desired product in good to high yields (**4f** and **4g**) while decreased reaction efficiency was observed for substrates bearing electron-withdrawing trifluoromethyl group (**4h**). 9,9-Dimethyl-N-(*m*-tolyl)-9H-fluoren-2-amine was also able to give a *para* amination product at the 3-methylaniline moiety (**4i**). A mono *para* C–H amination product could be observed for di-*m*-tolylamine in 66% yield under the standard conditions (**4j**). Notably, the reaction with N-phenylnaphthalen-2-amine demonstrated exclusive selectivity toward the N-naphthalen moiety with an excellent reaction efficiency (**4k**). Diphenylamines without free *para* C–H bonds such as di-*p*-tolylamine were unreactive in this electrolytic transformation.

**Fig. 4:**
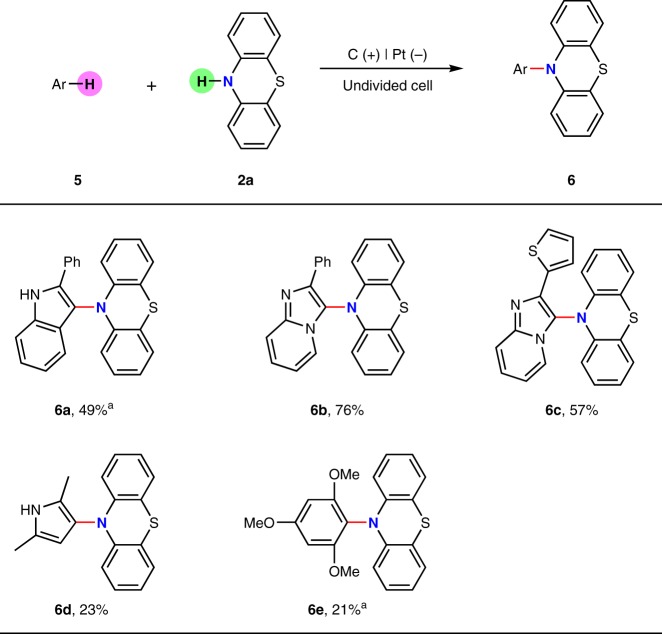
C–H amination of electron-rich arenes with phenothiazine. Reaction conditions: graphite rod anode (*ϕ* 6 mm), platinum plate cathode (15 mm × 15 mm × 0.3 mm), constant current = 12 mA (*J*_anode_ ≈ 13.3 mA cm^−2^), ^*n*^Bu_4_NBF_4_ (0.15 mmol), **5** (0.24 mmol), **2a** (0.20 mmol), MeCN/CF_3_CH_2_OH (7.0 mL/2.0 mL), room temperature, N_2_, 1.5 h (3.3 F). Isolated yields were shown. ^a^MeCN/AcOH (9.0 mL/1.0 mL)

Besides anilines, the reactivity of other electron-rich arenes were also tested in this electrooxidative C–H amination reactions with phenothiazine (Fig. 4). 2-Phenylindole could afford a C-3 amination product in 49% yield while 2-phenylimidazo[1,2-a]pyridine furnished the desired amination product in 76% yield (**6a**, **6b**). The reaction between 2-(thiophen-2-yl)imidazo[1,2-a]pyridine and phenothiazine demonstrated exclusive reactivity at the imidazo[1,2-a]pyridine ring with moderate yield (**6c**). 2,5-dimethylpyrrole and 1,3,5-trimethoxybenzene showed decreased reactivity in this transformation (**6d**, **6e**).

**Fig. 5:**
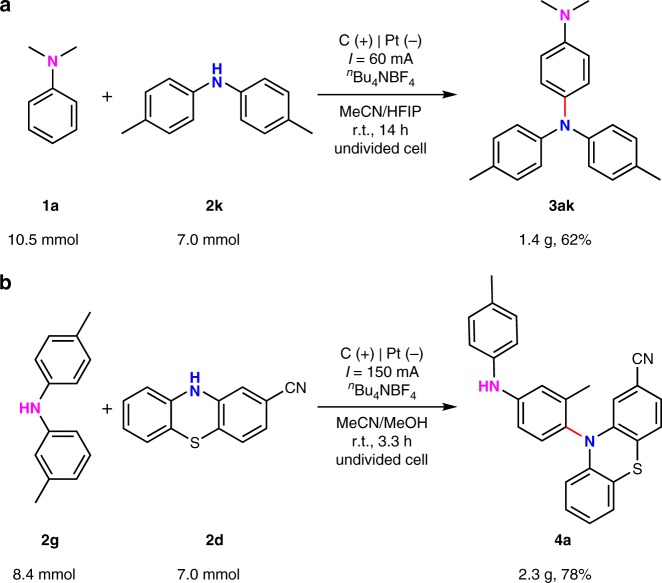
Large scale synthesis. **a** Gram-scale synthesis of **3ak**. **b** Gram-scale synthesis of **4a**

The scalability of this *para*-selective electrooxidative aryl C–H amination was then evaluated by performing 7.0 mmol scale reactions in a simple beaker equiped with graphite rod as the anode and platinum plate as the cathode. Under 60 mA constant current, the gram-scale reaction between **1a** and **2k** could give corresponding triphenylamine **3ak** in 62% yield (Fig. 5a). Under 150 mA constant current, the gram-scale reaction between **2g** and **2d** afforded **4a** in high selectivity with 78% yield (Fig. 5b).

## Discussion

**Fig. 6:**
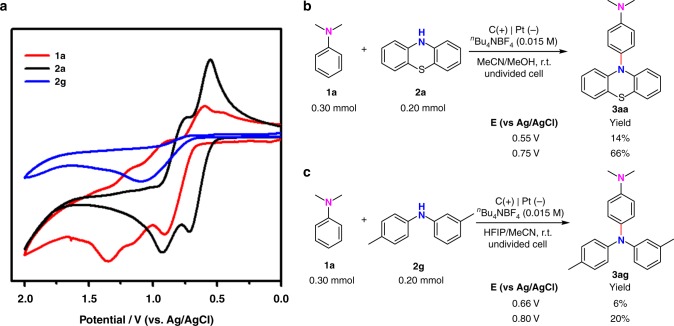
Study of the oxidation potential during electrolysis. **a** Cyclic voltammogram on a glassy carbon electrode (*ϕ* 3 mm) at 0.1 V s^−1^ under nitrogen. Red line, *N,N*-dimethylaniline (**1a**); black line, phenothiazine (**2a**); and blue line, 3-Methyl-N-(*p-*tolyl)aniline (**2g**). **b** Potential controlled electrolysis between **1a** and **2a**. **c** Potential controlled electrolysis between **1a** and **2g**

Since the method has been established, efforts were then paid to understand the mechanism for this selective oxidative C–H/N–H cross-coupling. First, cyclic voltammetry (CV) experiments were conducted to study the redox potential of the substrates. *N,N*-Dimethylaniline (**1a**) could be oxidized when the oxidation potential exceed 0.65 V (Fig. 6a, red line). Phenothiazine (**2a**) started to get oxidized from 0.50 V (Fig. 6a, black line) while 3-methyl-*N*-(*p-*tolyl)aniline (**2g**) could be oxidized from 0.70 V (Fig. 6a, blue line). Under the standard conditions in MeCN/MeOH, the voltage for whole electrolytic cell ranged from 3.10 to 3.42 V while the oxidation potential of anode (vs Ag/AgCl) ranged from 1.19 to 0.96 V. The two coupling partners could both be oxidized since the operating oxidation potential is higher than 0.96 V. This hypothesis could be supported by the fact that homo-coupling of both coupling partners were observed under the electrolytic conditions. Furthermore, experiments using potential controlled electrolysis were conducted to study the oxidation of the substrates. First, we tried to do the reaction between **1a** and **2a** at a controlled potential of 0.55 V where only **2a** could be oxidized. Only 14% yield of **3aa** could be obtained until complete consumption of the substrates. By contrast, 66% yield of **3aa** could be obtained when the reaction was conducted at a controlled potential of 0.75 V where both **1a** and **2a** could be oxidized (Fig. 6b). In the electrolytic reaction between **1a** and **2g**, only 6% yield of **3ag** could be obtained at a controlled potential of 0.66 V while 20% yield could be obtained at a controlled potential of 0.80 V (Fig. 6c). According to these results, we knew that best results were obtained when both of the substrates could be oxidized. Though with low yields, the reaction could also happen when only one of the substrates could be oxidized. Since the operating potentials under the standard reaction conditions were higher than the oxidation potential of both substrates, the cross-coupling of an arene radical cation and a nitrogen radical was likely to be the major reaction pathway.

**Fig. 7:**
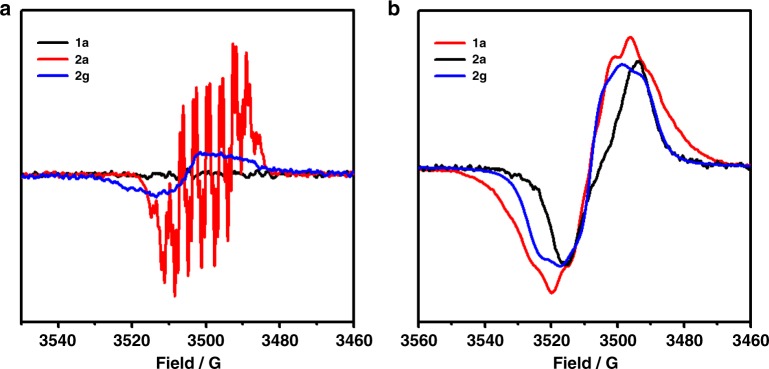
Electron paramagnetic resonance (EPR) spectra. **a** After electrolysis in MeCN/MeOH (7.0 mL/3.0 mL) for 15 min. **b** After electrolysis in MeCN/HFIP (5.0 mL/5.0 mL) for 15 min

To confirm the oxidation of the two coupling partners in Figs. 2-4, electron paramagnetic resonance (EPR) experiment (X band, 9.4 GHz, room temperature) was carried out to determine the oxidation species of **1a**, **2a,** and **2g** during the electrolysis. First, each substrate was separately electrolyzed under the standard conditions for 15 min. No obvious signals could be observed for **1a** (Fig. 7a, black line). Notably, a clear radical signal could be observed for **2a** (Fig. 7a, red line, *g* = 2.0058). The formation of a nitrogen radical was suggested (see Supplementary Fig. 1). However, only a very weak radical signal could be observed for **2g** (Fig. 7a, blue line, *g* = 2.0038). It has been reported that HFIP could stabilize radical cations^67,68^. When HFIP was used instead of MeOH in the EPR detection, clear radical signals could be observed for **1a** (Fig. 7b, red line, *g* = 2.0035), **2a** (Fig. 7b, black line, *g* = 2.0057), and **2g** (Fig. 7b, blue line, *g* = 2.0037). These results proved that dialkylamine, phenothiazine and diarylamine could all generate radical species through single-electron-transfer (SET) oxidation by the carbon anode under the electrolytic conditions.

**Fig. 8:**
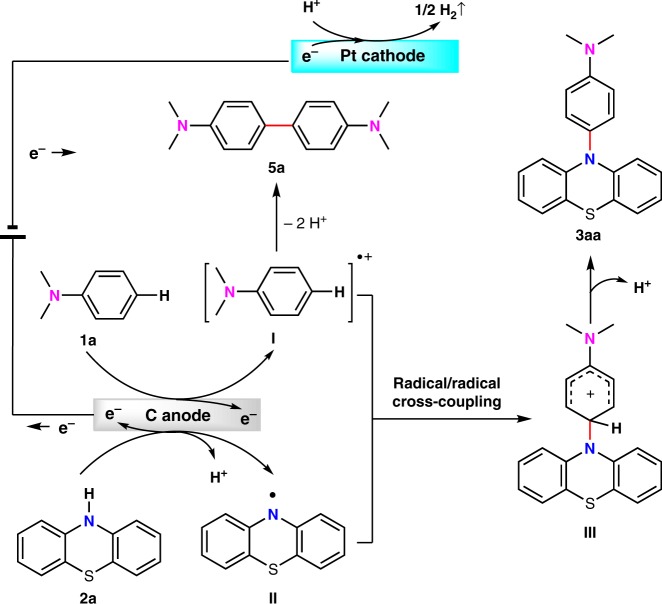
Proposed mechanism for the reaction between **1a** and **2a**. Tentative reaction mechanism involves anodic oxidation of aniline to generate aniline cation radical and diphenylamine to generate nitrogen radical, cross-coupling of aniline cation radical with nitrogen radical and deprotonation to furnish the final product

Based on the above experimental results, a plausible reaction mechanism between **1a** and **2a** was shown in Fig. 8. In the first step, **1a** could be oxidized at carbon anode to generate a radical cation **I**. Homo-coupling of radical cation **I** could lead to the formation of **5a**. At the same time, **2a** might also be oxidized by the carbon anode to generate a nitrogen radical **II**. C–N bond was likely to be formed from the radical/radical cross-coupling between radical cation **I** and nitrogen radical **II**. This unique reaction pathway might explain the exclusive *para*-selectivity for this oxidative C–H/N–H cross-coupling. Subsequent deprotonation of intermediate **III** could afford the final *para*-amination product. Concomitant cathodic reduction could release hydrogen gas during the reaction process. According to the results in Fig. 6, the cross-coupling of an arene radical cation and a nitrogen radical was likely to be the major reaction pathway. However, addition of nitrogen radical to aniline or nucleophilic addition of diarylamine derivatives to aniline radical cation were also possible to be involved as minor reaction pathways.

In summary, we have developed an electrooxidative C–H/N–H cross-coupling between electron-rich arenes and diarylamine derivatives under catalyst- and external oxidant-free conditions. Under undivided electrolytic conditions, exclusive *para*-selectivity are observed in the aryl C–H amination of anilines. This method provides a simple and efficient way to access triarylamine derivatives with high reaction efficiency. Highly active functional groups such as amino, hydroxyl, sulfur and even ethinyl could be well reserved after electrolysis. CV and EPR experiments suggest that C–N bonds are likely to be formed through the radical/radical cross-coupling between in situ generated aniline radical cations and nitrogen radicals.

## Methods

### General procedure for the electrooxidative C–H amination of *N,N*-dialkylanilines

In an oven-dried undivided three-necked bottle (25 mL) equipped with a stir bar, phenothiazine (0.20 mmol) and ^*n*^Bu_4_NBF_4_ (0.15 mmol) was added. The bottle was equipped with graphite rod (*ϕ* 6 mm, about 15 mm immersion depth in solution) as the anode and platinum plate (15 mm × 15 mm × 0.3 mm) as the cathode and charged with nitrogen. Subsequently, *N,N*-dialkylanilines (0.30 mmol) and CH_3_CN/MeOH (7.0 mL/3.0 mL) were added. Then the electrolysis system was stirred at a constant current of 7 mA under room temperature for 2 h. When the reaction was finished, the reaction mixture was washed with water and extracted with diethyl ether (10 mL × 3). The organic layers were combined, dried over Na_2_SO_4_, and concentrated. The pure product was obtained by flash column chromatography on silica gel (petroleum ether:ethyl acetate = 50:1). With diarylamines as the NH sources, diarylamines (0.20 mmol) and ^*n*^Bu_4_NBF_4_ (49.4 mg, 0.15 mmol) were added. The bottle was equipped with graphite rod (*ϕ* 6 mm, about 15 mm immersion depth in solution) as the anode and platinum plate (15 mm × 15 mm × 0.3 mm) as the cathode and then charged with nitrogen. Subsequently, *N,N*-dialkylanilines (0.30 mmol) and CH_3_CN/HFIP (5.0 mL/5.0 mL) were added. The reaction mixture was stirred and electrolyzed at a constant current of 12 mA under room temperature for 2 h. When the reaction was finished, the reaction mixture was washed with water and extracted with diethyl ether (10 mL × 3). The organic layers were combined, dried over Na_2_SO_4_, and concentrated. The pure product was obtained by flash column chromatography on silica gel (petroleum ether:ethyl acetate = 150:1). Full experimental details and characterization of the compounds are given in the Supplementary Information.

### General procedure for the electrooxidative C–H amination of diarylamines

In an oven-dried undivided three-necked bottle (25 mL) equipped with a stir bar, diarylamine (0.24 mmol), phenothiazine-2-carbonitrile (0.20 mmol), and ^*n*^Bu_4_NBF_4_ (0.15 mmol) were combined. The bottle was equipped with graphite rod (*ϕ* 6 mm, about 15 mm immersion depth in solution) as the anode and platinum plate (15 mm × 15 mm × 0.3 mm) as the cathode and was charged with nitrogen. Then CH_3_CN/MeOH (6.0 mL/4.0 mL) was added. The reaction mixture was stirred and electrolyzed at a constant current of 7 mA under room temperature for 2 h. When the reaction was finished, the reaction mixture was washed with water and extracted with diethyl ether (10 mL × 3). The organic layers were combined, dried over Na_2_SO_4_, and concentrated. The pure product was obtained by flash column chromatography on silica gel (petroleum ether:ethyl acetate = 50:1). Full experimental details and characterization of the compounds are given in the Supplementary Information.

## Supplementary information

Supplementary Information

## Data Availability

The X-ray crystallographic coordinates for structures reported in this article have been deposited at the Cambridge Crystallographic Data Centre (CCDC), under deposition number CCDC 1554125 (**4k**). The data can be obtained free of charge from The Cambridge Crystallographic Data Centre [http://www.ccdc.cam.ac.uk/data_request/cif]. The data supporting the findings of this study are available within the article and its Supplementary Information files. Any further relevant data are available from the authors on request.
