# Supplementary material for: Electrooxidative para-selective C–H/N–H cross-coupling with hydrogen evolution to synthesize triarylamine derivatives
Source: Nat Commun. 2019 Feb 7;10:639. doi: 10.1038/s41467-019-08414-8 (PMC6367370; doi:10.1038/s41467-019-08414-8)
Supplement: Supplementary file 1 — Supplementary Information [file 41467_2019_8414_MOESM1_ESM.pdf]

Electrooxidative *para*-selective C-H/N-H cross-coupling with hydrogen evolution to synthesize triarylamine derivatives

Liu et al.

Supplementary Methods

All glasswares were oven dried at 110 °C for hours and cooled down under vacuum. **1b-1j**[1], **1k-1m**[2], **2h-2k**[3], **2m** and **2n**[3], **2p-2s**[3], **2l**[4], **2o**[5] were prepared according to reported procedures. Unless otherwise noted, materials were obtained from commercial suppliers and used without further purification. The instrument for electrolysis is dual display potentiostat (DJS-292B) (made in China). Cyclic voltammograms were obtained on a CHI 605E potentiostat. The anodic electrode was graphite rod (ϕ 6 mm) and cathodic electrode was platinum plate (15 mm×15 mm×0.3 mm). Thin layer chromatography (TLC) employed glass 0.25 mm silica gel plates. Flash chromatography columns were packed with 200-300 mesh silica gel in petroleum ether (bp. 60-90 °C). EPR spectra were recorded on a Bruker X-band A-200 spectrometer. All new compounds were characterized by ^{1}H NMR, ^{13}C NMR and HRMS. The ^{1}H, ^{13}C and ^{19}F NMR spectra were recorded on a Bruker 400 MHz NMR spectrometer. For ^{1}H NMR, chemical shifts (δ) were given in ppm relatives to internal standard (TMS at 0 ppm, DMSO-d_6 at 2.50 ppm). For ^{13}C-NMR, chemical shifts (δ) were reported in ppm using solvent as internal standard ($CDCl_3$ at 77.00 ppm, DMSO-d_6 at 39.50 ppm). High resolution mass spectra (HRMS) were measured with a Waters Micromass GCT instrument and accurate masses were reported for the molecular ion (M+), molecular hydrogen ion $(M+H)^+$ or molecular anion $(M-H)^-$.

Procedure for gram scale synthesis of 3ak: In an undivided beaker (100 mL) equipped with a stir bar, *N,N*-dimethylaniline (10.5 mmol, 1.27 g), di-*p*-tolylamine (7.0 mmol, 1.38 g), $^{n}Bu_4NBF_4$ (0.494 g, 1.5 mmol) and CH_3CN/HFIP (50 mL/50 mL) were combined and added. The bottle was equipped with graphite rod (ϕ 6 mm, about 15 mm immersion depth in solution) as the anode and platinum plate (15 mm×15 mm×0.3 mm) as the cathode. The reaction mixture was stirred and electrolyzed at a constant current of 60 mA under air atmosphere at room temperature for 14 h (4.4 F). When the reaction was finished, the reaction mixture was washed with water and extracted with diethyl ether (100 mL x 3). The organic layers were combined, dried over Na_2SO_4, and concentrated. The pure product was obtained by flash column chromatography on silica gel (petroleum: ethyl ether = 150:1). White solid was obtained in 62% isolated yield (1.37 g).

Procedure for gram scale synthesis of 4a: In an oven-dried undivided beaker (100 mL) equipped with a stir bar, 3-methyl-*N*-(p-tolyl)aniline (8.4 mmol, 1.65 g), 10H-phenothiazine-2-carbonitrile (7.0 mmol, 1.57 g), $^{n}Bu_4NBF_4$ (0.658 g, 2.0 mmol) and CH_3CN/MeOH (60 mL/40 mL) were

combined and added. The bottle was equipped with graphite rod (ϕ 6 mm, about 15 mm immersion depth in solution) as the anode and platinum plate (15 mm×15 mm×0.3 mm) as the cathode. The reaction mixture was stirred and electrolyzed at a constant current of 150 mA under air atmosphere at room temperature for 3.3 h (2.6 F). When the reaction was finished, the reaction mixture was washed with water and extracted with diethyl ether (100 mL x 3). The organic layers were combined, dried over Na_2SO_4, and concentrated. The pure product was obtained by flash column chromatography on silica gel (petroleum: ethyl ether = 30:1). Yellow solid was obtained in 78% isolated yield (2.3 g).

Procedure for the potential controlled electrolysis of 1a with 2a or 2g: In an oven-dried undivided three-necked bottle (25 mL) equipped with a stir bar, *N,N*-dimethylaniline (0.30 mmol), phenothiazine (0.20 mmol), *n*Bu_4NBF_4 (49.4 mg, 0.15 mmol) and CH_3CN/MeOH (7.0 mL/3.0 mL) were combined and added. The bottle was equipped with graphite rod (ϕ 6 mm, about 15 mm immersion depth in solution) as the anode and platinum plate (15 mm×15 mm×0.3 mm) as the cathode and then charged with nitrogen. The reaction mixture was stirred and electrolyzed at a controlled potential of 0.55 and 0.75 V (vs Ag/AgCl) and stopped until complete consumption of substrate. With regard to the potential controlled electrolysis between **1a** and **2g**, *N,N*-dimethylaniline (0.30 mmol), 3-methyl-N-(p-tolyl)aniline (0.20 mmol), *n*Bu_4NBF_4 (49.4 mg, 0.15 mmol) and CH_3CN/HFIP (5.0 mL/5.0 mL) were combined and added. The bottle was equipped with graphite rod (ϕ 6 mm, about 15 mm immersion depth in solution) as the anode and platinum plate (15 mm×15 mm×0.3 mm) as the cathode and then charged with nitrogen. The reaction mixture was stirred and electrolyzed at a controlled potential of 0.66 and 0.80 V (vs Ag/AgCl) and stopped until complete consumption of substrate.

General procedure for cyclic voltammetry (CV): Cyclic voltammetry was performed in a three-electrode cell connected to a schlenk line under nitrogen at room temperature. The working electrode was a steady glassy carbon disk electrode, the counter electrode a platinum wire. The reference was a Ag/AgCl electrode submerged in saturated aqueous KCl solution, and separated from reaction by a salt bridge. 7.0 mL of acetonitrile and 3.0 mL of methanol containing 0.1 M $^{n}Bu_4NBF_4$ were poured into the electrochemical cell in all experiments. The scan rate is 0.1 V/s, ranging from 0 V to 2.0 V.

General procedure for the Electron Paramagnetic Resonance (EPR) experiment: 1a, **2a** and

2g were electrolyzed in MeCN/MeOH (7/3 mL) or MeCN/HFIP (5/5 mL) for 15 min, respectively. Then, the reaction solution was taken out by capillary and analyzed by EPR at room temperature. The samples were taken out by a capillary (borosilicate glass, 0.8-1.1×100 mm), and then recorded by EPR spectrometer at indicated temperature and parameters. The EPR measurement of a solution in MeCN/MeOH = 7/3 mL of $^{n}Bu_4NBF_4$, **2a** for 15 min under the constant current of 7 mA could obtain the following spectrum (Fig. S1, black line, g = 2.0058). After fitting, we proposed that this radical signal belongs to the two phenothiazine formed nitrogen radical (A_N= 7.1 g, A_H=3.7 g, A_H=3.7 g, A_H=3.7 g, A_H=3.6 g, A_H=0.86 g, A_H=0.86 g, A_H=0.86 g).

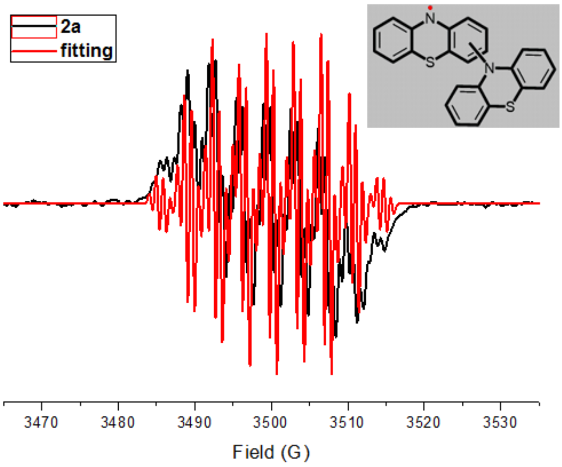

Supplementary Figure 1. EPR measurement of a solution in MeCN/MeOH = 7/3 mL of $^{n}Bu_4NBF_4$, **2a** under the constant current of 7 mA for 15 min.

Supplementary Table 1. Effect of Reaction Parameters*ᵃ*.

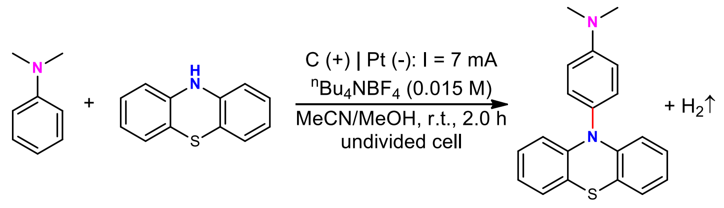

Entry	Variation from the standard conditions	Yield (%)
1	none	71
2	without MeOH	41
3	without MeCN	62
4	4 mA instead of 7 mA, 3.5 h	56
5	14 mA instead of 7 mA, 1 h	64

6	$^{n}Bu_4NClO_4$ instead of $^{n}Bu_4NBF_4$	68
7	$^{n}Bu_4NPF_6$ instead of $^{n}Bu_4NBF_4$	61
8	platinum plate anode	62
9	nickel plate cathode	68
10	graphite rod cathode	60
11	under air	63
12	without electric current, under air	n.r.

[a]Reaction conditions: graphite rod anode, platinum plate cathode, constant current = 7 mA, **1a** (1.5 equiv, 0.30 mmol), **2a** (1.0 equiv, 0.20 mmol), $^{n}Bu_4NBF_4$ (0.75 equiv, 0.15 mmol), MeCN/MeOH (7.0 mL/3.0 mL), room temperature, N_2, 2 h (2.6 F). Isolated yields were shown. n.r. = no reaction.

Crystallography Data of 4k: A single crystal of the compound was selected, mounted onto a cryoloop, and transferred in a cold nitrogen gas stream. Intensity data were collected with a BRUKER Kappa-APEXII diffractometer with graphite-monochromated Mo-Kα radiation (λ = 0.71073 Å). Data collection were performed with APEX2 suite (BRUKER). Unitcell parameters refinement, integration and data reduction were carried out with SAINT program (BRUKER). SADABS (BRUKER) was used for scaling and multi-scan absorption corrections. In the WinGX suite of programs, the structure were solved with Sir2014 program and refined by fullmatrix least-squares methods using SHELXL-14.

CCDC 1554125 contain the supplementary crystallographic data for this paper. These data can be obtained free of charge from The Cambridge Crystallographic Data Centre via www.ccdc.cam.ac.uk/data_request/cif.

Supplementary Table 2. Crystallography Data of **4k**.

Empirical formula	$C_{29}H_{19}N_3S$
Formula weight	441.1300
Space group	P 21/c
a (Å)	13.2989
b (Å)	8.2753
c (Å)	20.2677
α (deg)	90
β (deg)	100.654
γ (deg)	90
V ($Å^3$)	2192.1
Z	4

T (K)	296 K
ρ_{calcd} (g/cm^3)	1.338
μ (mm^{-1})	0.171
Significant reflections	5431
R[I > 2.5 *(I)*]	0.0469
R_w[I > 2.5 *(I)*]	0.1149

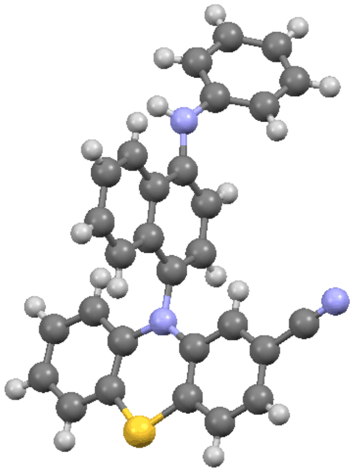

Supplementary Figure 2. Crystal structure of **4k**

Detail descriptions for products

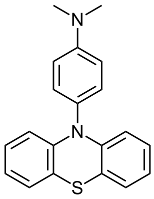

***N,N*-Dimethyl-4-(10H-phenothiazin-10-yl)aniline (3aa):** white solid was obtained in 72% isolated yield. ^{1}H NMR (400 MHz, DMSO-d_6) δ 7.24 – 7.16 (m, 2H), 7.03 (dd, J = 7.5, 1.6 Hz, 2H), 6.98 – 6.88 (m, 4H), 6.82 (td, J = 7.6, 1.2 Hz, 2H), 6.19 (dd, J = 8.0, 1.2 Hz, 2H), 3.01 (s, 6H). ^{13}C NMR (101 MHz, DMSO) δ 149.91, 144.41, 131.04, 127.91, 127.20, 126.44, 122.26, 118.48, 115.52, 113.81, 40.07. HRMS (ESI) calculated for $C_{20}H_{19}N_2S^+$ [M+H]$^+$: 319.1263; found: 319.1258.

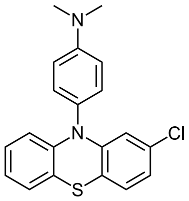

4-(2-Chloro-10H-phenothiazin-10-yl)-*N,N*-dimethylaniline (3ab): white solid was obtained in 83% isolated yield. ^{1}H NMR (400 MHz, $CDCl_3$) δ 7.21 – 7.14 (m, 2H), 6.95 (dd, *J* = 7.2, 1.6 Hz, 1H), 6.91 – 6.82 (t, *J* = 8.4 Hz, 3H), 6.79 (m, 2H), 6.72 (td, *J* = 8.0, 2.0 Hz, 1H), 6.23 (m, 2H), 3.05 (s, 6H). ^{13}C NMR (101 MHz, $CDCl_3$) δ 150.10, 146.15, 144.37, 132.56, 131.25, 128.24, 127.01, 126.94, 126.44, 122.42, 121.67, 119.07, 117.85, 115.96, 115.60, 113.77, 40.46. HRMS (ESI) calculated for $C_{20}H_{18}ClN_2S^+$ $[M+H]^+$: 353.0874; found: 353.0865.

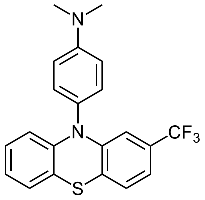

***N,N*-Dimethyl-4-(2-(trifluoromethyl)-10H-phenothiazin-10-yl)aniline (3ac)**: white solid was obtained in 84% isolated yield. ^{1}H NMR (400 MHz, DMSO-d_6) δ 7.26 – 7.19 (m, 3H), 7.21 (d, *J* = 8.0, 0.8 Hz, 1H), 7.03 (dd, *J* = 7.6, 1.6 Hz, 1H), 7.00 – 6.90 (m, 3H), 6.85 (td, *J* = 7.2, 1.2 Hz, 1H), 6.33 (d, *J* = 2.0 Hz, 1H), 6.15 (dd, *J* = 8.2, 1.0 Hz, 1H), 3.01 (s, 6H). ^{13}C NMR (101 MHz, DMSO-d_6) δ 150.06, 144.96, 143.49, 130.80, 127.69 (q, J_{C-F} = 31.3 Hz), 127.67, 127.17, 126.91, 126.54, 125.21, 124.22, 123.01, 118.60 (q, J_{C-F} = 4.4 Hz), 117.41, 115.94, 113.85, 111.71 (q, J_{C-F} = 4.4 Hz), 39.95. ^{19}F NMR (377 MHz, DMSO-d_6) δ -61.70. HRMS (ESI) calculated for $C_{21}H_{18}F_3N_2S^+$ $[M+H]^+$: 387.1137; found: 387.1134.

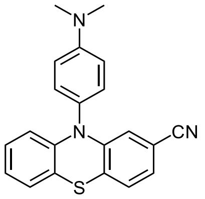

10-(4-(Dimethylamino)phenyl)-10H-phenothiazine-2-carbonitrile (3ad): white solid was obtained in 86% isolated yield. ^{1}H NMR (400 MHz, $CDCl_3$) δ 7.13 (d, *J* = 8.8 Hz, 2H), 6.95 (q, *J* = 7.9 Hz, 2H), 6.88 (t, *J* = 8.0 Hz, 3H), 6.82 -6.75 (m, 2H), 6.34 (s, 1H), 6.22 (d, *J* = 8.0 Hz, 1H), 3.06 (s, 6H). ^{13}C NMR (101 MHz, $CDCl_3$) δ 150.25, 145.42, 143.63, 130.90, 127.39, 127.37, 126.57, 126.49, 126.35, 125.22, 122.76, 119.05, 117.71, 117.56, 116.05, 113.94, 109.94, 40.37. HRMS (ESI) calculated for $C_{21}H_{18}N_3S^+$ $[M+H]^+$: 344.1216; found: 344.1213.

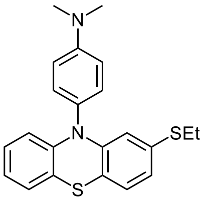

4-(2-(Ethylthio)-10H-phenothiazin-10-yl)-*N,N*-dimethylaniline (3ae): white solid was obtained in 70% isolated yield. ^{1}H NMR (400 MHz, DMSO-d_6) δ 7.25 – 7.17 (m, 2H), 7.05 – 7.01 (m, 1H), 7.05 – 6.87 (m, 4H), 6.88 – 6.76 (m, 2H), 6.17 (d, *J* = 8.0 Hz, 1H), 6.09 (d, *J* = 1.8 Hz, 1H), 3.02 (s, 6H), 2.76 (q, *J* = 7.3 Hz, 2H), 1.11 (t, *J* = 7.3 Hz, 3H). ^{13}C NMR (101 MHz, $CDCl_3$) δ 149.98, 144.78, 144.06, 134.92, 130.95, 127.56, 127.25, 126.86, 126.46, 122.45, 121.67, 118.44, 115.91, 115.73, 115.22, 113.78, 40.06, 26.43, 14.17. HRMS (ESI) calculated for $C_{22}H_{23}N_2S_2^+$ $[M+H]^+$: 379.1297; found: 379.1290.

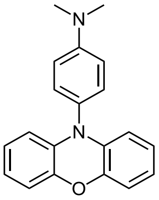

***N,N*-Dimethyl-4-(10H-phenoxazin-10-yl)aniline (3af):** white solid was obtained in 63% isolated yield. ^{1}H NMR (400 MHz, $CDCl_3$) δ 7.14 (dt, *J* = 10.0, 2.8 Hz, 2H), 6.85 (dt, *J* = 10.0, 2.8 Hz, 2H), 6.69 – 6.52 (m, 6H), 6.01 – 5.93 (m, 2H), 3.02 (s, 6H). ^{13}C NMR (101 MHz, $CDCl_3$) δ 150.05, 143.97, 135.13, 131.05, 126.93, 123.15, 120.73, 115.09, 113.96, 113.21, 40.51. HRMS (ESI) calculated for $C_{20}H_{19}N_2O^+$ $[M+H]^+$: 303.1492; found: 303.1492.

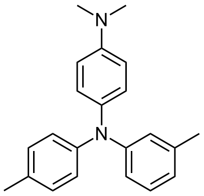

N^1,N^1-Dimethyl-N^4-(m-tolyl)-N^4-(p-tolyl)benzene-1,4-diamine (3ag): pale yellow oil was obtained in 55% isolated yield. 1H NMR (400 MHz, DMSO-d6) δ 7.08 – 7.01 (m, 3H), 6.94 – 6.87 (m, 2H), 6.86 – 6.79 (m, 2H), 6.74 – 6.60 (m, 5H), 2.87 (s, 6H), 2.22 (s, 3H), 2.15 (s, 3H). ^{13}C NMR (101 MHz, DMSO) δ 148.22, 147.52, 145.38, 138.21, 136.29, 130.84, 129.75, 128.91, 127.21, 122.90, 121.51, 121.31, 118.10, 113.55, 40.38, 21.19, 20.35. HRMS (ESI) calculated for $C_{22}H_{25}N_2^+$ $[M+H]^+$: 317.2012; found: 317.2010.

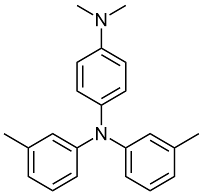

N^1,N^1-dimethyl-N^4,N^4-di-m-tolylbenzene-1,4-diamine (3ah): pale yellow oil was obtained in 42% isolated yield. ^{1}H NMR (400 MHz, DMSO-d^6) δ 7.13 – 7.05 (m, 2H), 6.94 – 6.88 (m, 2H), 6.75 – 6.65 (m, 8H), 2.88 (s, 6H), 2.17 (s, 6H). ^{13}C NMR (101 MHz, DMSO-d^6) δ 147.95, 147.59, 138.29, 136.05, 128.93, 127.46, 122.37, 122.12, 119.10, 113.48, 40.29, 21.11. HRMS (ESI) calculated for $C_{22}H_{25}N_2^+$ [M+H]$^+$: 317.2012; found: 317.2008.

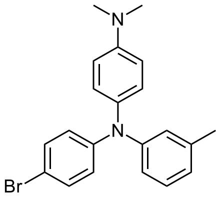

***N*^{1}-(4-Bromophenyl)-*N*^{4},*N*^{4}-dimethyl-*N*^{1}-(m-tolyl)benzene-1,4-diamine (3ai):** pale yellow oil was obtained in 51% isolated yield. ^{1}H NMR (400 MHz, DMSO-d^6) δ 7.37 – 7.28 (m, 2H), 7.20 – 7.07 (m, 1H), 6.97 – 6.90 (m, 2H), 6.85 – 6.62 (m, 7H), 2.88 (s, 6H), 2.19 (s, 3H). ^{13}C NMR (101 MHz, DMSO-d^6) δ 148.44, 147.93, 147.59, 139.14, 135.75, 132.22, 129.68, 128.13, 123.88, 123.77, 122.78, 120.61, 114.03, 112.19, 40.73, 21.53. HRMS (ESI) calculated for $C_{21}H_{22}BrN_2^+$ [M+H]$^+$: 381.0961; found: 381.0958.

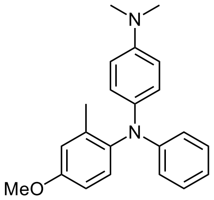

***N*^{1}-(4-Methoxy-2-methylphenyl)-*N*^{4},*N*^{4}-dimethyl-*N*^{1}-phenylbenzene-1,4-diamine (3aj):** white solid was obtained in 67% isolated yield. ^{1}H NMR (400 MHz, $CDCl_3$) δ 7.16 – 7.09 (m, 2H), 7.06 (d, *J* = 8.4 Hz, 1H), 7.03 – 6.96 (m, 2H), 6.79 – 6.71 (m, 5H), 6.69 – 6.63 (m, 2H), 3.79 (s, 3H), 2.90 (s, 6H), 2.07 (s, 3H). ^{13}C NMR (101 MHz, $CDCl_3$) δ 157.24, 148.76, 146.72, 138.60, 137.84, 137.21, 130.44, 128.75, 125.04, 118.75, 117.87, 116.39, 113.61, 112.45, 55.32, 41.06, 18.80. HRMS (ESI) calculated for $C_{22}H_{25}N_2O^+$ [M+H]$^+$: 333.1961; found: 333.1960.

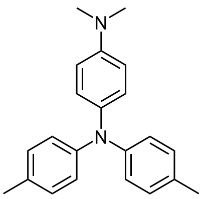

***N*^1^,*N*^1^-Dimethyl-*N*^4^,*N*^4^-di-p-tolylbenzene-1,4-diamine (3ak):** white solid was obtained in 54% isolated yield. ^{1}H NMR (400 MHz, DMSO-d^{6}) δ 7.00 (d, *J* = 8.0 Hz, 4H), 6.92 – 6.84 (m, 2H), 6.82 – 6.74 (m, 4H), 6.69 (d, *J* = 8.4 Hz, 2H), 2.86 (s, 6H), 2.21 (s, 6H). ^{13}C NMR (101 MHz, DMSO) δ 145.66, 145.61, 136.59, 130.10, 129.64, 126.78, 121.93, 113.58, 40.42, 20.28. HRMS (ESI) calculated for $C_{22}H_{25}N_2^+$ [M+H]$^+$: 317.2012; found: 317.2010.

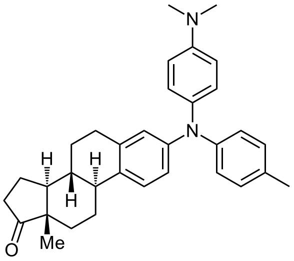

(8R,9S,13S,14S)-3-((4-(dimethylamino)phenyl)(p-tolyl)amino)-13-methyl-7,8,9,11,12,13,15,16-octahydro-6H-cyclopenta[a]phenanthren-17(14H)-one (3al): white solid was obtained in 43% isolated yield. ^{1}H NMR (400 MHz, $CDCl_3$) δ 7.15 – 6.90 (m, 7H), 6.84 – 6.63 (m, 4H), 2.93 (s, 6H), 2.82 – 2.69 (m, 2H), 2.50 (dd, *J* = 18.9, 8.5 Hz, 1H), 2.37 (dt, *J* = 12.4, 3.8 Hz, 1H), 2.31 – 2.23 (m, 3H), 2.19 – 2.00 (m, 2H), 1.99 – 1.89 (m, 2H), 1.70 – 1.35 (m, 6H), 1.34 – 1.23 (m, 1H), 0.91 (s, 3H). ^{13}C NMR (101 MHz, $CDCl_3$) δ 221.07, 147.32, 146.29, 145.86, 137.48, 136.96, 132.27, 130.92, 129.54, 127.26, 125.72, 123.04, 121.73, 119.46, 113.55, 50.45, 48.03, 44.16, 40.91, 38.31, 35.88, 31.58, 29.51, 26.61, 25.75, 21.57, 20.70, 13.88. HRMS (ESI) calculated for $C_{33}H_{39}N_2O^+$ [M+H]$^+$: 479.3057; found: 479.3055.

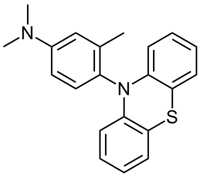

***N*,*N*,3-Trimethyl-4-(10H-phenothiazin-10-yl)aniline (3ba):** white solid was obtained in 90% isolated yield. ^{1}H NMR (400 MHz, $CDCl_3$) δ 7.16 – 7.08 (m, 1H), 6.91 (dd, *J* = 7.6, 1.6 Hz, 2H), 6.78 (td, *J* = 7.2, 1.6 Hz, 2H), 6.75 – 6.66 (m, 4H), 6.08 (dd, *J* = 8.0, 1.2 Hz, 2H), 3.01 (s, 6H), 2.15 (s, 3H). ^{13}C NMR (101 MHz, $CDCl_3$) δ 150.15, 143.55, 138.66, 131.68, 127.74, 126.90, 126.27,

121.80, 118.80, 114.96, 114.88, 111.62, 40.51, 18.19. HRMS (ESI) calculated for $C_{21}H_{21}N_2S^+$ $[M+H]^+$: 333.1420; found: 333.1418.

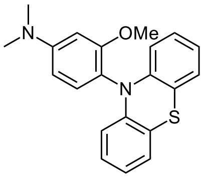

3-Methoxy-*N,N*-dimethyl-4-(10H-phenothiazin-10-yl)aniline (3ca): white solid was obtained in 83% isolated yield. ^{1}H NMR (400 MHz, $CDCl_3$) δ 7.13 (dt, *J* = 9.2, 1.3 Hz, 1H), 6.93 (dd, *J* = 7.2, 1.6 Hz, 2H), 6.87 – 6.78 (m, 2H), 6.73 (td, *J* = 7.6, 1.2 Hz, 2H), 6.40 (m, 2H), 6.14 (dd, *J* = 8.4, 1.6 Hz, 2H), 3.76 (s, 3H), 3.04 (s, 6H). ^{13}C NMR (101 MHz, $CDCl_3$) δ 158.14, 151.77, 144.18, 131.96, 126.80, 126.21, 121.73, 119.37, 117.64, 115.39, 105.41, 96.51, 55.36, 40.57. HRMS (ESI) calculated for $C_{21}H_{21}N_2OS^+$ $[M+H]^+$: 349.1369; found: 349.1376.

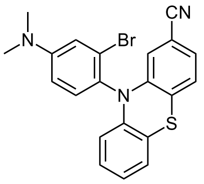

10-(2-Bromo-4-(dimethylamino)phenyl)-10H-phenothiazine-2-carbonitrile (3dd): yellow solid was obtained in 79% isolated yield. ^{1}H NMR (400 MHz, DMSO-d_6) δ 7.35 (d, *J* = 8.8 Hz, 1H), 7.21 (dd, *J* = 7.6, 1.6 Hz, 1H), 7.18 – 7.12 (m, 2H), 6.98 (dd, *J* = 7.2, 1.6 Hz, 1H), 6.95 – 6.89 (m, 2H), 6.83 (td, *J* = 7.2, 1.2 Hz, 1H), 6.09 (d, *J* = 1.6 Hz, 1H), 5.98 (d, *J* = 8.0 Hz, 1H), 3.01 (s, 6H). ^{13}C NMR (101 MHz, DMSO-d_6) δ 151.19, 142.90, 141.17, 132.54, 131.87, 127.92, 127.27, 126.49, 125.99, 125.82, 125.08, 124.23, 123.30, 118.62, 116.72, 116.33, 115.53, 113.42, 109.41, 39.89. HRMS (ESI) calculated for $C_{21}H_{17}BrN_3S^+$ $[M+H]^+$: 422.0321; found: 422.0315.

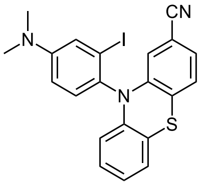

10-(4-(Dimethylamino)-2-iodophenyl)-10H-phenothiazine-2-carbonitrile (3ed): yellow solid was obtained in 87% isolated yield. ^{1}H NMR (400 MHz, DMSO-d_6) δ 7.33 (d, *J* = 2.8 Hz, 1H), 7.31 (d, *J* = 8.8 Hz, 1H), 7.21 (dd, *J* = 8.0, 1.6 Hz, 1H), 7.15 (d, *J* = 8.0 Hz, 1H), 7.00 – 6.94 (m, 2H), 6.93 – 6.87 (m, 1H), 6.82 (td, *J* = 7.6, 1.2 Hz, 1H), 6.05 (d, *J* = 1.6 Hz, 1H), 5.96 (dd, *J* = 8.0, 1.2 Hz, 1H), 2.99 (s, 6H). ^{13}C NMR (101 MHz, DMSO-d_6) δ 150.86, 142.86, 141.12, 131.79, 127.86,

127.23, 126.46, 125.93, 125.86, 123.25, 122.69, 118.67, 116.71, 116.44, 115.69, 114.13, 109.28, 102.82, 39.91. HRMS (ESI) calculated for $C_{21}H_{17}IN_3S^+$ $[M+H]^+$: 470.0182; found: 470.0183.

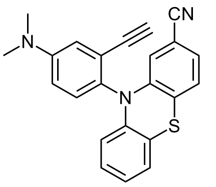

10-(4-(Dimethylamino)-2-ethynylphenyl)-10H-phenothiazine-2-carbonitrile (3fd): yellow solid was obtained in 90% isolated yield. ^{1}H NMR (400 MHz, DMSO-d_6) δ 7.27 (d, J = 8.8 Hz, 1H), 7.21 (dd, J = 8.0, 1.6 Hz, 1H), 7.16 (d, J = 7.6 Hz, 1H), 7.02 (d, J = 3.2 Hz, 1H), 7.00 – 6.95 (m, 2H), 6.94 – 6.88 (m, 1H), 6.82 (td, J = 7.4, 1.3 Hz, 1H), 6.12 (d, J = 1.2 Hz, 1H), 6.01 (dd, J = 8.4, 1.2 Hz, 1H), 4.18 (s, 1H), 3.01 (s, 6H). ^{13}C NMR (101 MHz, DMSO-d_6) δ 149.98, 143.70, 142.03, 131.54, 128.03, 127.88, 127.27, 126.47, 125.98, 125.77, 123.62, 123.12, 118.71, 116.91, 116.80, 116.51, 115.76, 114.89, 109.35, 84.74, 80.41, 39.92. HRMS (ESI) calculated for $C_{23}H_{18}N_3S^+$ $[M+H]^+$: 368.1216; found: 368.1214.

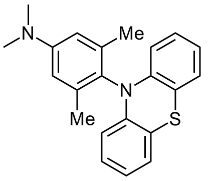

***N,N*,3,5-Tetramethyl-4-(10H-phenothiazin-10-yl)aniline (3ga):** white solid was obtained in 98% isolated yield. ^{1}H NMR (400 MHz, CDCl$_3$) δ 6.85 (dd, J = 7.2, 1.6 Hz, 2H), 6.75 (td, J = 7.7, 1. 6Hz, 2H), 6.68 (td, J = 7.2, 1.2 Hz, 2H), 6.57 (s, 2H), 5.94 (dd, J = 8.0, 1.2 Hz, 2H), 2.99 (s, 6H), 2.15 (s, 6H). ^{13}C NMR (101 MHz, CDCl$_3$) δ 149.92, 142.00, 138.60, 127.10, 126.69, 126.11, 121.70, 118.21, 114.11, 112.69, 40.50, 18.45. HRMS (ESI) calculated for $C_{22}H_{23}N_2S^+$ $[M+H]^+$: 347.1576; found: 347.1570.

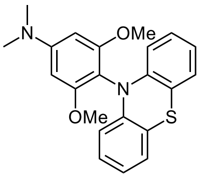

3,5-Dimethoxy-N,N-dimethyl-4-(10H-phenothiazin-10-yl)aniline (3ha): white solid was obtained in 97% isolated yield. ^{1}H NMR (400 MHz, CDCl$_3$) δ 6.90 (dd, J = 7.2, 1.6 Hz, 2H), 6.80 (td, J = 8.0, 1.6 Hz, 2H), 6.71 (t, J = 7.2 Hz, 2H), 6.14 (d, J = 8.4 Hz, 2H), 6.01 (s, 2H), 3.75 (s, 6H), 3.06 (s, 6H). ^{13}C NMR (101 MHz, CDCl$_3$) δ 158.62, 151.63, 143.56, 126.82, 126.16, 121.58, 119.41,

115.00, 106.93, 89.48, 55.90, 40.60. HRMS (ESI) calculated for $C_{22}H_{23}N_2O_2S^+$ $[M+H]^+$: 379.1475; found: 379.1472.

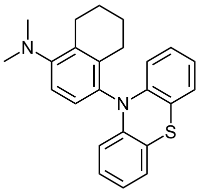

***N,N*-Dimethyl-4-(10H-phenothiazin-10-yl)-5,6,7,8-tetrahydronaphthalen-1-amine (3ia):** white solid was obtained in 63% isolated yield. ^{1}H NMR (400 MHz, $CDCl_3$) δ 7.17 – 7.01 (m, 2H), 6.91 (dd, *J* = 7.6, 1.6 Hz, 2H), 6.78 (td, *J* = 8.0, 2.0 Hz, 2H), 6.72 (td, *J* = 7.6, 1.2 Hz, 2H), 6.06 (dd, *J* = 8.0, 1.2 Hz, 2H), 2.79 (t, *J* = 8.4 Hz, 2H), 2.76 (s, 6H), 2.69 – 2.64 (m, 2H), 1.73 – 1.66 (m, 4H). ^{13}C NMR (101 MHz, $CDCl_3$) δ 152.44, 143.01, 137.54, 134.79, 132.97, 128.76, 126.87, 126.29, 121.89, 118.85, 116.96, 114.85, 44.34, 26.19, 24.91, 22.77, 22.42. HRMS (ESI) calculated for $C_{24}H_{25}N_2S^+$ $[M+H]^+$:373.1733; found: 373.1729.

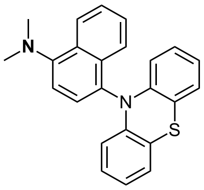

***N,N*-Dimethyl-2-(10H-phenothiazin-10-yl)naphthalen-1-amine (3ja):** white solid was obtained in 75% isolated yield. ^{1}H NMR (400 MHz, $CDCl_3$) δ 8.32 (d, *J* = 8.4 Hz, 1H), 8.02 (d, *J* = 8.4 Hz, 1H), 7.61 – 7.46 (m, 2H), 7.44 – 7.37 (m, 1H), 7.18 (d, *J* = 7.9 Hz, 1H), 6.99 (dd, *J* = 7.3, 1.8 Hz, 2H), 6.88 – 6.74 (td, *J* = 7.2, 1.6 Hz, 2H), 6.69 (td, *J* = 8.4, 2.0 Hz, 2H), 6.04 (dd, *J* = 7.6, 1.2 Hz, 2H), 2.99 (s, 6H). ^{13}C NMR (101 MHz, $CDCl_3$) δ 151.50, 144.02, 132.35, 131.52, 130.28, 129.20, 126.92, 126.85, 126.40, 125.76, 125.07, 124.11, 122.19, 119.44, 115.77, 114.15, 45.23. HRMS (ESI) calculated for $C_{24}H_{21}N_2S^+$ $[M+H]^+$: 369.1420; found: 369.1417.

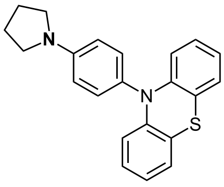

10-(4-(Pyrrolidin-1-yl)phenyl)-10H-phenothiazine (3ka)： white solid was obtained in 65% isolated yield. ^{1}H NMR (400 MHz, $CDCl_3$) δ 7.23 – 7.15 (m, 2H), 6.96 (dd, *J* = 7.6, 1.6 Hz, 2H), 6.85 – 6.78 (m, 2H), 6.77 – 6.69 (m, 4H), 6.26 (dd, *J* = 8.0, 1.2 Hz, 2H), 3.65 – 2.99 (m, 4H), 2.18 – 1.99 (m, 4H). ^{13}C NMR (101 MHz, $CDCl_3$) δ 147.34, 145.11, 131.69, 128.07, 126.74, 126.40,

121.87, 119.32, 115.65, 112.89, 47.67, 25.59. HRMS (ESI) calculated for $C_{22}H_{21}N_2S^+$ $[M+H]^+$: 345.1420; found: 345.1414.

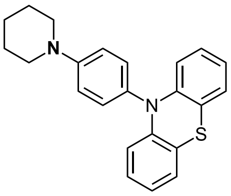

10-(4-(Piperidin-1-yl)phenyl)-10H-phenothiazine (3la): white solid was obtained in 72% isolated yield. ^1H NMR (400 MHz, $CDCl_3$) δ 7.24 – 7.19 (m, 2H), 7.17 – 7.06 (m, 2H), 6.97 (dd, *J* = 7.2, 1.6 Hz, 2H), 6.82 (td, *J* = 8.0, 1.2 Hz, 2H), 6.76 (td, *J* = 7.2, 1.2 Hz, 2H), 6.23 (dd, *J* = 8.0, 1.2 Hz, 2H), 3.26 (t, *J* = 5.6 Hz, 4H), 1.80 – 1.72 (m, 4H), 1.67 – 1.59 (m, 2H). ^{13}C NMR (101 MHz, $CDCl_3$) δ 151.58, 144.81, 131.57, 131.18, 126.75, 126.46, 122.02, 119.41, 117.50, 115.64, 50.07, 25.80, 24.22. HRMS (ESI) calculated for $C23H_{23}N_2S^+$ $[M+H]^+$: 359.1576; found: 359.1573.

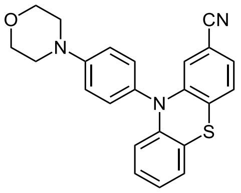

10-(4-Morpholinophenyl)-10H-phenothiazine-2-carbonitrile (3md): yellow solid was obtained in 52% isolated yield. ^1H NMR (400 MHz, $CDCl_3$) δ 7.24 – 7.18 (m, 2H), 7.14 – 7.08 (m, 2H), 7.02 – 6.94 (m, 2H), 6.92 (dd, *J* = 7.2, 2.0 Hz, 1H), 6.87 – 6.76 (m, 2H), 6.31 (d, *J* = 1.2 Hz, 1H), 6.18 (dd, *J* = 8.0, 1.6 Hz, 1H), 3.92 (t, *J* = 4.8 Hz, 4H), 3.30 (t, *J* = 4.8 Hz, 4H). ^{13}C NMR (101 MHz, $CDCl_3$) δ 151.16, 145.14, 143.38, 131.29, 130.62, 127.43, 126.73, 126.66, 126.50, 125.46, 122.98, 119.00, 117.87, 117.55, 117.24, 116.02, 110.05, 66.79, 48.58. HRMS (ESI) calculated for $C23H_{23}N_2S^+$ $[M+H]^+$: 359.1576; found: 359.1573. HRMS (ESI) calculated for $C_{23}H_{20}N_3OS^+$ $[M+H]^+$: 386.1322; found: 386.1318.

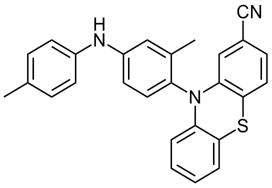

10-(2-Methyl-4-(p-tolylamino)phenyl)-10H-phenothiazine-2-carbonitrile (4a): yellow solid was obtained in 93% isolated yield. ^1H NMR (400 MHz, DMSO-d_6) δ 8.35 (s, 1H), 7.24 – 7.05 (m, 9H), 7.00 (dd, *J* = 7.6, 1.6 Hz, 1H), 6.95 – 6.91 (m, 1H), 6.84 (td, *J* = 7.6, 1.2 Hz, 1H), 6.17 (d, *J* = 1.6 Hz, 1H), 6.11 – 6.02 (dd, *J* = 8.0, 0.6 Hz, 1H), 2.27 (s, 3H), 2.05 (s, 3H). ^{13}C NMR (101 MHz,

DMSO-d_6) δ 145.07, 143.40, 141.62, 139.67, 137.76, 131.37, 129.94, 129.71, 128.06, 127.60, 127.29, 126.55, 125.86, 125.75, 123.17, 119.02, 118.66, 117.75, 116.77, 116.06, 115.37, 114.84, 109.64, 20.39, 17.30. HRMS (ESI) calculated for $C_{27}H_{22}N_3S^+$ [M+H]$^+$: 420.1529; found: 420.1532.

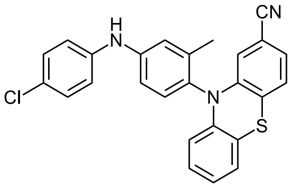

10-(4-((4-Chlorophenyl)amino)-2-methylphenyl)-10H-phenothiazine-2-carbonitrile (4b): yellow solid was obtained in 91% isolated yield. ^{1}H NMR (400 MHz, DMSO-d_6) δ 8.61 (s, 1H), 7.35 – 7.28 (m, 2H), 7.25 – 7.10 (m, 7H), 6.99 (dd, J = 7.2, 1.6 Hz, 1H), 6.95 – 6.88 (m, 1H), 6.83 (td, J = 7.2, 1.2 Hz, 1H), 6.15 (d, J = 1.6 Hz, 1H), 6.04 (dd, J = 8.0, 1.2 Hz, 1H), 2.06 (s, 3H). ^{13}C NMR (101 MHz, DMSO-d_6) δ 143.82, 143.30, 141.58, 141.53, 138.04, 131.59, 129.11, 128.74, 128.09, 127.34, 126.61, 125.96, 125.81, 123.74, 123.25, 119.23, 119.10, 118.66, 116.84, 116.07, 115.37, 109.68, 17.30. HRMS (EI) calculated for $C_{26}H_{18}ClN_3S^+$ [M]$^+$: 439.0904; found: 439.0899.

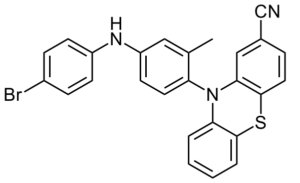

10-(4-((4-Bromophenyl)amino)-2-methylphenyl)-10H-phenothiazine-2-carbonitrile (4c): yellow solid was obtained in 83% isolated yield. ^{1}H NMR (400 MHz, $CDCl_3$) δ 7.47 – 7.40 (m, 2H), 7.17 – 7.02 (m, 5H), 7.00 – 6.91 (m, 2H), 6.90 – 6.75 (m, 3H), 6.21 (d, J = 1.6 Hz, 1H), 6.08 (dd, J = 8.0, 1.4 Hz, 1H), 5.88 (s, 1H), 2.13 (s, 3H). ^{13}C NMR (101 MHz, $CDCl_3$) δ 143.68, 143.63, 141.84, 140.95, 139.09, 132.33, 131.77, 130.13, 127.63, 126.66, 126.45, 126.40, 125.51, 122.99, 120.75, 119.80, 119.04, 117.46, 116.73, 116.39, 115.36, 114.10, 110.12, 17.75. HRMS (ESI) calculated for $C_{26}H_{19}BrN_3S^+$ [M+H]$^+$: 484.0478; found: 484.0450.

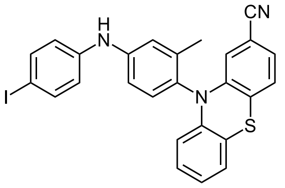

10-(4-((4-Iodophenyl)amino)-2-methylphenyl)-10H-phenothiazine-2-carbonitrile (4d): yellow solid was obtained in 84% isolated yield. ^{1}H NMR (400 MHz, DMSO-d_6) δ 8.61 (s, 1H), 7.62 – 7.53 (m, 2H), 7.30 – 7.10 (m, 5H), 7.07 – 6.97 (m, 3H), 6.96 – 6.89 (m, 1H), 6.84 (td, J = 7.2, 1.2

Hz, 1H), 6.15 (d, *J* = 1.6 Hz, 1H), 6.04 (dd, *J* = 8.4, 1.2 Hz, 1H), 2.06 (s, 3H). ^{13}C NMR (101 MHz, DMSO-d_6) δ 143.55, 143.28, 142.47, 141.51, 138.03, 137.74, 131.60, 128.86, 128.11, 127.36, 126.62, 125.98, 125.80, 123.26, 119.92, 119.30, 118.66, 116.83, 116.29, 116.08, 115.37, 109.68, 82.31, 17.30. HRMS (ESI) calculated for $C_{26}H_{19}IN_3S^+$ $[M+H]^+$: 532.0339; found: 532.0315.

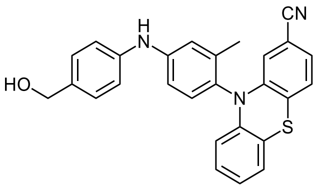

10-(4-((4-(Hydroxymethyl)phenyl)amino)-2-methylphenyl)-10H-phenothiazine-2-carbonitrile (4e): yellow solid was obtained in 75% isolated yield. ^{1}H NMR (400 MHz, $CDCl_3$) δ 7.37 – 7.31 (m, 2H), 7.20 (dt, *J* = 8.4, 2.0 Hz, 2H), 7.10 – 7.05 (m, 3H), 6.97 (dd, *J* = 8.0, 1.6 Hz, 1H), 6.91 (d, *J* = 7.6 Hz, 1H), 6.89 – 6.84 (m, 1H), 6.82 (dd, *J* = 7.9, 1.9 Hz, 1H), 6.77 (td, *J* = 7.6, 1.2 Hz, 1H), 6.23 (d, *J* = 1.6 Hz, 1H), 6.09 (dd, *J* = 8.0, 1.2 Hz, 1H), 6.01 (s, 1H), 4.65 (s, 2H), 2.12 (s, 3H). ^{13}C NMR (101 MHz, $CDCl_3$) δ 144.17, 143.72, 141.88, 141.29, 138.86, 134.54, 131.59, 129.58, 128.53, 127.60, 126.60, 126.38, 126.32, 125.43, 122.91, 119.43, 119.36, 119.03, 117.38, 116.71, 116.00, 115.37, 110.06, 64.93, 17.71. HRMS (EI) calculated for $C_{27}H_{21}N_3OS^+$ $[M]^+$: 435.1400; found: 435.1397.

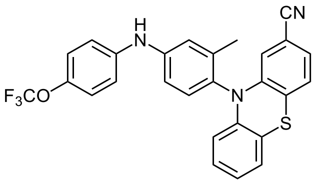

10-(2-Methyl-4-((4-(trifluoromethoxy)phenyl)amino)phenyl)-10H-phenothiazine-2-carbonitrile (4f): yellow solid was obtained in 91% isolated yield. ^{1}H NMR (400 MHz, DMSO-d_6) δ 8.68 (s, 1H), 7.32 – 7.14 (m, 9H), 7.01 (dd, *J* = 7.6, 1.6 Hz, 1H), 6.96 – 6.90 (m, 1H), 6.85 (td, *J* = 7.6, 1.2 Hz, 1H), 6.17 (d, *J* = 1.6 Hz, 1H), 6.07 (dd, *J* = 8.4, 1.2 Hz, 1H), 2.09 (s, 3H). ^{13}C NMR (101 MHz, DMSO-d_6) δ 143.76, 143.28, 141.99, 141.51, 138.07, 131.59, 128.89, 128.06, 127.31, 126.59, 125.94, 125.81, 123.22, 122.31, 121.54, 119.22, 119.01, 118.63, 118.56, 116.85, 116.16, 116.06, 115.35, 109.68, 17.25. ^{19}F NMR (377 MHz, DMSO-d_6) δ -57.15. HRMS (EI) calculated for $C_{27}H_{18}F_3N_3OS^+$ $[M]^+$: 489.1117; found: 489.1113.

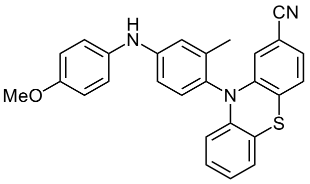

10-(4-((4-Methoxyphenyl)amino)-2-methylphenyl)-10H-phenothiazine-2-carbonitrile (4g): yellow solid was obtained in 74% isolated yield. ^{1}H NMR (400 MHz, $CDCl_3$) δ 7.23 – 7.17 (m, 2H), 7.06 – 7.00 (m, 1H), 6.98 – 6.90 (m, 5H), 6.90 – 6.84 (m, 2H), 6.84 – 6.74 (m, 2H), 6.23 (d, *J* = 1.6 Hz, 1H), 6.10 (dd, *J* = 8.0, 1.2 Hz, 1H), 5.69 (s, 1H), 3.83 (s, 3H), 2.09 (s, 3H). ^{13}C NMR (101 MHz, $CDCl_3$) δ 156.05, 146.26, 143.90, 142.06, 138.73, 134.32, 131.50, 128.47, 127.61, 126.57, 126.36, 126.27, 125.37, 123.96, 122.86, 119.12, 117.56, 117.36, 116.83, 115.45, 114.70, 114.34, 110.12, 55.52, 17.75. HRMS (ESI) calculated for $C_{27}H_{22}N_3OS^+$ $[M+H]^+$: 436.1478; found: 436.1474.

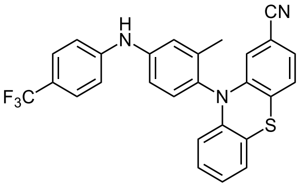

10-(2-Methyl-4-((4-(trifluoromethyl)phenyl)amino)phenyl)-10H-phenothiazine-2-carbonitrile (4h): yellow solid was obtained in 43% isolated yield. ^{1}H NMR (400 MHz, DMSO-d_6) δ 9.00 (s, 1H), 7.61 (d, *J* = 8.5 Hz, 2H), 7.37 – 7.24 (m, 5H), 7.24 (dd, *J* = 8.0, 1.6 Hz, 1H), 7.20 (d, *J* = 8.0 Hz, 1H), 7.03 (dd, *J* = 7.6, 1.6 Hz, 1H), 6.99 – 6.92 (m, 1H), 6.86 (td, *J* = 7.6, 1.2 Hz, 1H), 6.18 (d, *J* = 1.6 Hz, 1H), 6.07 (dd, *J* = 8.4, 1.2 Hz, 1H), 2.12 (s, 3H). ^{13}C NMR (101 MHz, DMSO-d_6) δ 147.01, 143.62, 142.97, 141.86, 138.68, 132.21, 130.45, 128.56, 127.82, 127.09, 127.04, 126.64, 126.48, 126.29, 123.95, 123.75, 121.35, 120.03, 119.72, 119.08, 118.23, 117.33, 116.54, 116.40, 115.80, 110.14, 17.69. ^{19}F NMR (377 MHz, DMSO) δ -59.63. HRMS (EI) calculated for $C_{27}H_{18}F_3N_3S^+$ $[M]^+$: 473.1168; found: 473.1161.

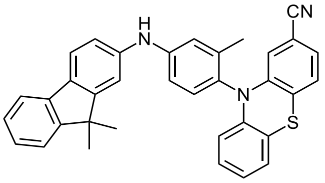

10-(4-((9,9-Dimethyl-9H-fluoren-2-yl)amino)-2-methylphenyl)-10H-phenothiazine-2-carbonitrile (4i): yellow solid was obtained in 66% isolated yield. ^{1}H NMR (400 MHz, DMSO-d_6) δ 8.62 (s, 1H), 7.74 (d, *J* = 8.0 Hz, 1H), 7.71 (d, *J* = 7.2 Hz, 1H), 7.50 (d, *J* = 7.2 Hz, 1H), 7.37 – 7.26 (m, 2H), 7.26 – 7.14 (m, 7H), 7.01 (dd, *J* = 7.6, 1.6 Hz, 1H), 6.98 – 6.90 (m, 1H), 6.84 (td, *J* =

7.6, 1.2 Hz, 1H), 6.18 (d, J = 1.6 Hz, 1H), 6.09 (dd, J = 8.0, 1.2 Hz, 1H), 2.07 (s, 3H), 1.44 (s, 6H). ^{13}C NMR (101 MHz, DMSO-d_6) δ 154.81, 152.81, 144.60, 143.40, 142.00, 141.62, 138.80, 137.90, 131.73, 131.56, 128.12, 127.36, 127.01, 126.62, 126.12, 125.94, 125.83, 123.24, 122.62, 120.96, 119.12, 118.69, 118.34, 117.31, 116.84, 116.14, 115.44, 115.37, 113.02, 109.68, 99.55, 46.41, 27.07, 17.37. HRMS (ESI) calculated for $C_{35}H_{28}N_3S^+$ [M+H]$^+$: 522.1999; found: 522.1993.

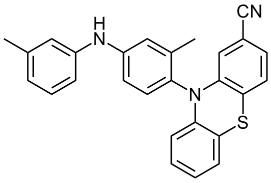

10-(2-Methyl-4-(m-tolylamino)phenyl)-10H-phenothiazine-2-carbonitrile (4j): yellow solid was obtained in 66% isolated yield. ^{1}H NMR (400 MHz, DMSO-d_6) δ 8.40 (s, 1H), 7.26 – 7.07 (m, 6H), 7.06 – 6.96 (m, 3H), 6.95 – 6.89 (m, 1H), 6.83 (td, J = 7.6, 1.2 Hz, 1H), 6.73 (d, J = 7.2 Hz, 1H), 6.15 (d, J = 1.6 Hz, 1H), 6.06 (dd, J = 8.4, 1.6 Hz, 1H), 2.28 (s, 3H), 2.05 (s, 3H). ^{13}C NMR (101 MHz, DMSO-d_6) δ 144.56, 143.36, 142.32, 141.58, 138.49, 137.82, 131.41, 129.10, 128.07, 128.01, 127.30, 126.57, 125.89, 125.77, 123.19, 121.63, 118.87, 118.66, 118.52, 116.80, 116.07, 115.37, 109.65, 21.24, 17.30. HRMS (ESI) calculated for $C_{27}H_{22}N_3S^+$ [M+H]$^+$: 420.1529; found: 420.1535.

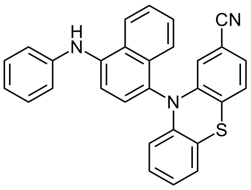

10-(4-(Phenylamino)naphthalen-1-yl)-10H-phenothiazine-2-carbonitrile (4k): yellow solid was obtained in 99% isolated yield. ^{1}H NMR (400 MHz, DMSO-d_6) δ 8.59 (s, 1H), 8.51-8.47 (m, 1H), 7.93 – 7.87 (m, 1H), 7.68 – 7.52 (m, 3H), 7.43 (d, J = 8.0 Hz, 1H), 7.39 -7.29 (m, 4H), 7.28 – 7.18 (m, 2H), 7.13 – 7.02 (m, 1H), 7.02 – 6.94 (m, 1H), 6.90 – 6.77 (m, 2H), 6.20 (d, J = 1.6 Hz, 1H), 6.12 – 5.97 (m, 1H). ^{13}C NMR (101 MHz, DMSO-d_6) δ 144.03, 143.25, 142.37, 141.51, 130.84, 130.11, 129.28, 127.99, 127.94, 127.46, 127.34, 126.66, 126.58, 126.41, 126.16, 125.85, 123.91, 123.42, 122.29, 121.31, 119.51, 118.52, 117.45, 116.82, 116.09, 110.85, 109.57. HRMS (ESI) calculated for $C_{29}H_{20}N_3S^+$ [M+H]$^+$:442.1373; found: 442.1351.

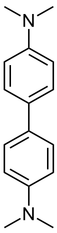

***N*[4],*N*[4],*N*[4'],*N*[4']-tetramethyl-[1,1'-biphenyl]-4,4'-diamine (5a):**[6] pale grey solid was obtained in 21% yield during the reaction between **1a** and **2h** with HFIP/MeCN as co-solvents. ^{1}H NMR (400 MHz, Chloroform-*d*) δ 7.48 – 7.43 (m, 4H), 6.84 – 6.78 (m, 4H), 2.97 (s, 12H). ^{13}C NMR (101 MHz, $CDCl_3$) δ 149.20, 129.78, 126.93, 113.04, 40.78.

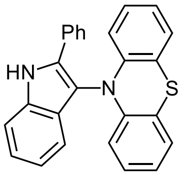

10-(2-Phenyl-1H-indol-3-yl)-10H-phenothiazine (6a):[7] white solid was obtained in 49% yield. ^{1}H NMR (400 MHz, DMSO-d_6) δ 11.95 (s, 1H), 8.07 – 7.85 (m, 2H), 7.58 (dt, *J* = 8.0, 1.0 Hz, 1H), 7.46 – 7.40 (m, 2H), 7.35 – 7.28 (m, 1H), 7.28 – 7.21 (m, 2H), 7.13 – 7.03 (m, 3H), 6.92 – 6.77 (m, 4H), 6.34 – 6.24 (m, 2H). ^{13}C NMR (101 MHz, DMSO) δ 143.47, 135.13, 133.01, 130.52, 128.94, 128.22, 127.53, 126.66, 126.21, 125.75, 122.83, 122.78, 120.32, 119.64, 118.02, 115.72, 112.65, 112.49.

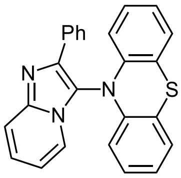

10-(2-Phenylimidazo[1,2-a]pyridin-3-yl)-10H-phenothiazine (6b): pale red solid was obtained in 76% yield. ^{1}H NMR (400 MHz, DMSO-d_6) δ 8.14 – 8.08 (m, 3H), 7.80 (dt, *J* = 9.2, 1.2 Hz, 1H), 7.49 – 7.37 (m, 3H), 7.34 – 7.27 (m, 1H), 7.24 – 7.17 (m, 2H), 7.01 (td, *J* = 6.8, 1.2 Hz, 1H), 6.97 – 6.87 (m, 4H), 6.09 – 6.00 (m, 2H). ^{13}C NMR (101 MHz, DMSO) δ 142.90, 140.50, 139.01, 132.54, 128.87, 128.49, 128.14, 127.22, 126.68, 126.30, 124.19, 123.00, 120.61, 117.90, 117.34, 115.26, 113.73. HRMS (ESI) calculated for $C_{25}H_{18}N_3S^+$ $[M+H]^+$: 392.1216; found: 392.1208.

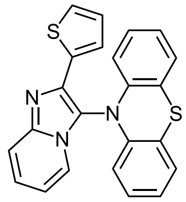

10-(2-(Thiophen-2-yl)imidazo[1,2-a]pyridin-3-yl)-10H-phenothiazine (6c): white solid was obtained in 57% yield. ^{1}H NMR (400 MHz, DMSO-d_6) δ 8.12 (dt, *J* = 6.8, 1.2 Hz, 1H), 7.77 (dt, *J* = 9.2, 1.2 Hz, 1H), 7.62 – 7.56 (m, 2H), 7.48 – 7.43 (m, 1H), 7.23 – 7.18 (m, 2H), 7.10 (dd, *J* = 5.2, 3.6 Hz, 1H), 7.02 (td, *J* = 6.8, 1.2 Hz, 1H), 6.97 – 6.88 (m, 4H), 6.05 – 6.00 (m, 2H). ^{13}C NMR (101 MHz, DMSO) δ 143.03, 140.31, 135.56, 135.34, 128.23, 128.12, 127.16, 126.86, 124.88, 124.18, 123.13, 120.59, 117.58, 116.13, 115.10, 113.83. HRMS (ESI) calculated for $C_{23}H_{16}N_3S_2^+$ $[M+H]^+$: 398.0780; found: 398.0778.

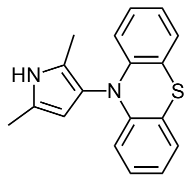

10-(2,5-Dimethyl-1H-pyrrol-3-yl)-10H-phenothiazine (6d): white solid was obtained in 23% yield. ^{1}H NMR (400 MHz, DMSO-d_6) δ 10.71 (s, 1H), 7.00 – 6.95 (m, 2H), 6.94 – 6.91 (m, 2H), 6.79 (td, *J* = 7.6, 1.2 Hz, 2H), 6.48 (dd, J = 8.4, 1.2 Hz, 2H), 5.71 (dd, J = 2.8, 1.2 Hz, 1H), 2.22 (s, 3H), 1.93 (s, 3H). 13C NMR (101 MHz, DMSO) δ 144.65, 127.28, 126.28, 125.45, 122.26, 122.07, 118.83, 118.81, 115.75, 104.55, 13.14, 9.95. HRMS (ESI) calculated for $C_{18}H_{15}N_2S^-$ $[M-H]^-$: 291.0961; found: 291.0953.

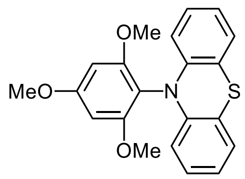

10-(2,4,6-Trimethoxyphenyl)-10H-phenothiazine (6e): white solid was obtained in 21% yield. ^{1}H NMR (400 MHz, DMSO-d_6) δ 6.90 (dd, *J* = 7.6, 1.6 Hz, 2H), 6.86 – 6.81 (m, 2H), 6.74 (td, *J* = 7.2, 1.2 Hz, 2H), 6.45 (s, 2H), 5.97 (dd, *J* = 8.4, 1.2 Hz, 2H), 3.87 (s, 3H), 3.71 (s, 6H). ^{13}C NMR (101 MHz, DMSO) δ 161.05, 158.05, 142.38, 127.32, 126.08, 121.98, 118.19, 114.62, 91.77, 55.93, 55.58. HRMS (ESI) calculated for $C_{21}H_{20}NO_3S^+$ $[M+H]^+$: 366.1158; found: 366.1151.

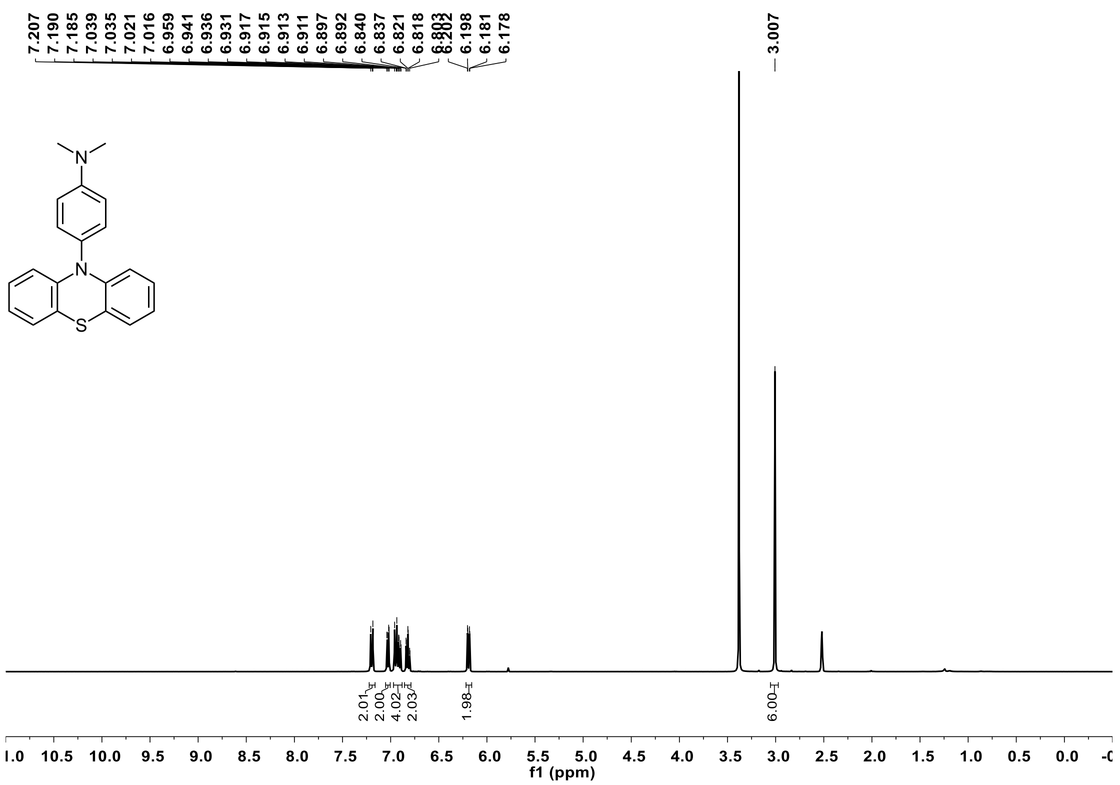

Supplementary Figure 3. ^{1}H NMR (400 MHz, DMSO-d$_6$) spectrum of 3aa

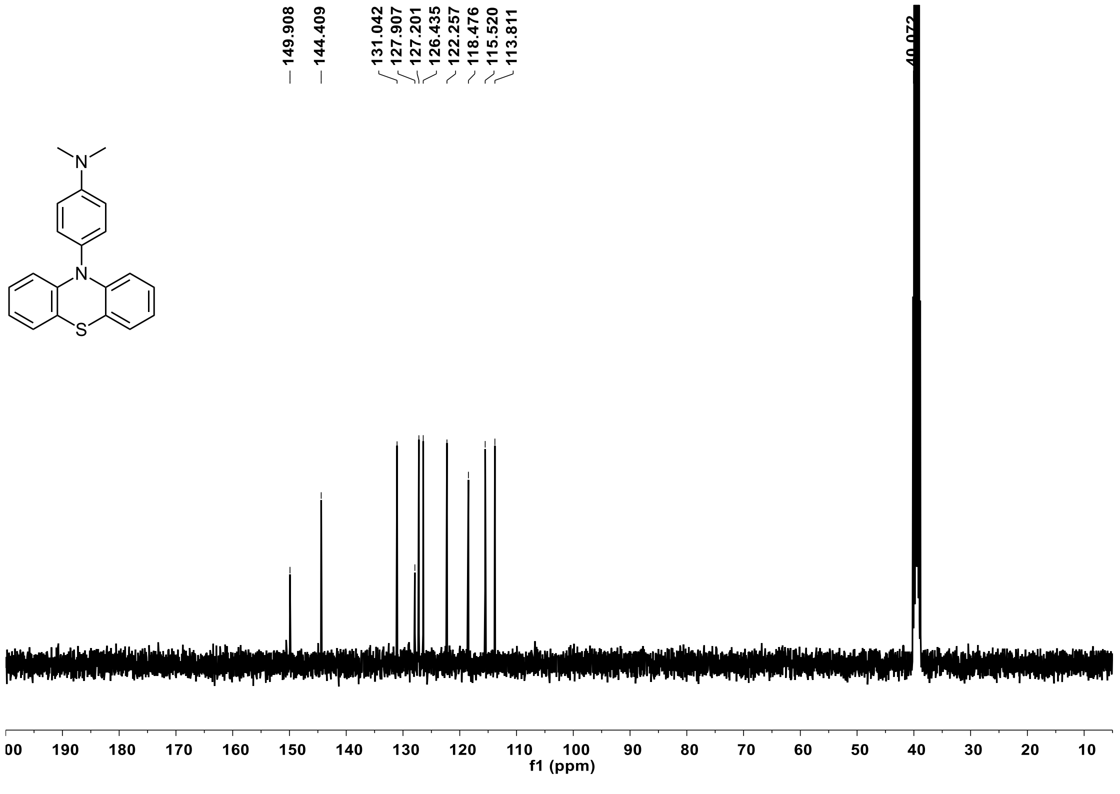

Supplementary Figure 4. ^{13}C NMR (101 MHz, DMSO-d$_6$) spectrum of 3aa

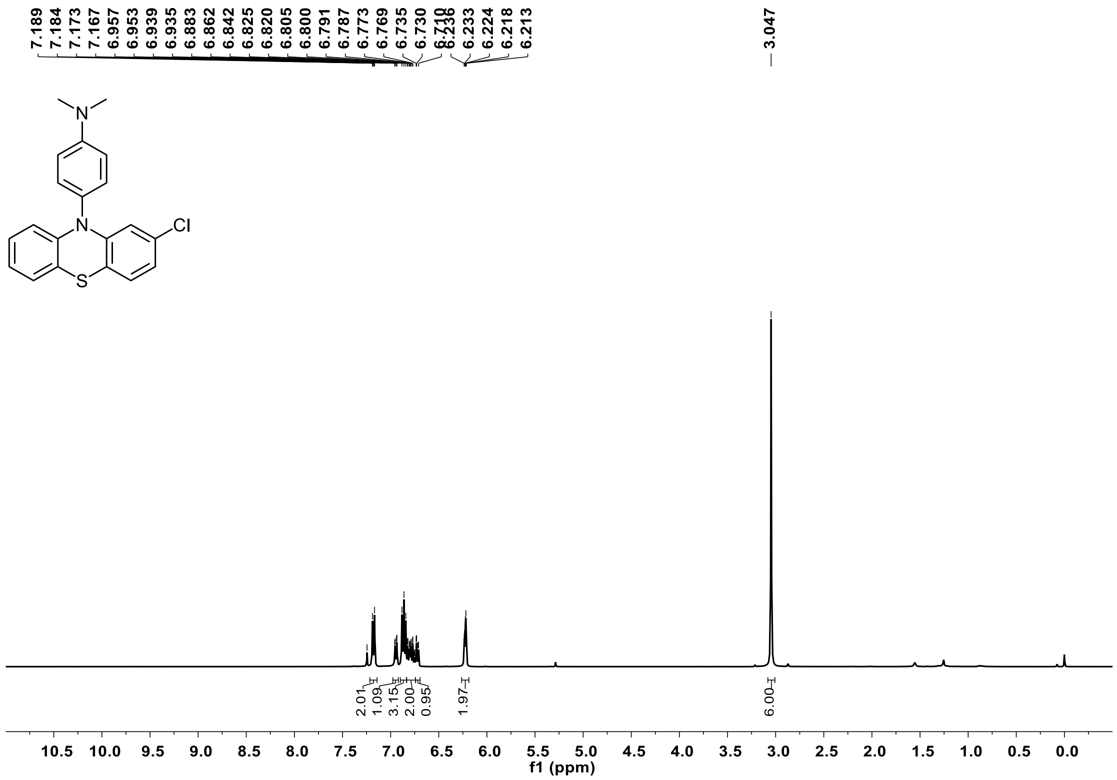

Supplementary Figure 5. ^{1}H NMR (400 MHz, $CDCl_3$) spectrum of 3ab

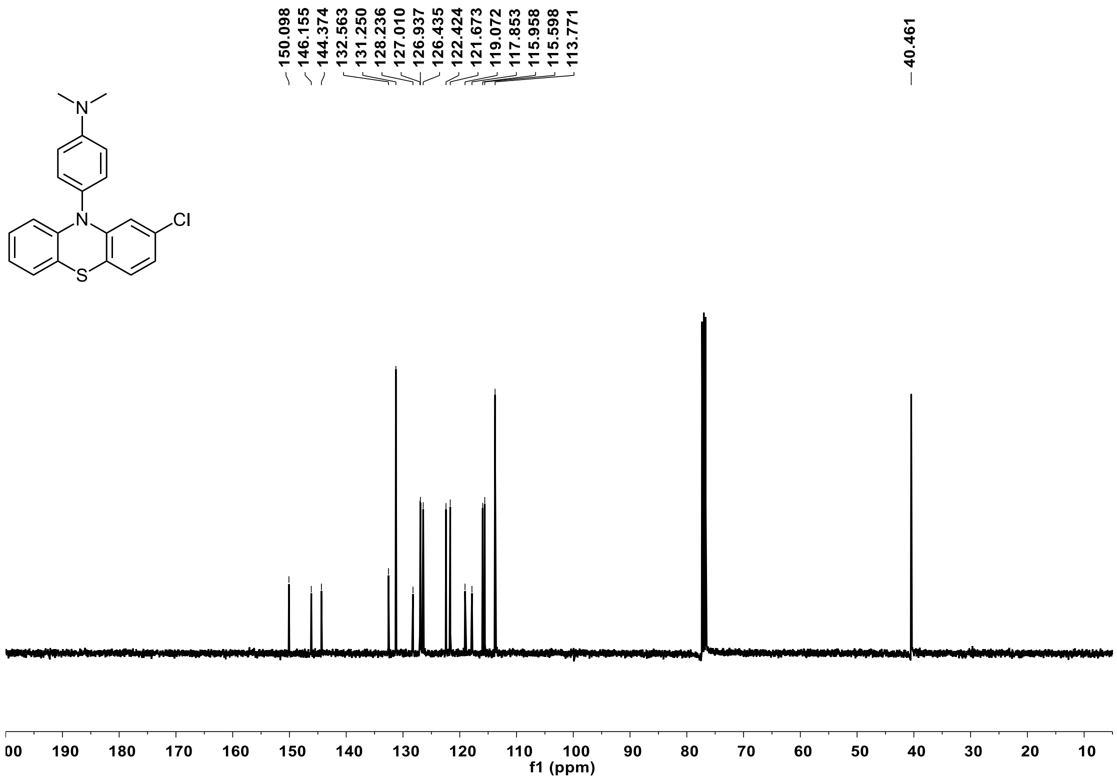

Supplementary Figure 6. ^{13}C NMR (101 MHz, $CDCl_3$) spectrum of 3ab

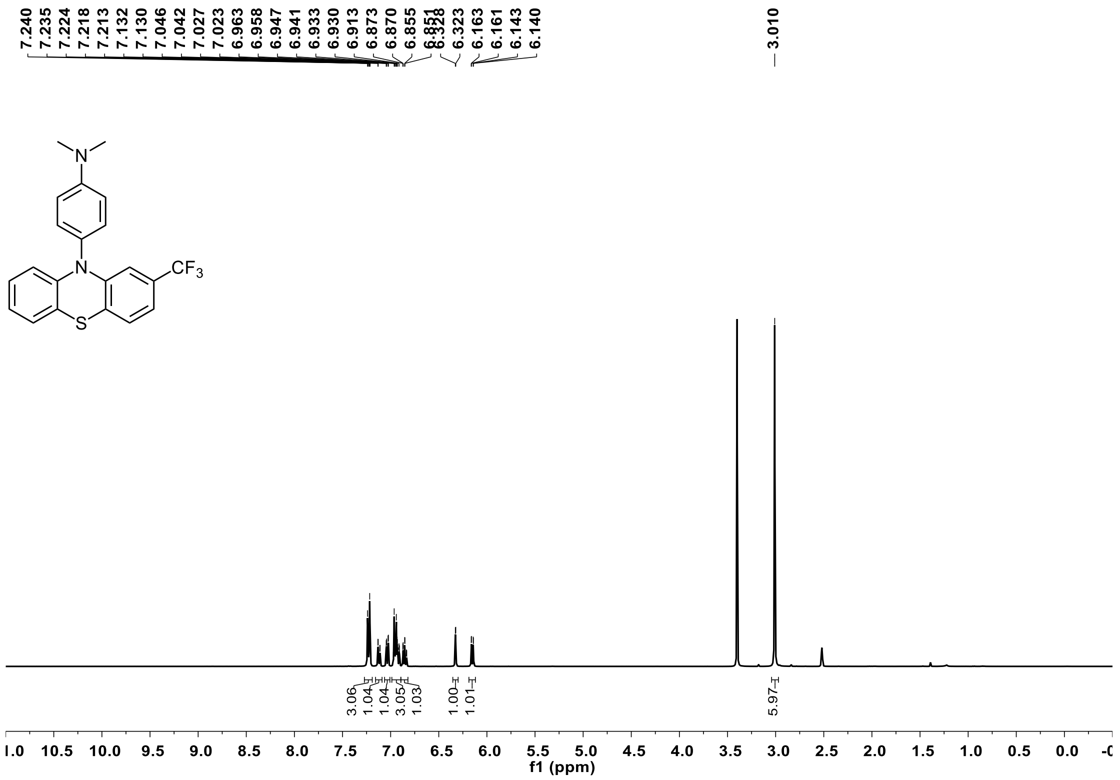

Supplementary Figure 7. **^{1}H NMR (400 MHz, DMSO-d$_6$) spectrum of 3ac**

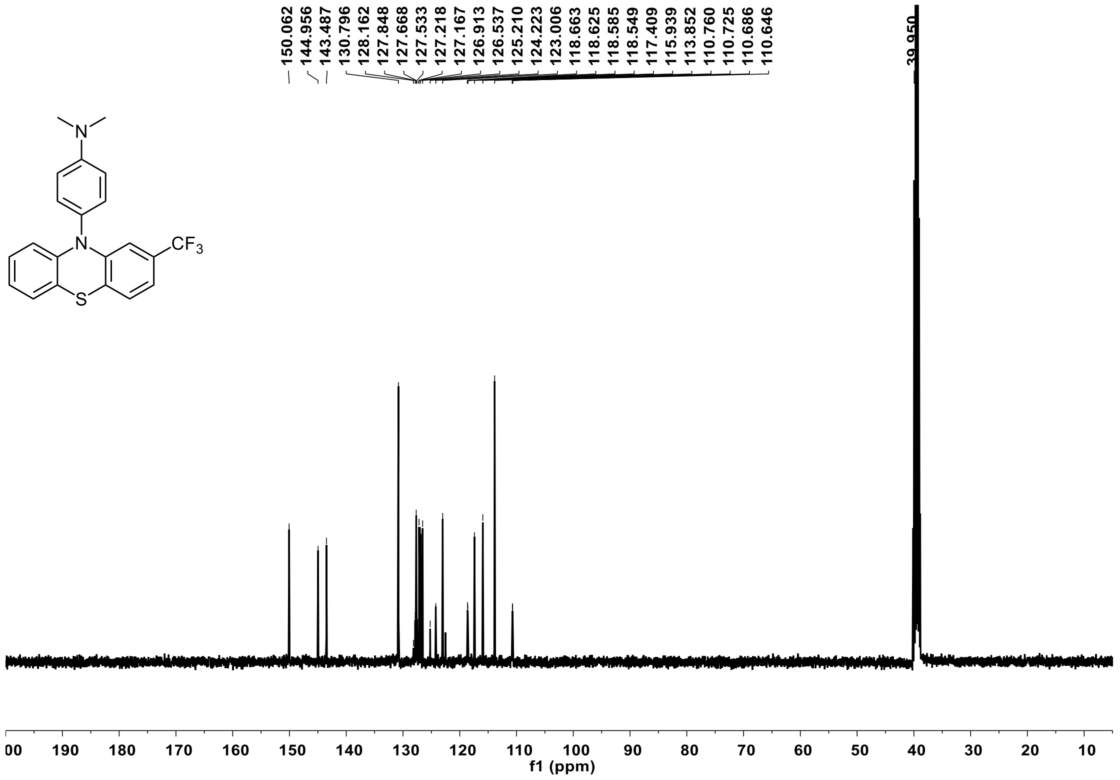

Supplementary Figure 8. **^{13}C NMR (101 MHz, DMSO-d$_6$) spectrum of 3ac**

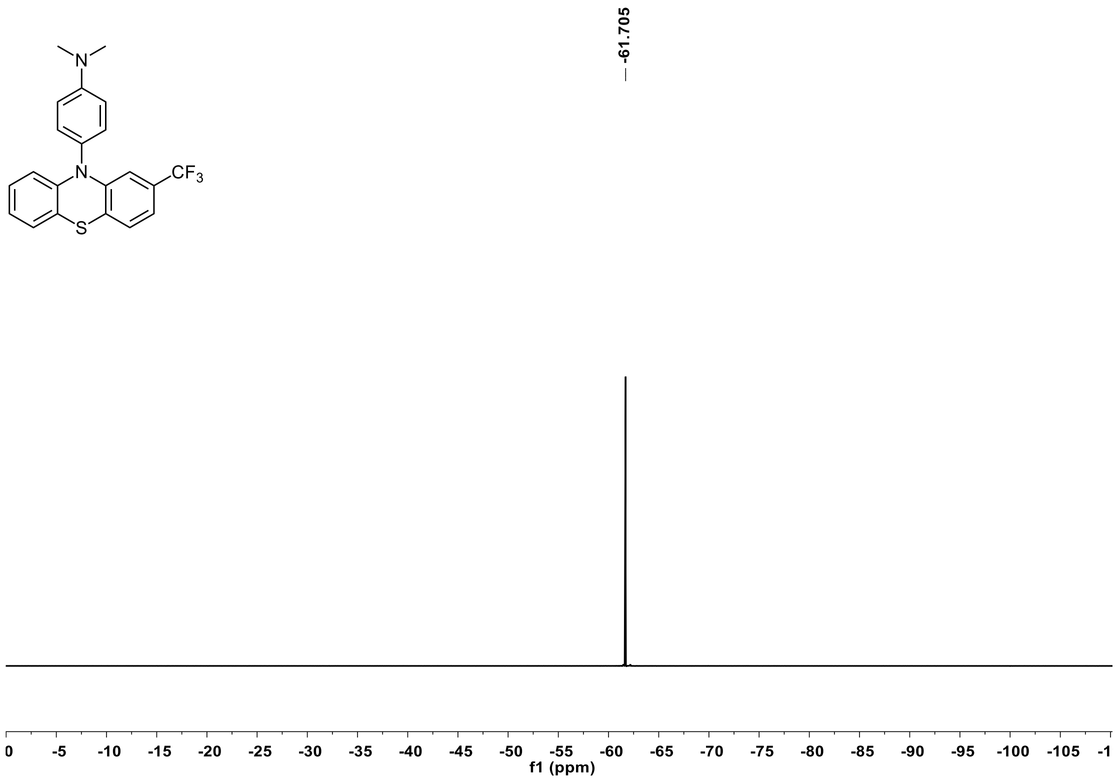

Supplementary Figure 9. ^{19}F NMR (377 MHz, DMSO-d_6) spectrum of 3ac

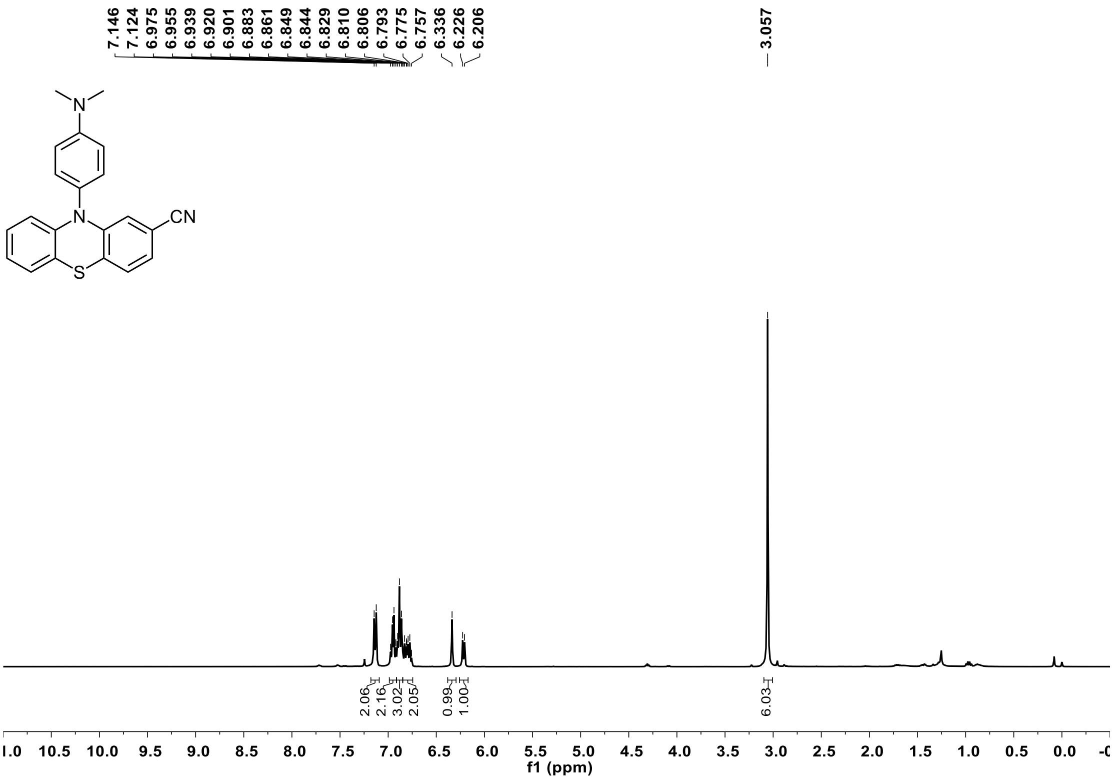

Supplementary Figure 10. ^{1}H NMR (400 MHz, $CDCl_3$) spectrum of 3ad

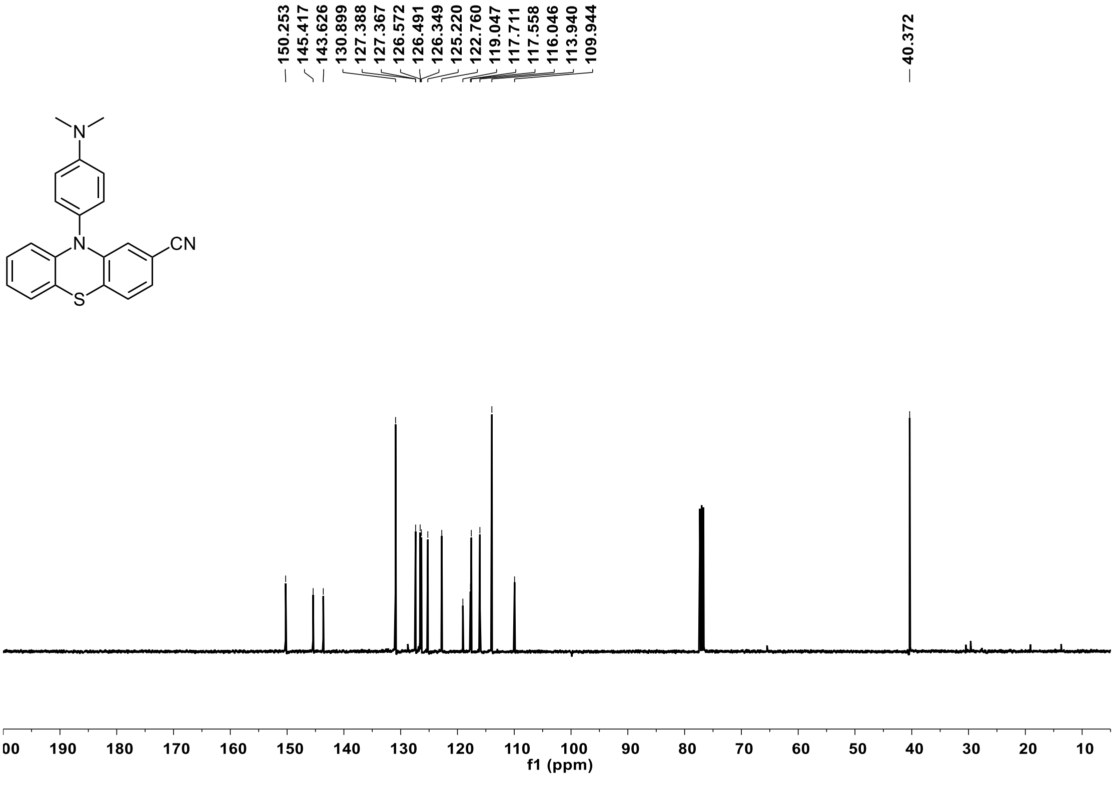

Supplementary Figure 11. ^{13}C NMR (101 MHz, $CDCl_3$) spectrum of 3ad

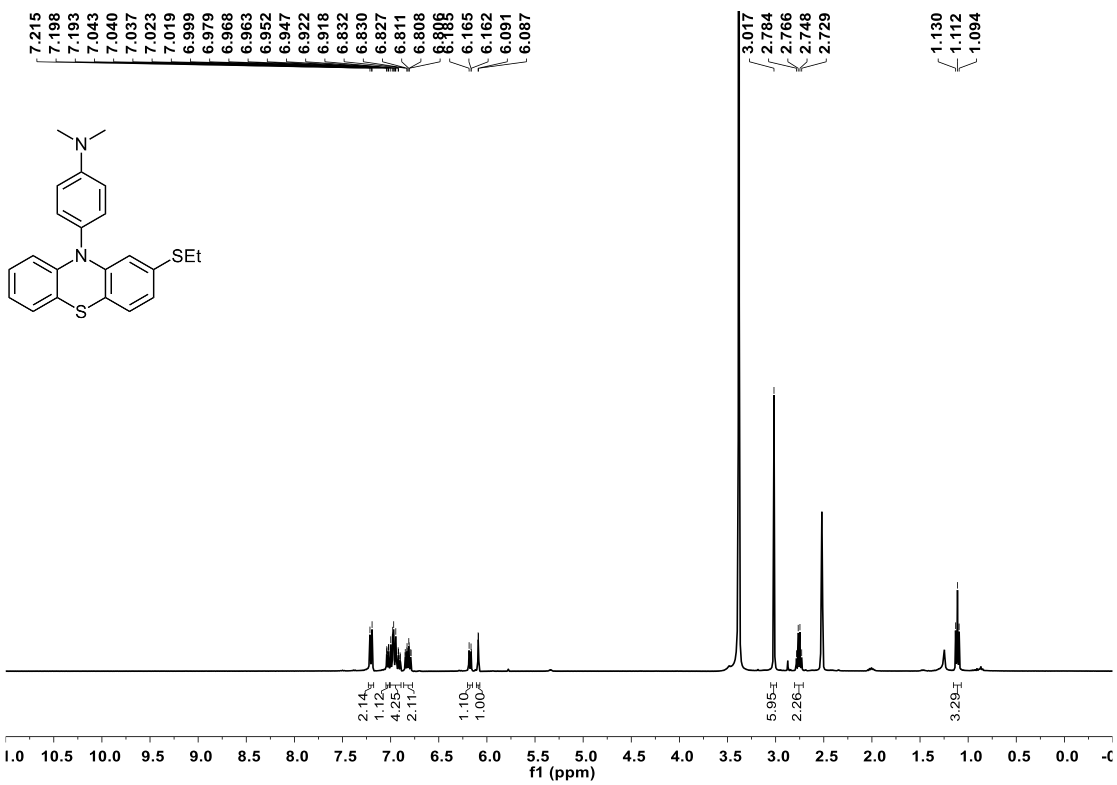

Supplementary Figure 12. ^{1}H NMR (400 MHz, DMSO-d_6) spectrum of 3ae

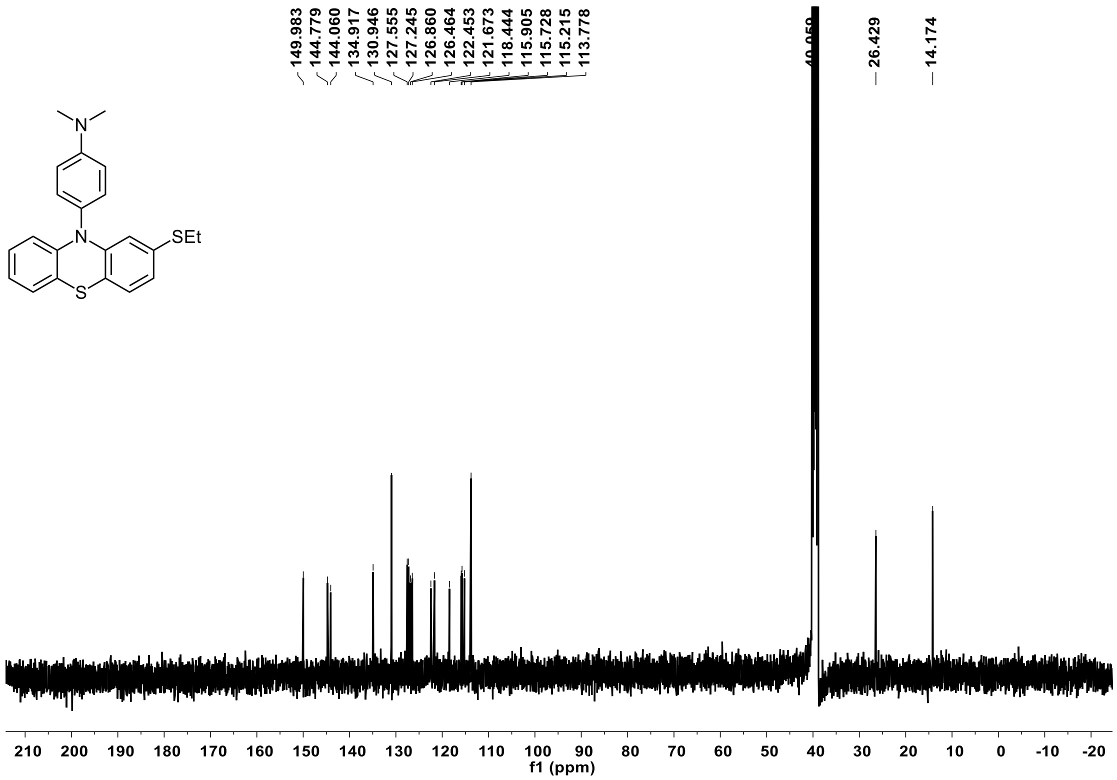

Supplementary Figure 13. ^{13}C NMR (101 MHz, DMSO-d_6) spectrum of 3ae

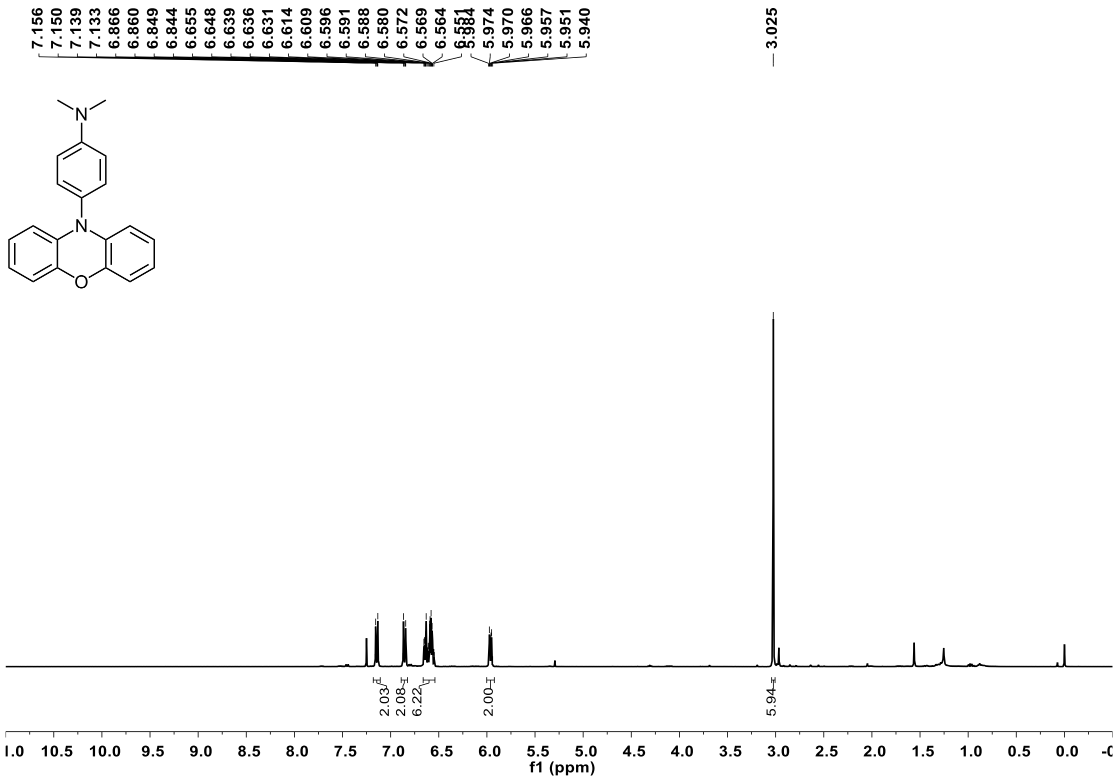

Supplementary Figure 14. ^{1}H NMR (400 MHz, $CDCl_3$) spectrum of 3af

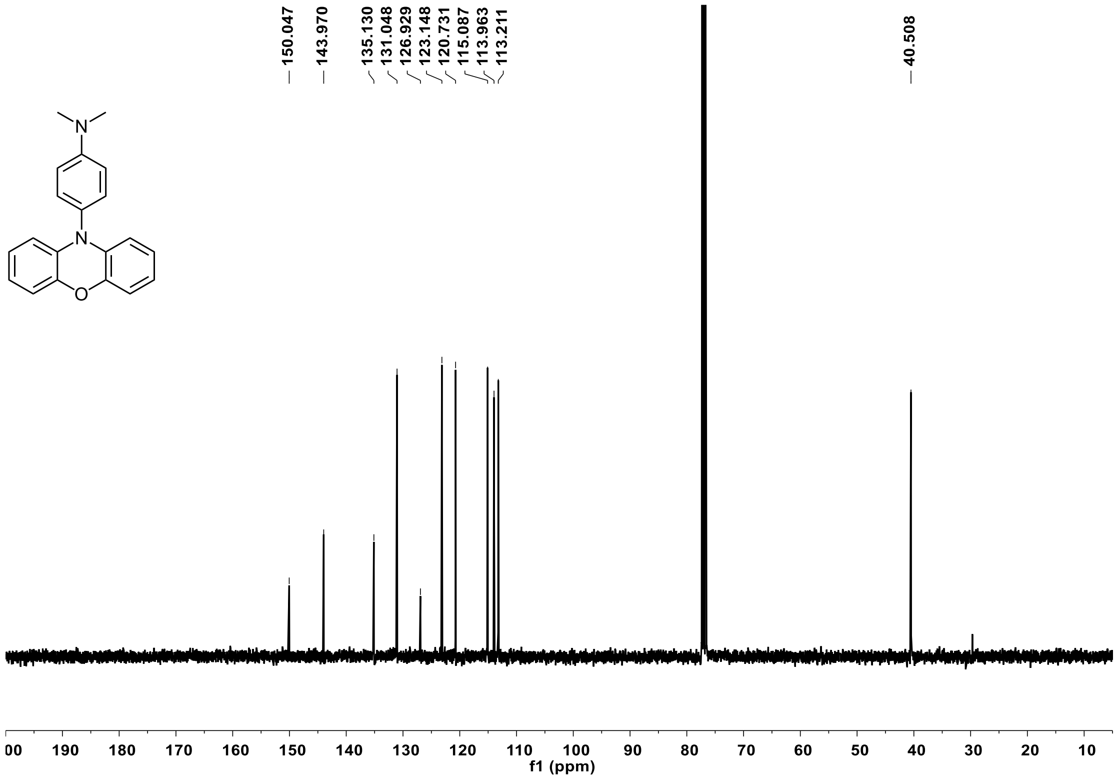

Supplementary Figure 15. ^{13}C NMR (101 MHz, $CDCl_3$) spectrum of 3af

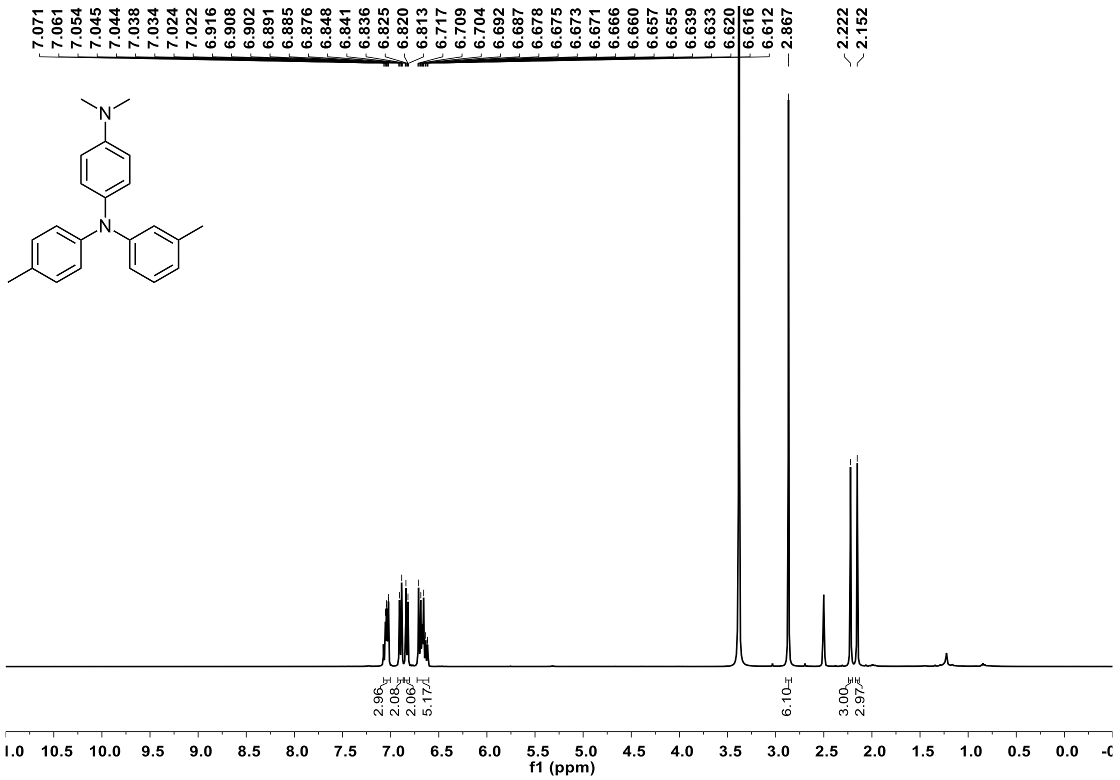

Supplementary Figure 16. ^{1}H NMR (400 MHz, DMSO-d_6) spectrum of 3ag

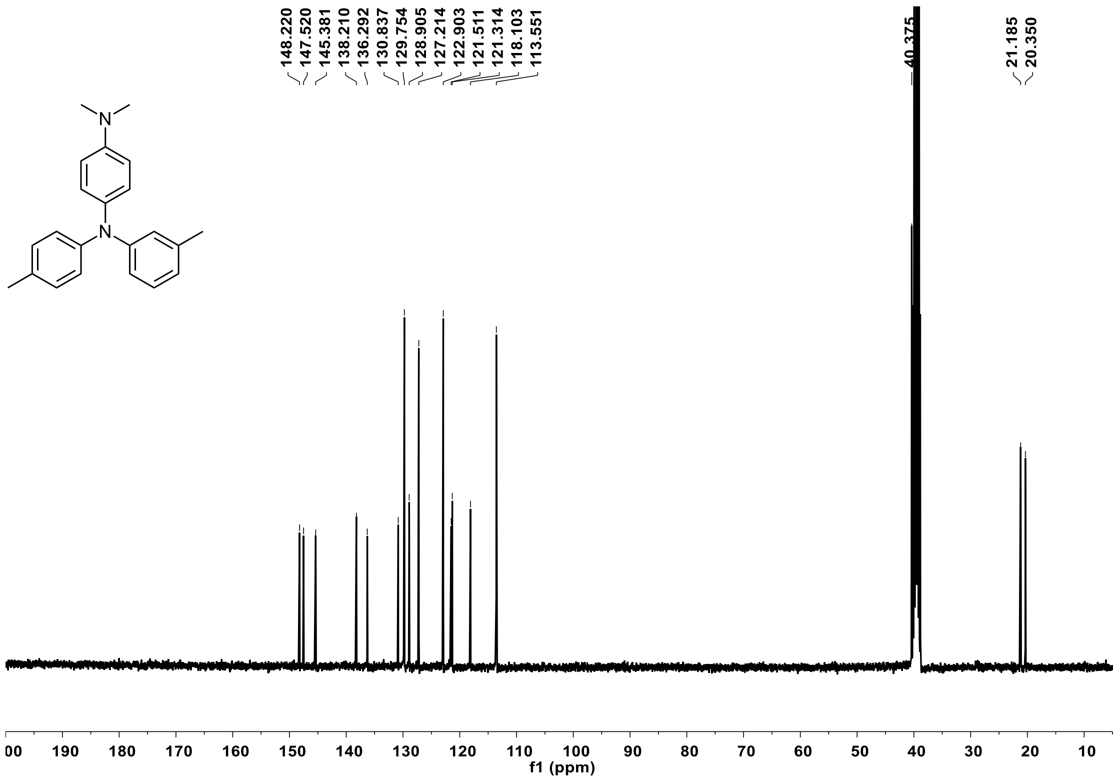

Supplementary Figure 17. ^{13}C NMR (101 MHz, DMSO-d_6) spectrum of 3ag

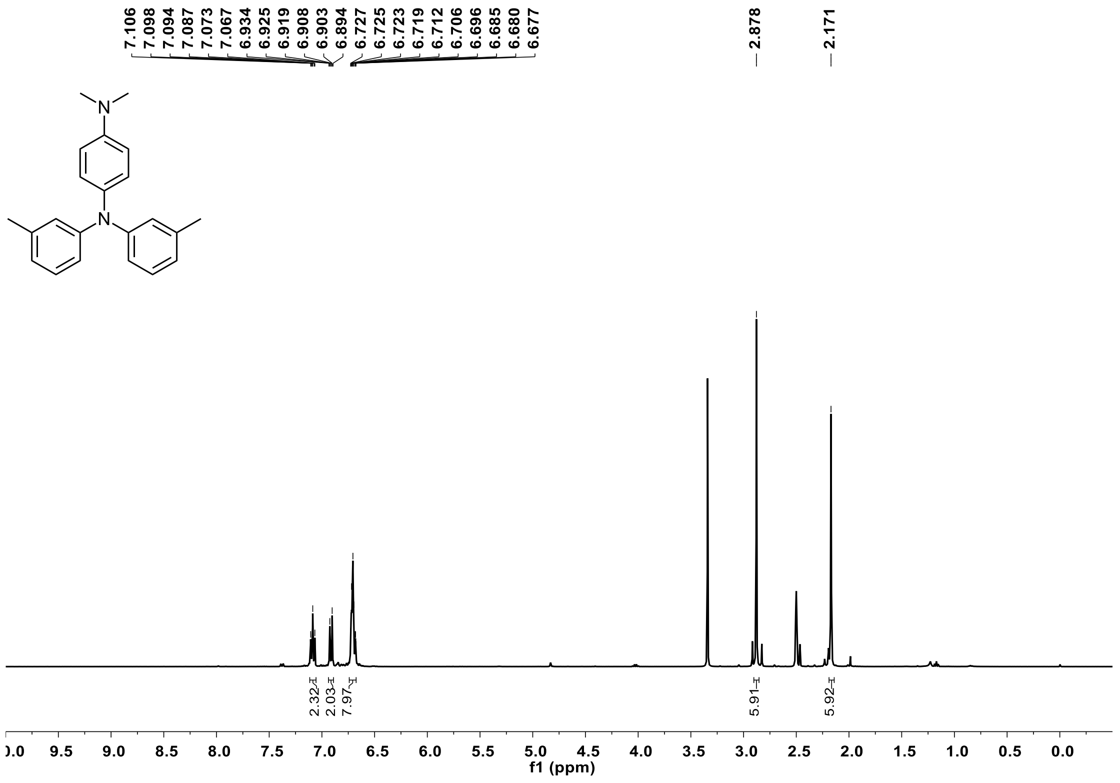

Supplementary Figure 18. ^{1}H **NMR (400 MHz, DMSO-d$_6$) spectrum of 3ah**

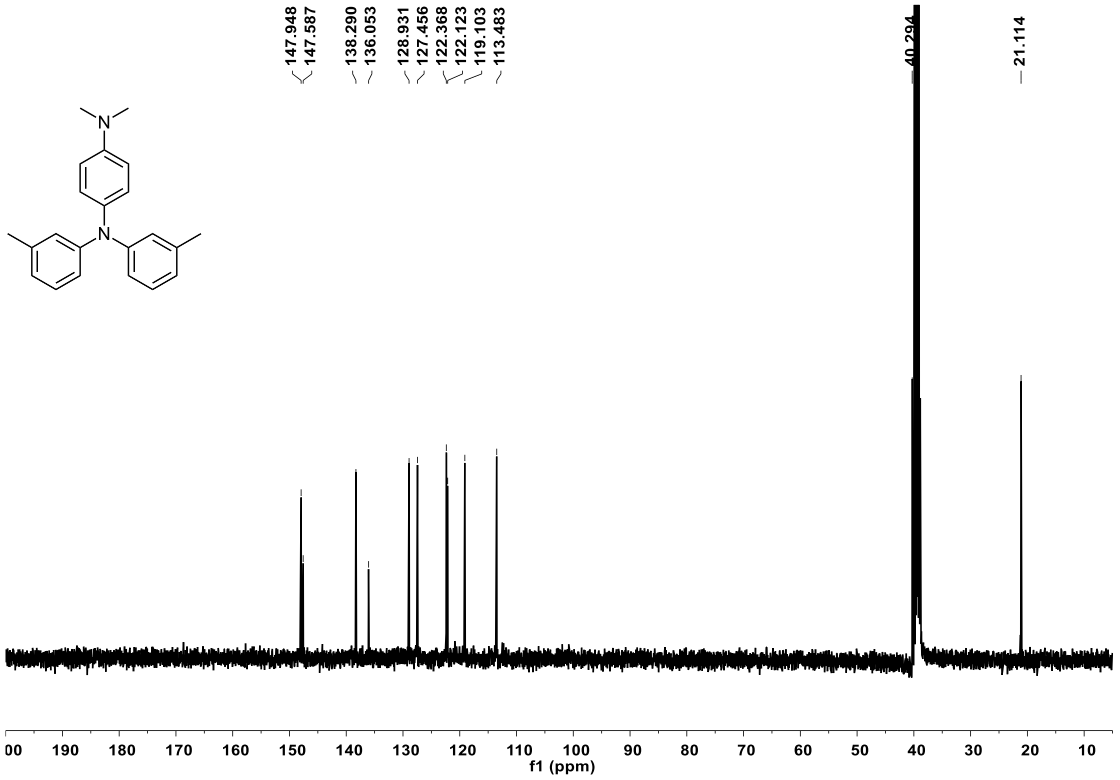

Supplementary Figure 19. ^{13}C **NMR (101 MHz, DMSO-d$_6$) spectrum of 3ah**

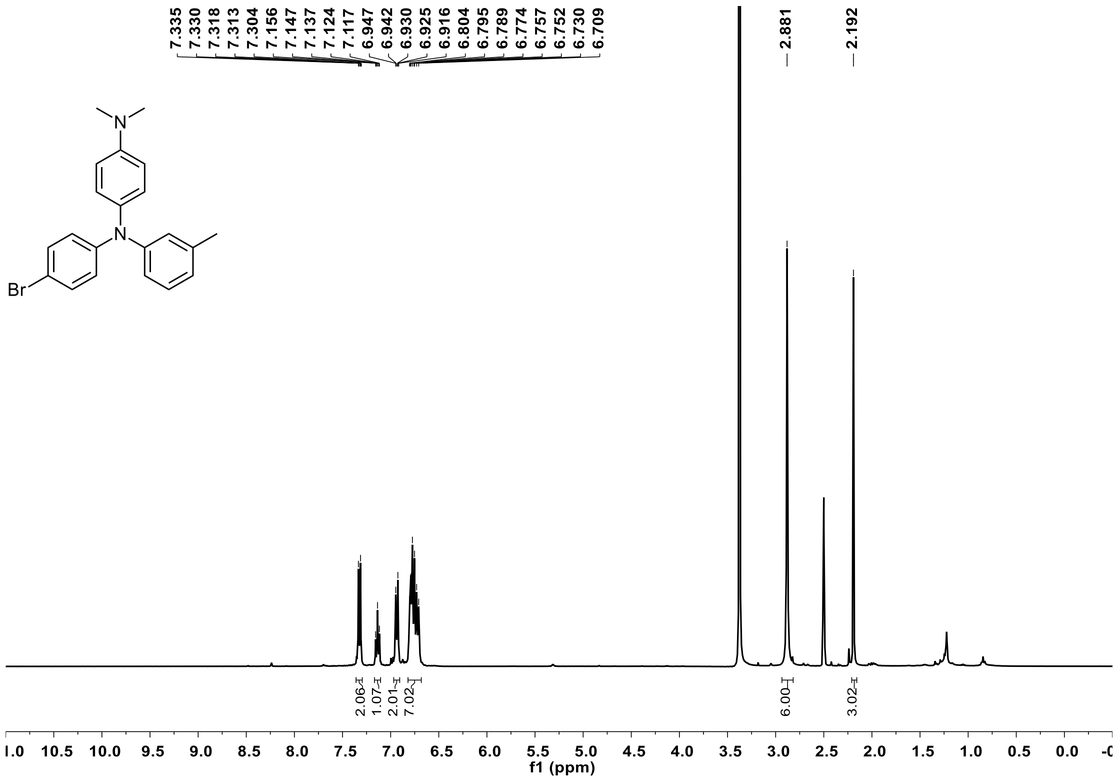

Supplementary Figure 20. ^{1}H NMR (400 MHz, DMSO-d_6) spectrum of 3ai

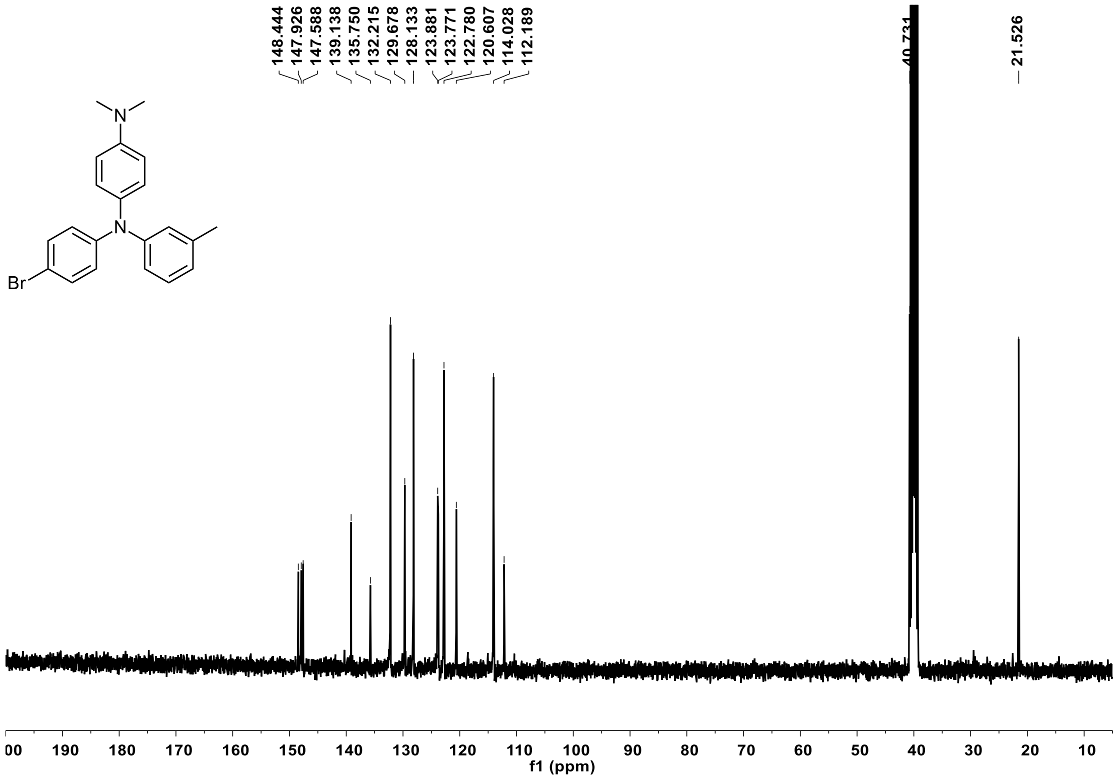

Supplementary Figure 21. ^{13}C NMR (101 MHz, DMSO-d_6) spectrum of 3ai

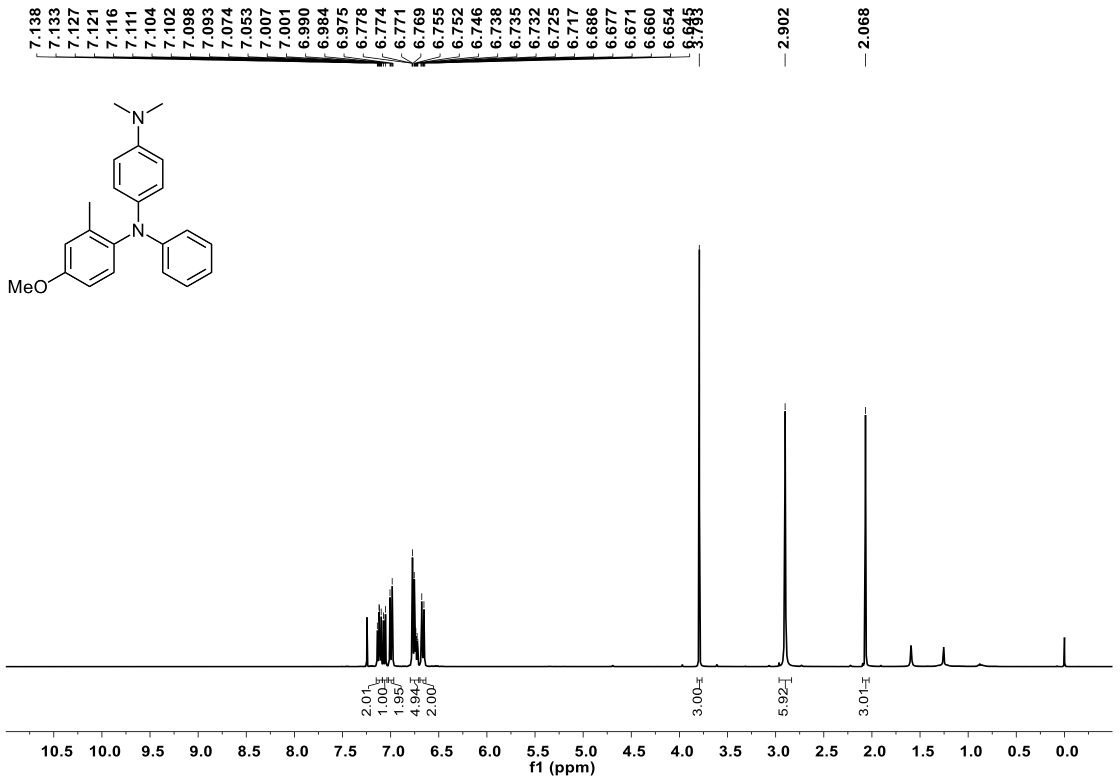

Supplementary Figure 22. ^{1}H NMR (400 MHz, $CDCl_3$) spectrum of 3aj

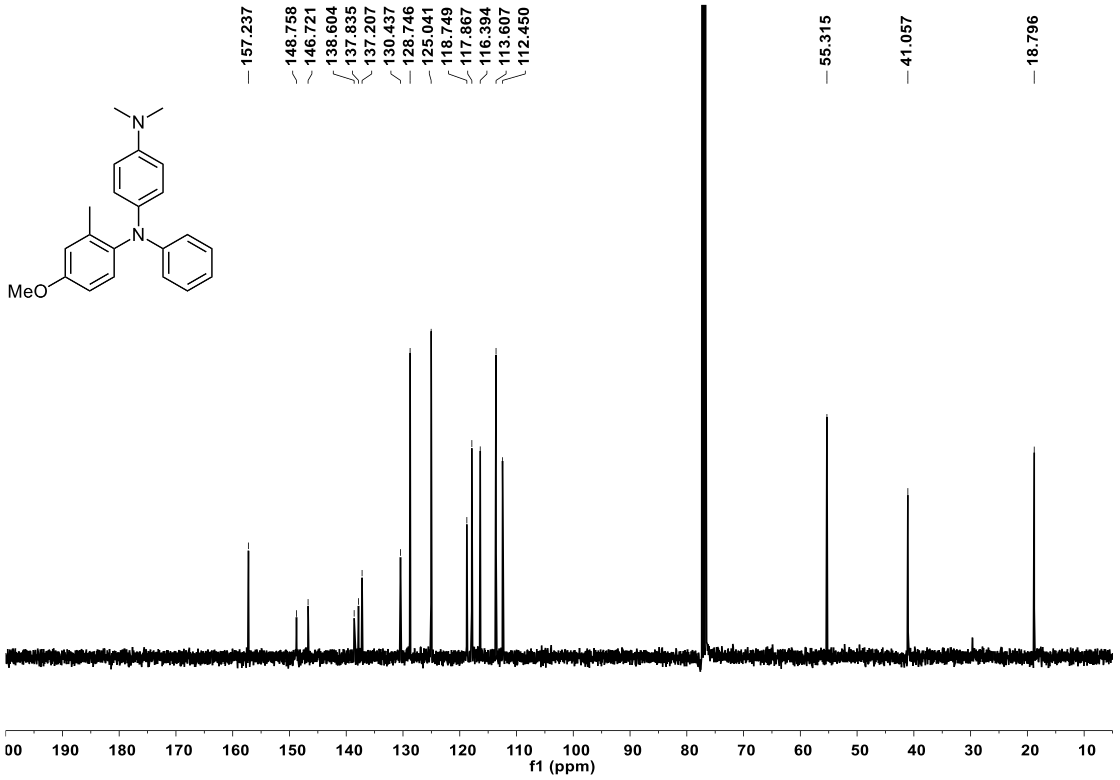

Supplementary Figure 23. ^{13}C NMR (101 MHz, $CDCl_3$) spectrum of 3aj

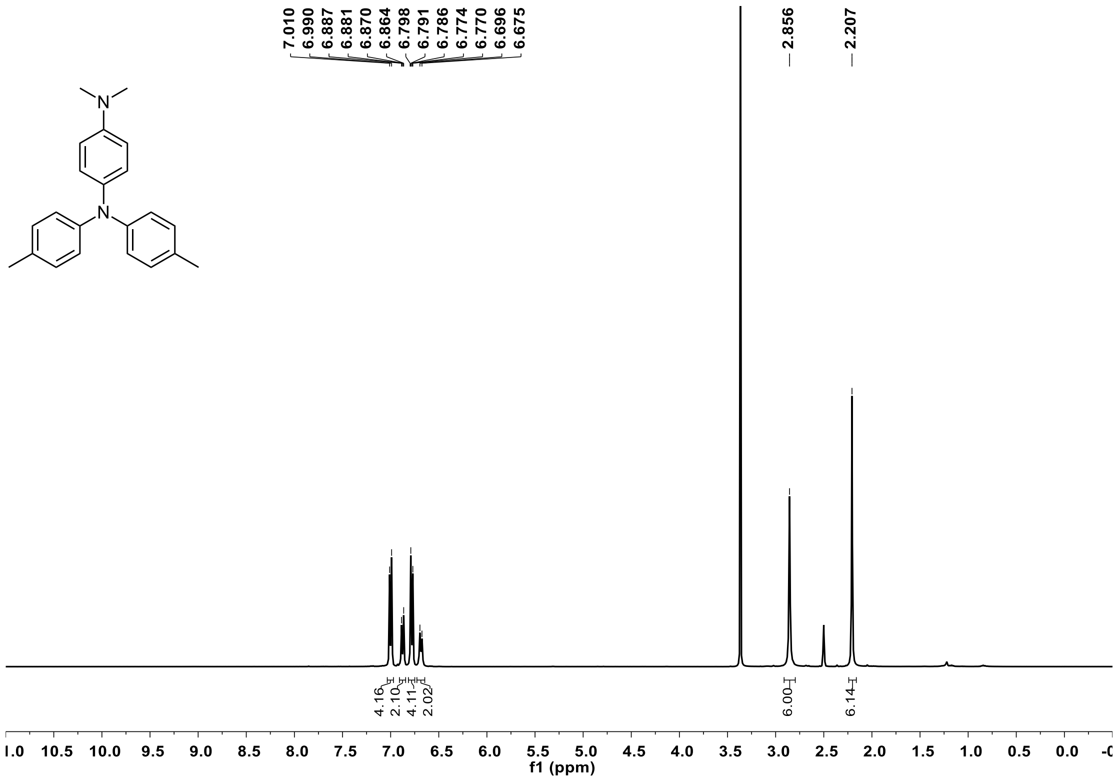

Supplementary Figure 24. ^{1}H NMR (400 MHz, DMSO-d_6) spectrum of 3ak

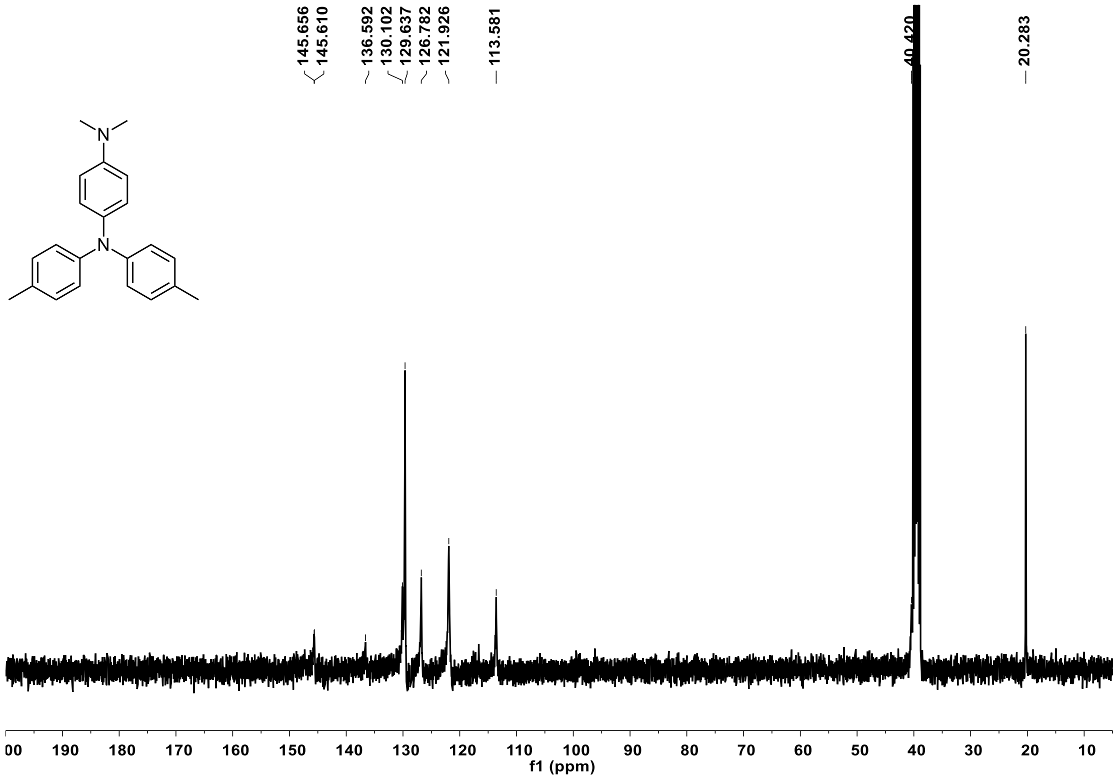

Supplementary Figure 25. ^{13}C NMR (101 MHz, DMSO-d_6) spectrum of 3ak

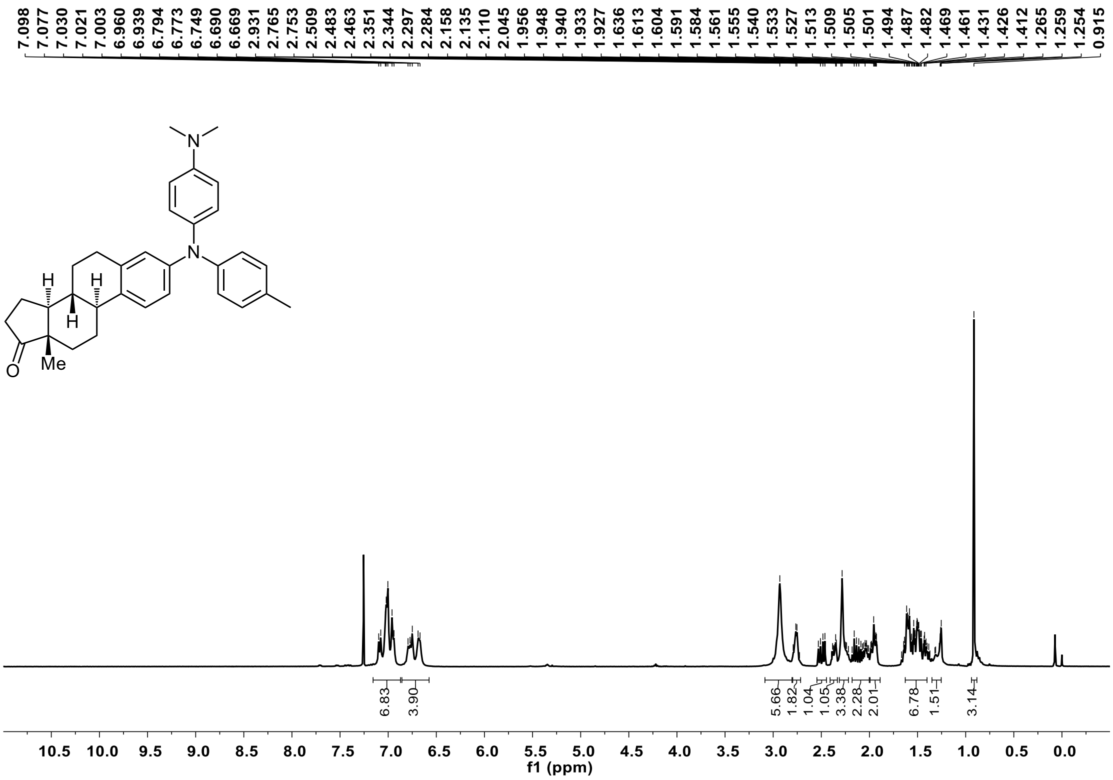

Supplementary Figure 26. ^{1}H NMR (400 MHz, $CDCl_3$) spectrum of 3al

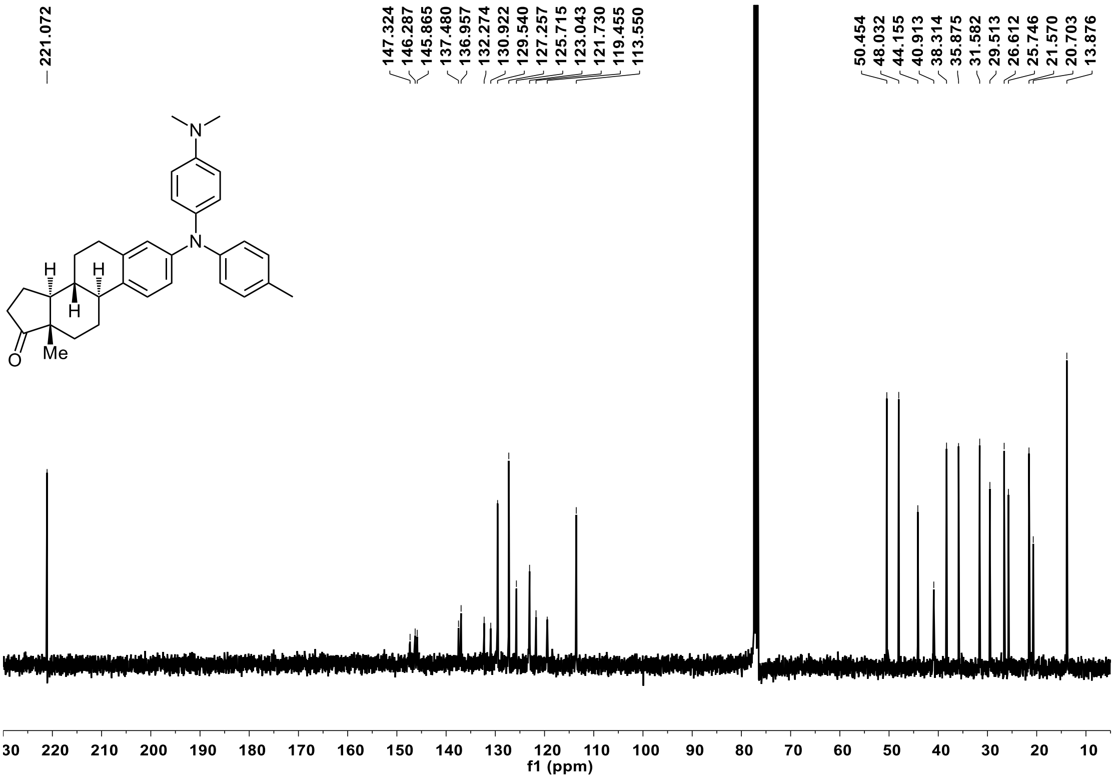

Supplementary Figure 27. ^{13}C NMR (101 MHz, $CDCl_3$) spectrum of 3al

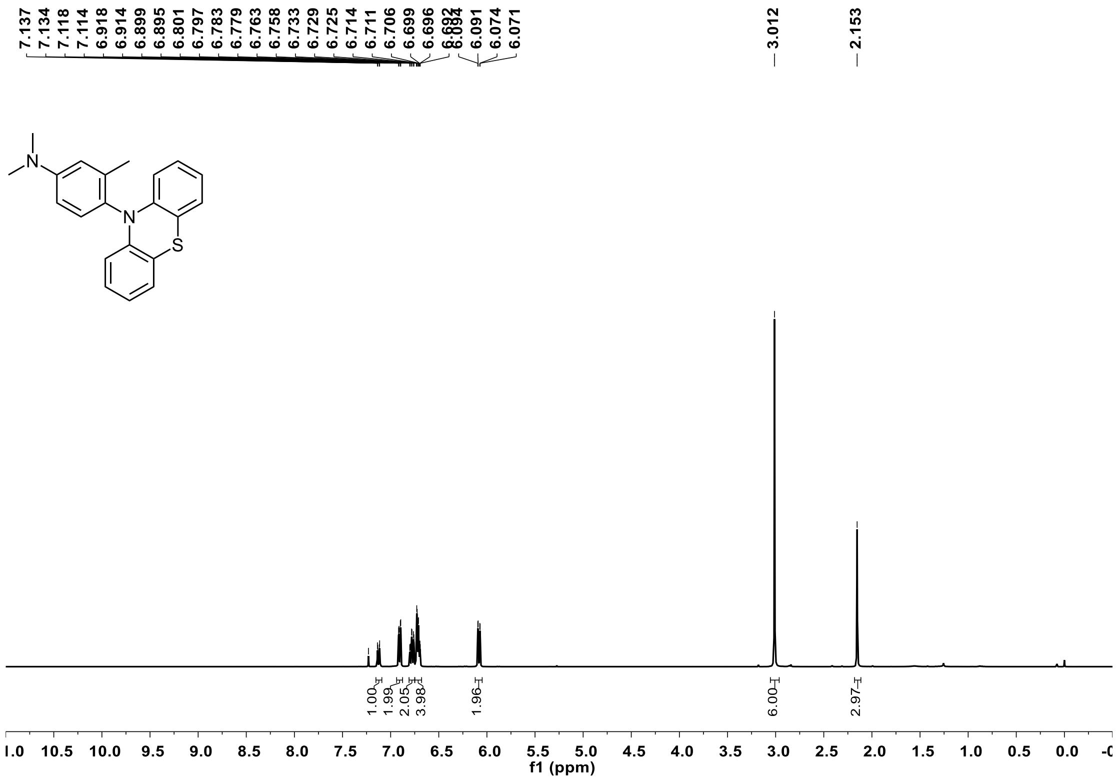

Supplementary Figure 28. ^{1}H NMR (400 MHz, $CDCl_3$) spectrum of 3ba

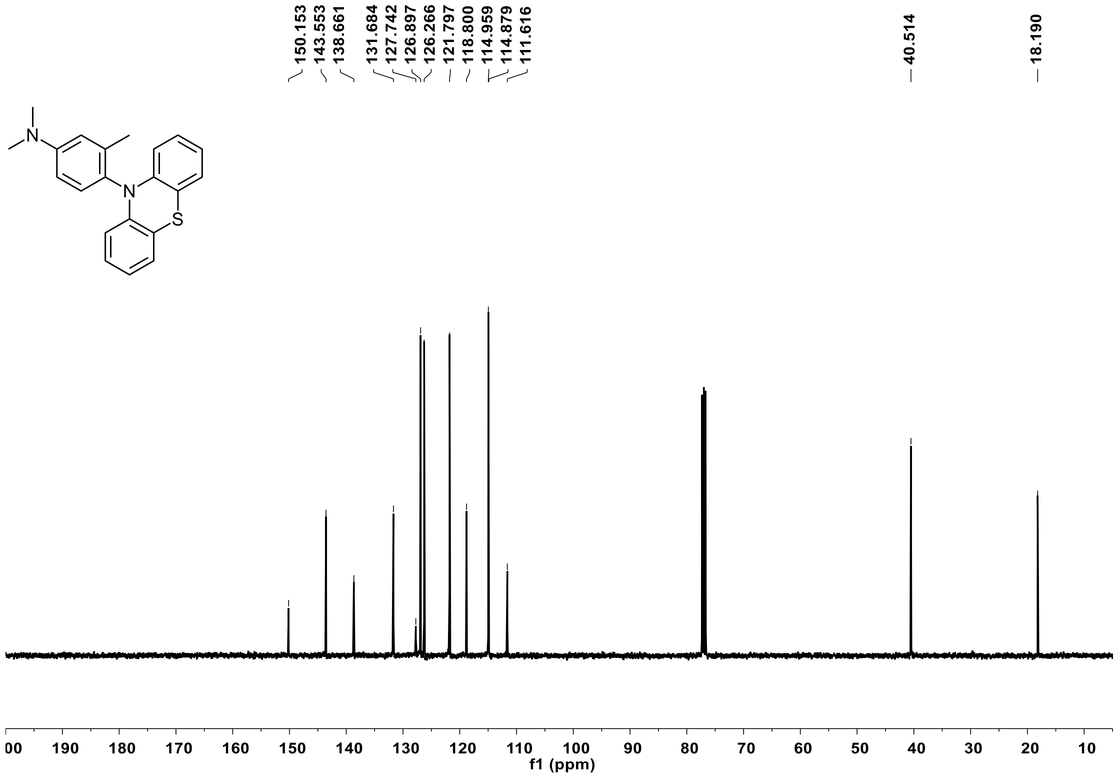

Supplementary Figure 29. ^{13}C NMR (101 MHz, $CDCl_3$) spectrum of 3ba

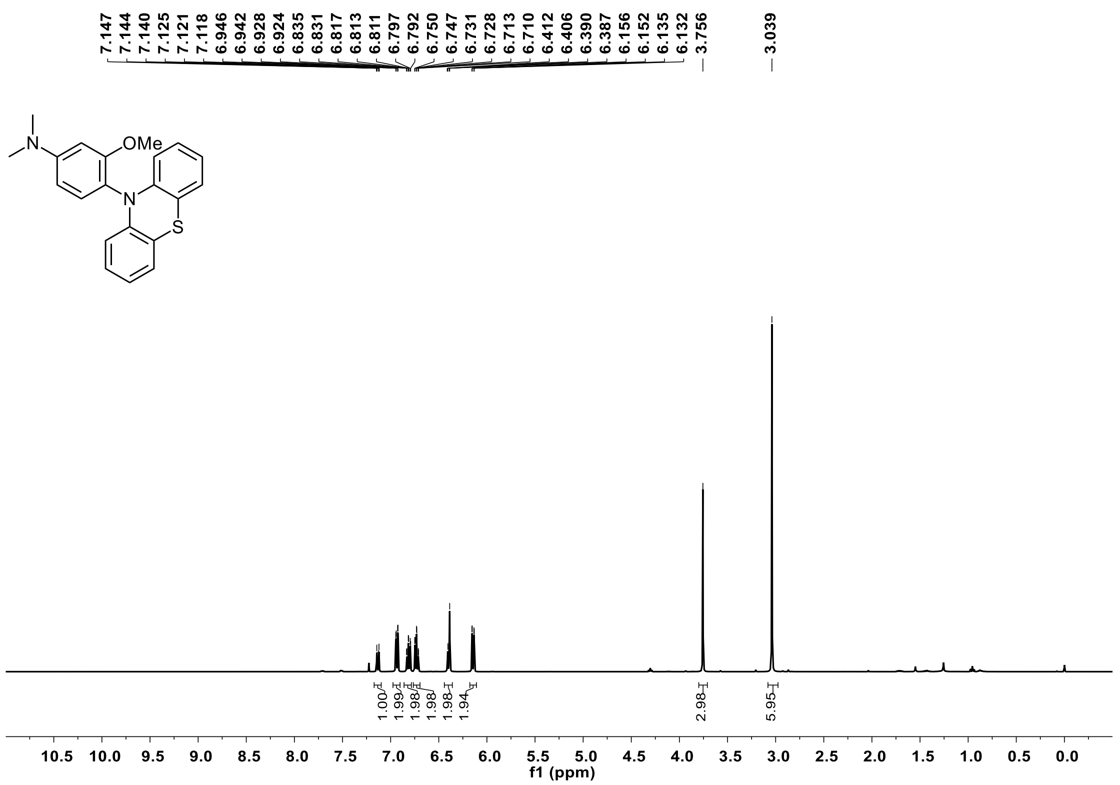

Supplementary Figure 30. ^{1}H NMR (400 MHz, $CDCl_3$) spectrum of 3ca

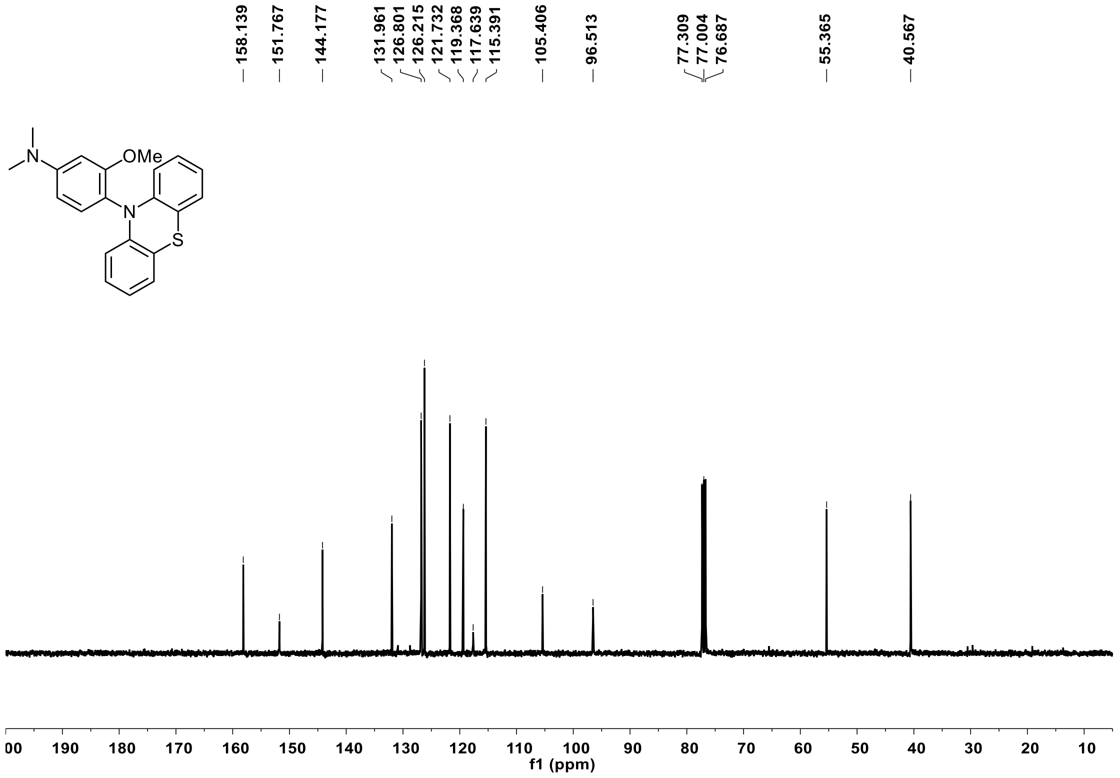

Supplementary Figure 31. ^{13}C NMR (101 MHz, $CDCl_3$) spectrum of 3ca

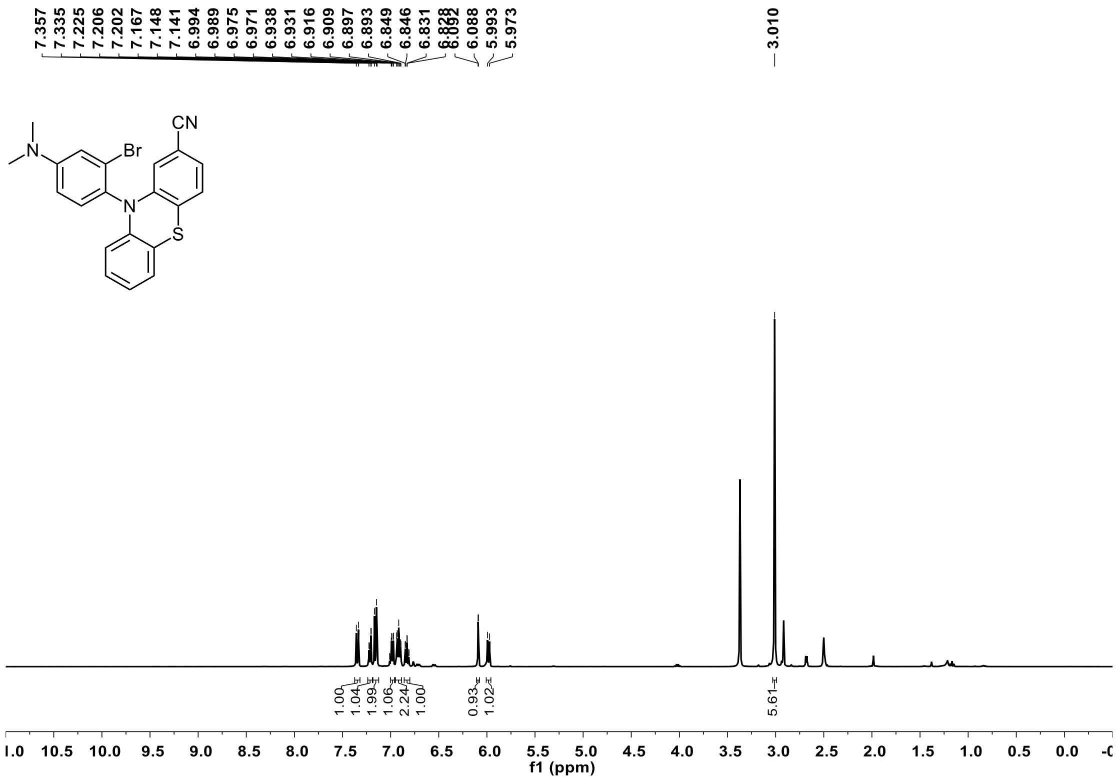

Supplementary Figure 32. ^{1}**H NMR (400 MHz, DMSO-d**$_6$**) spectrum of 3dd**

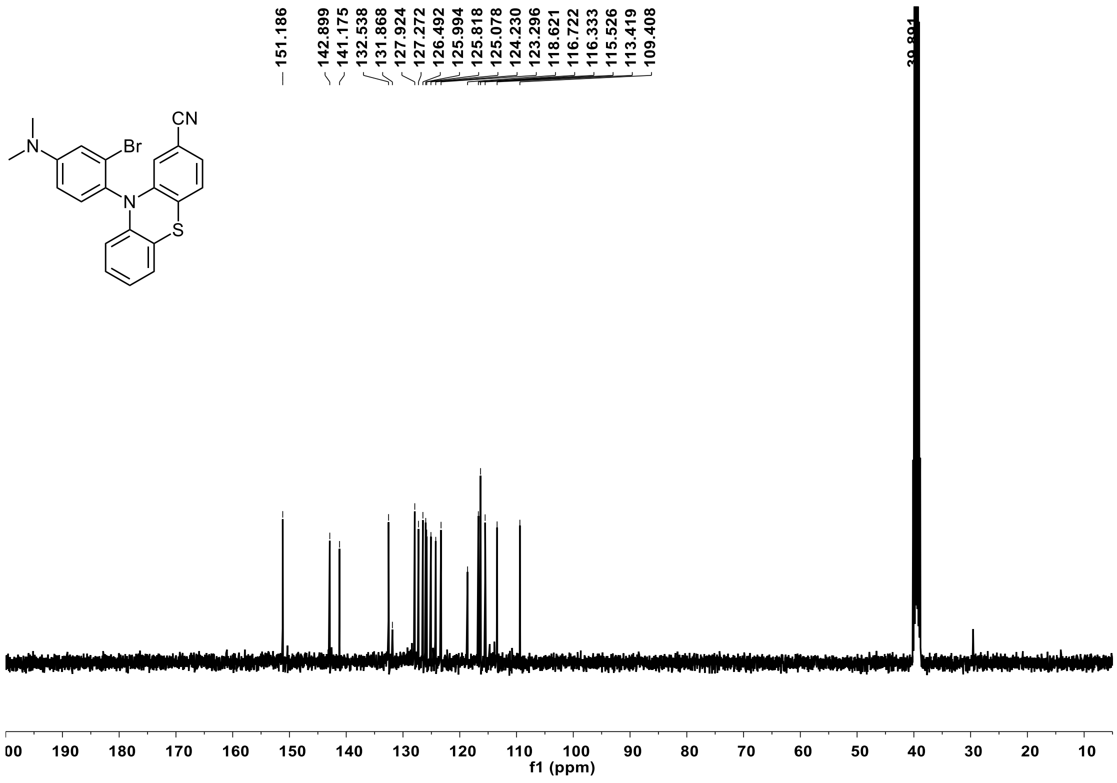

Supplementary Figure 33. ^{13}**C NMR (101 MHz, DMSO-d**$_6$**) spectrum of 3dd**

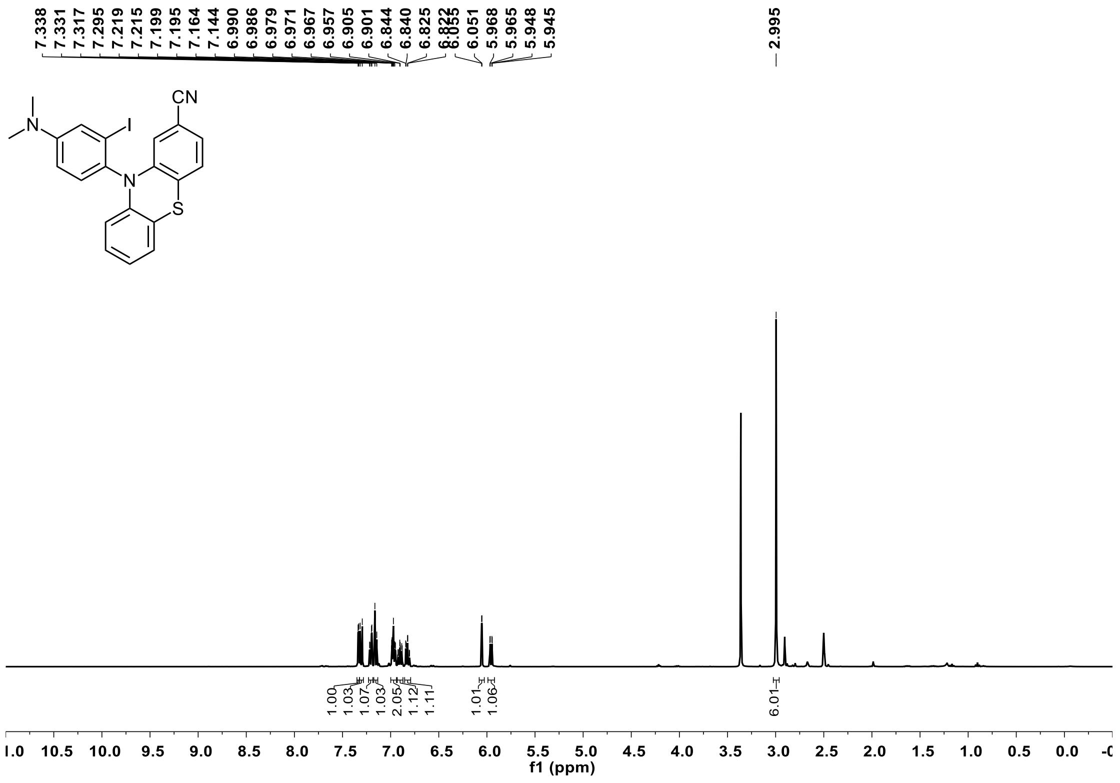

Supplementary Figure 34. ^{1}H **NMR (400 MHz, DMSO-d$_6$) spectrum of 3ed**

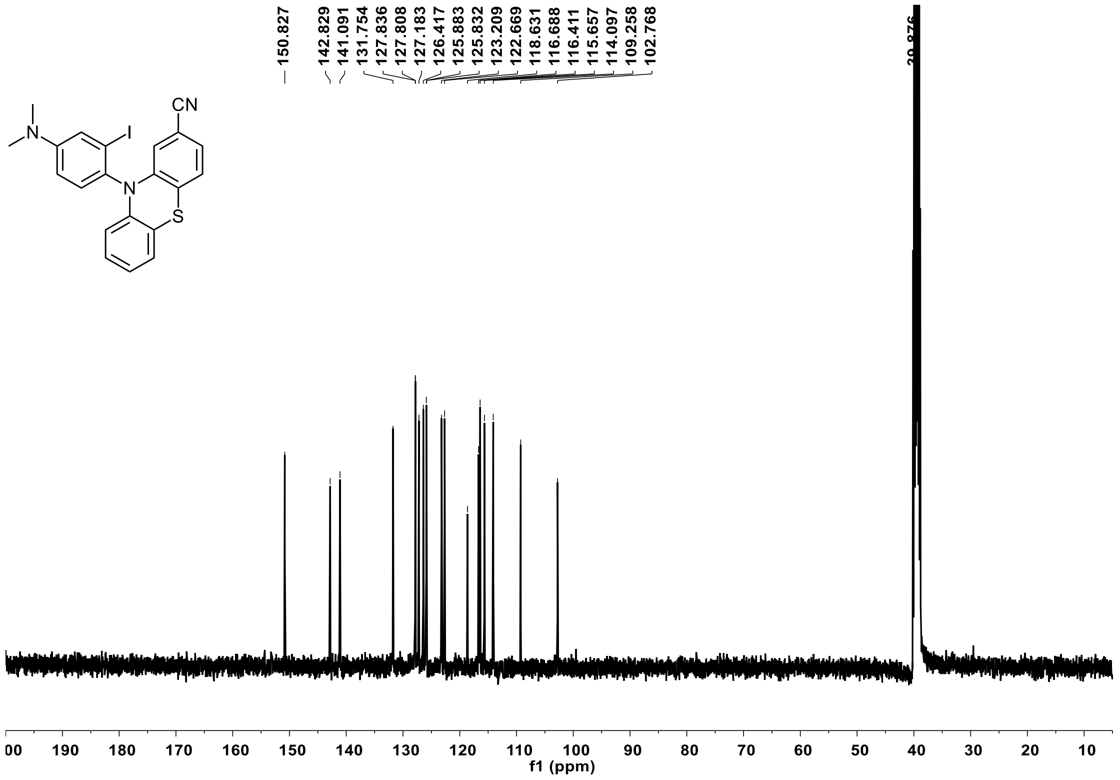

Supplementary Figure 35. ^{13}C **NMR (101 MHz, DMSO-d$_6$) spectrum of 3ed**

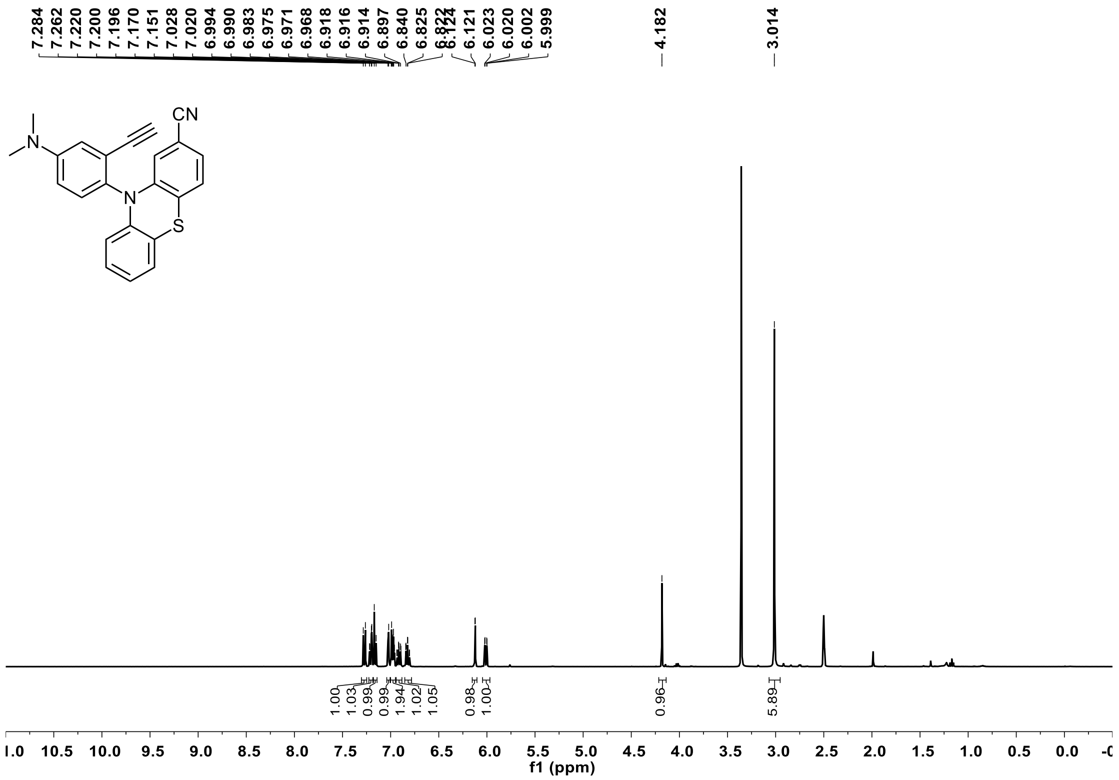

Supplementary Figure 36. ^{1}H NMR (400 MHz, DMSO-d_6) spectrum of 3fd

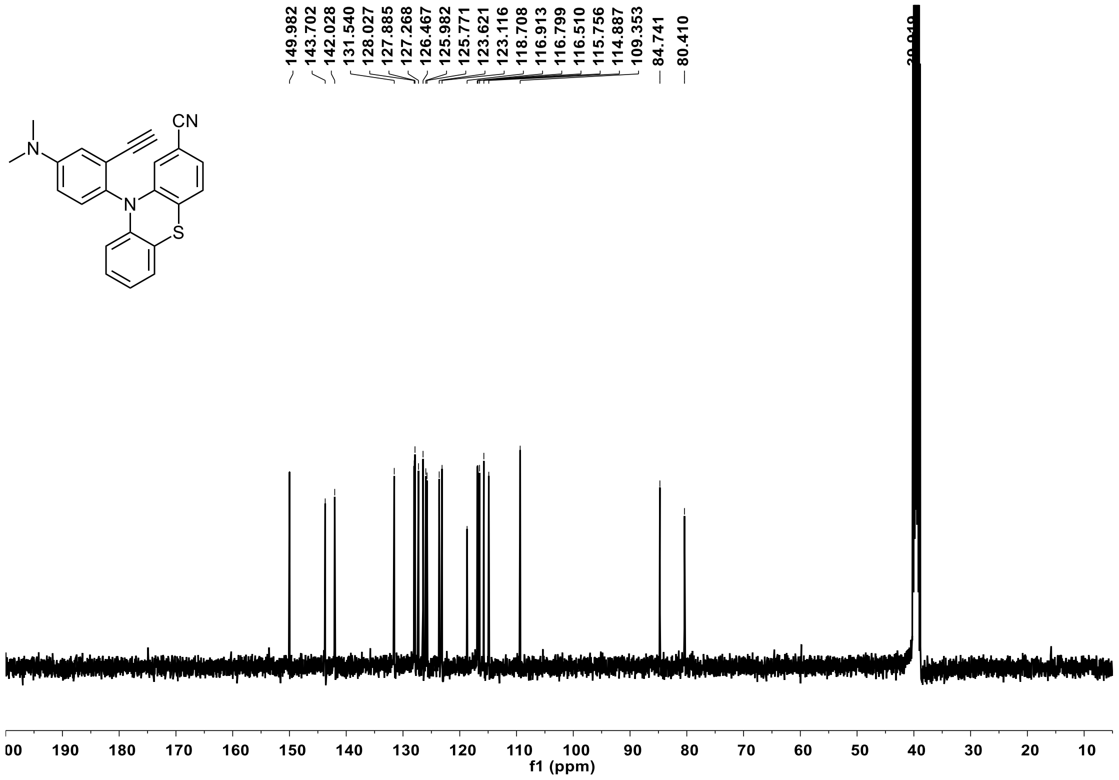

Supplementary Figure 37. ^{13}C NMR (101 MHz, DMSO-d_6) spectrum of 3fd

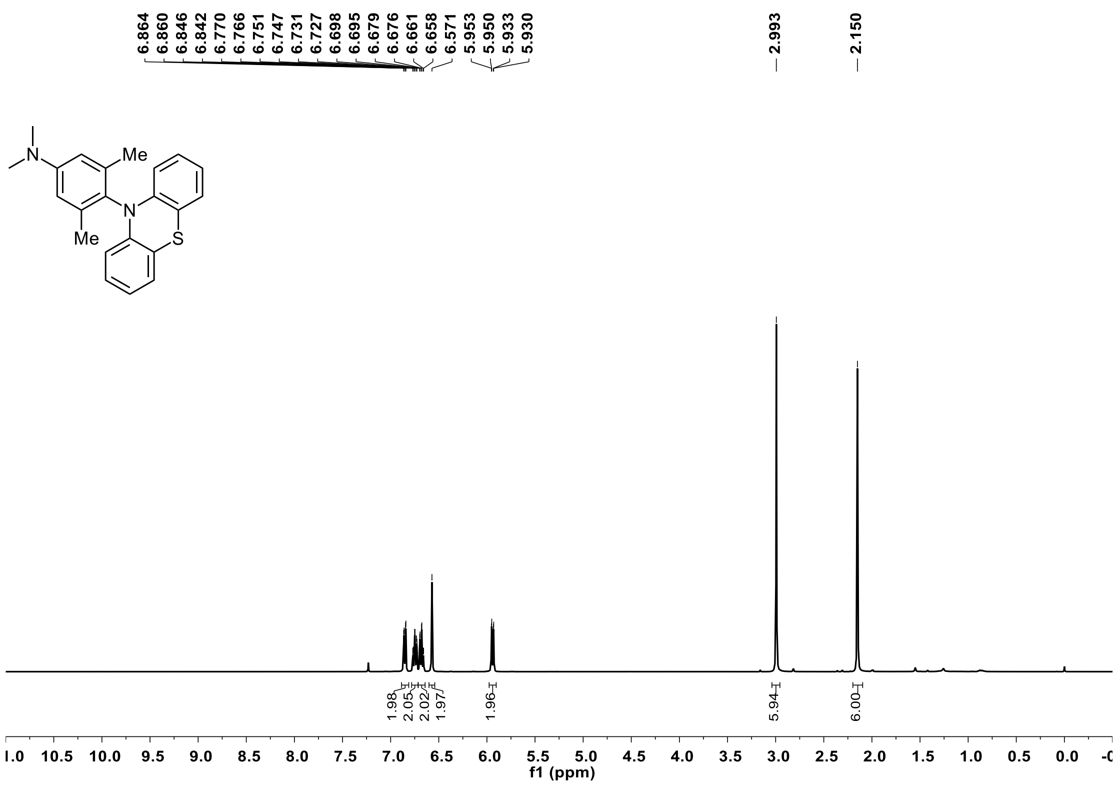

Supplementary Figure 38. ^{1}H NMR (400 MHz, $CDCl_3$) spectrum of 3ga

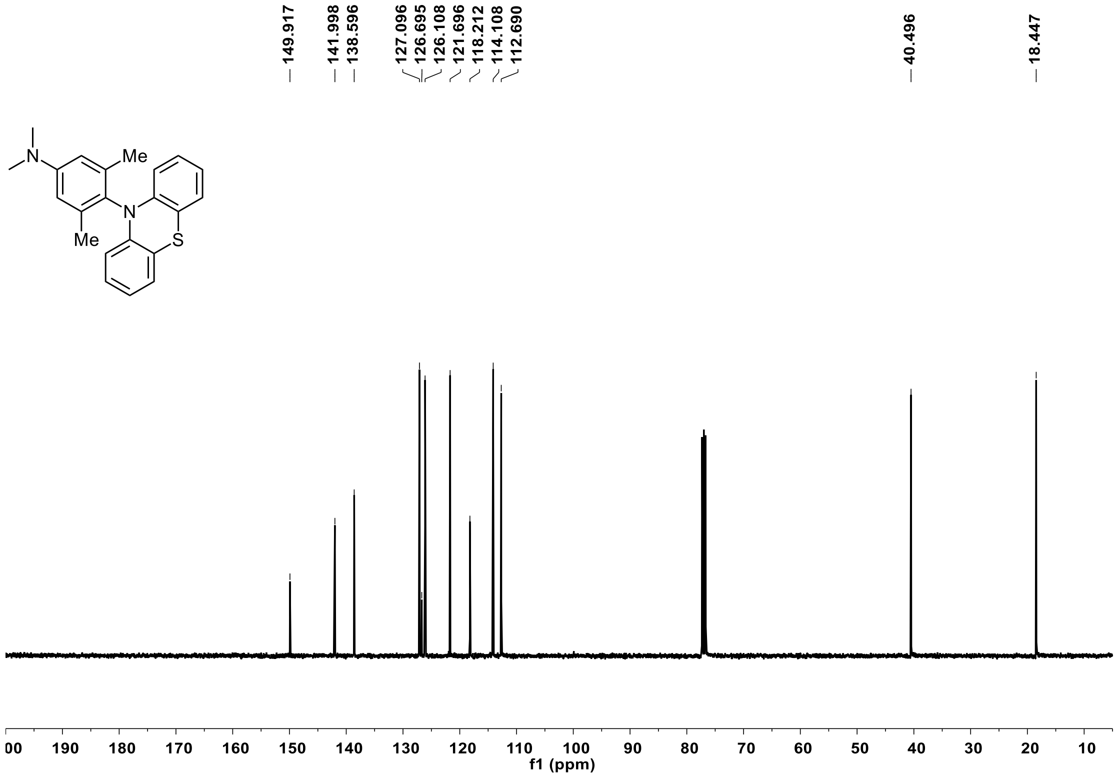

Supplementary Figure 39. ^{13}C NMR (101 MHz, $CDCl_3$) spectrum of 3ga

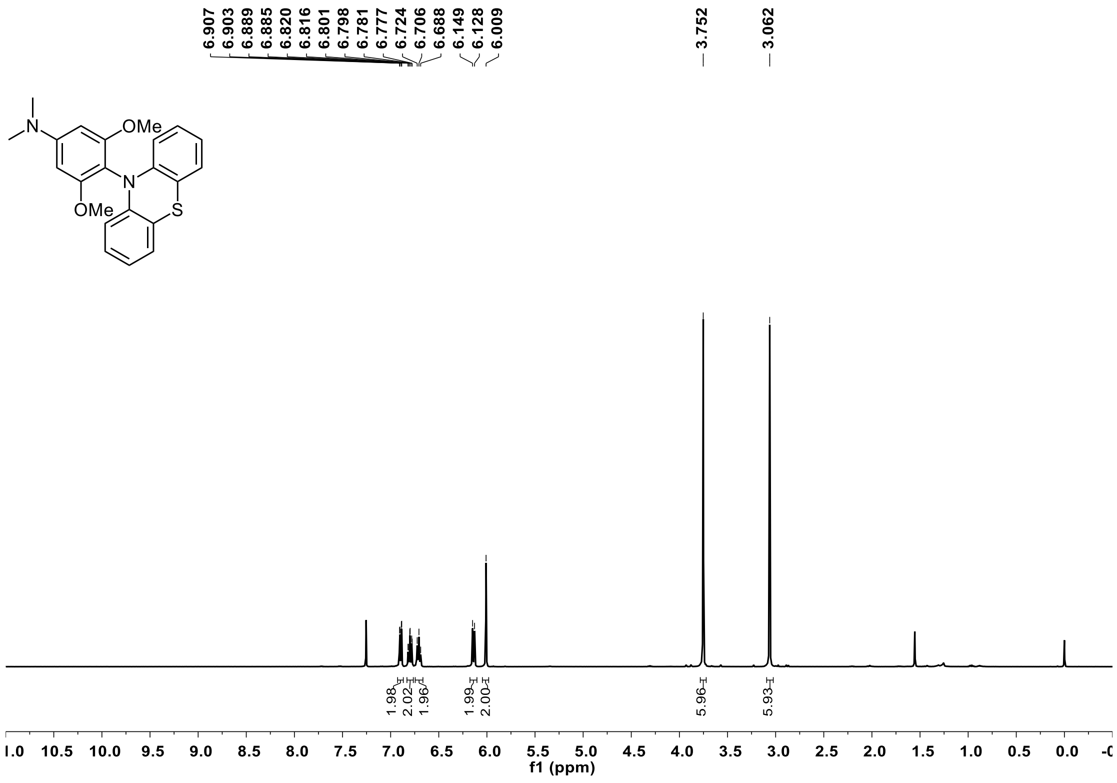

Supplementary Figure 40. ^{1}H NMR (400 MHz, $CDCl_3$) spectrum of 3ha

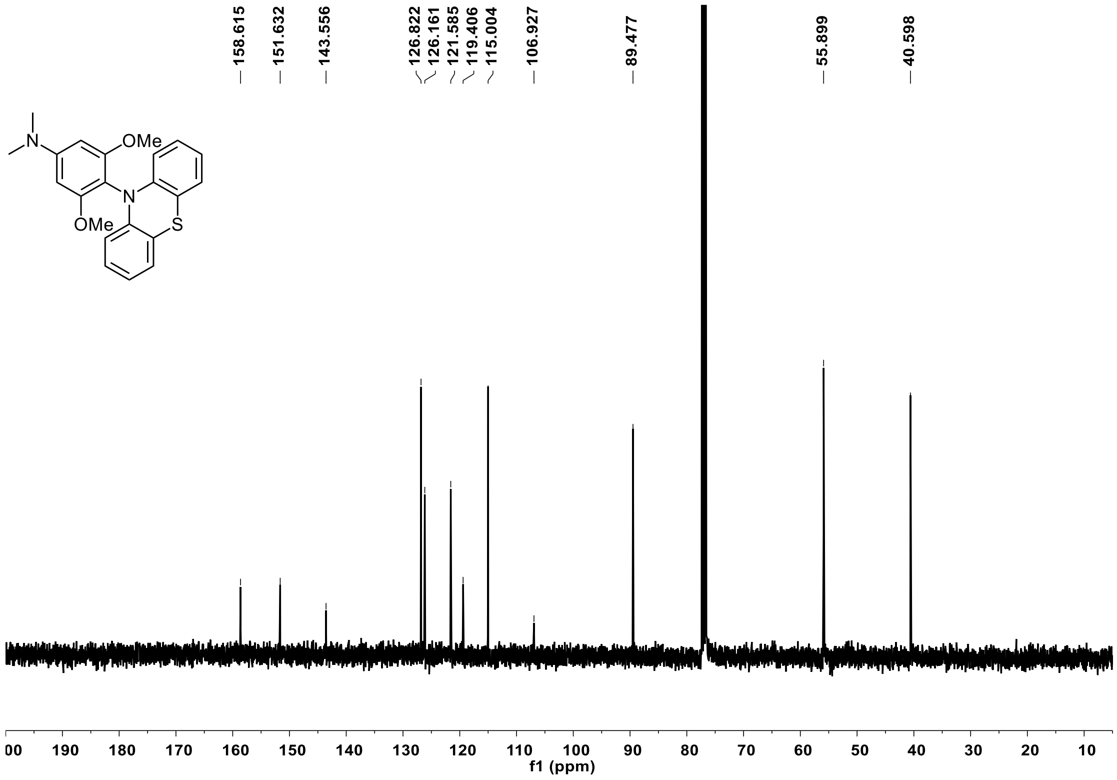

Supplementary Figure 41. ^{13}C NMR (101 MHz, $CDCl_3$) spectrum of 3ha

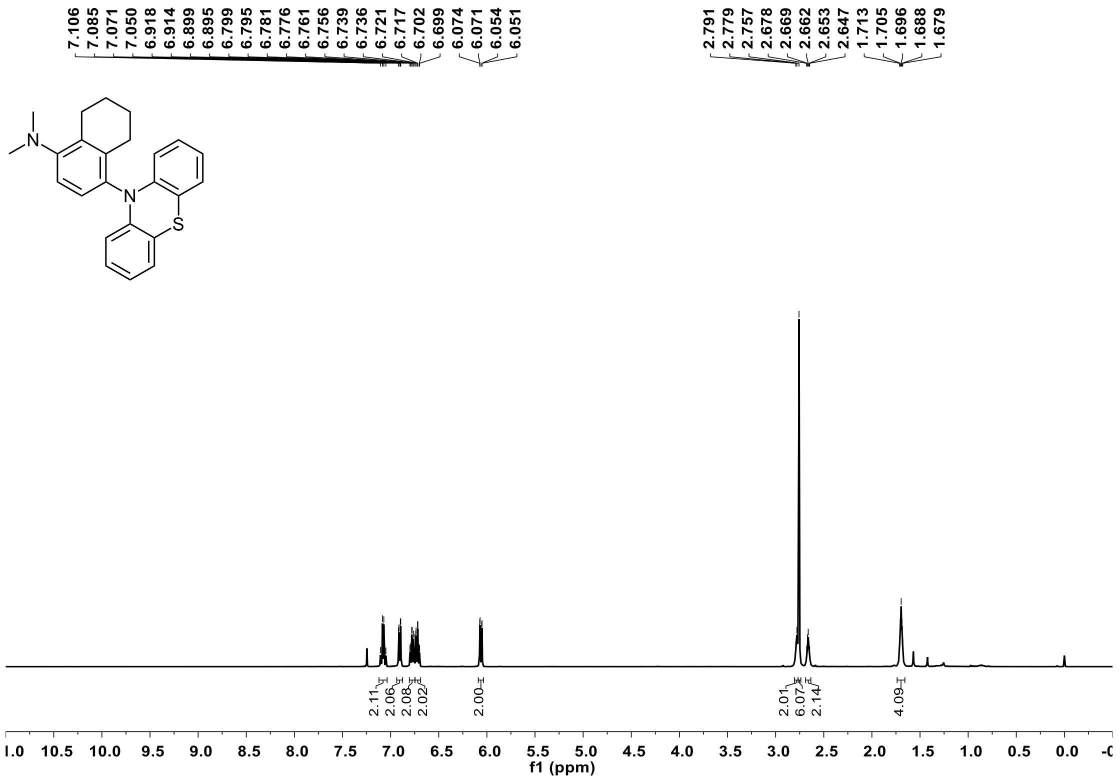

Supplementary Figure 42. ^{1}H NMR (400 MHz, $CDCl_3$) spectrum of 3ia

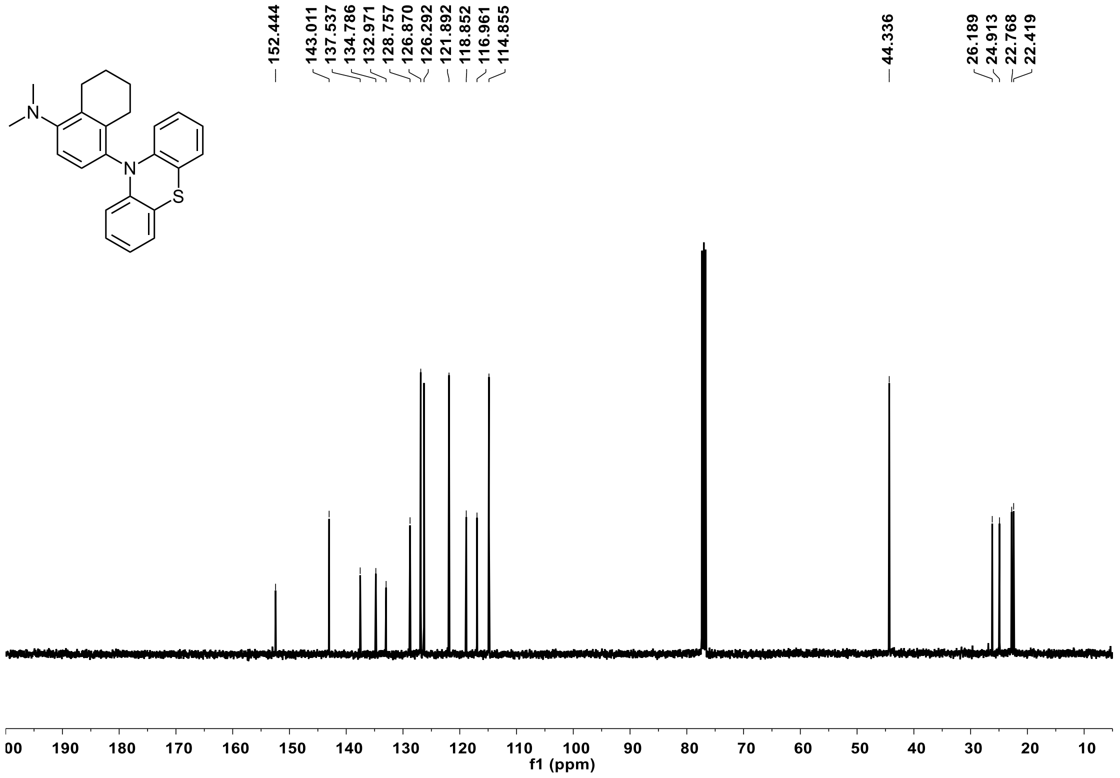

Supplementary Figure 43. ^{13}C NMR (101 MHz, $CDCl_3$) spectrum of 3ia

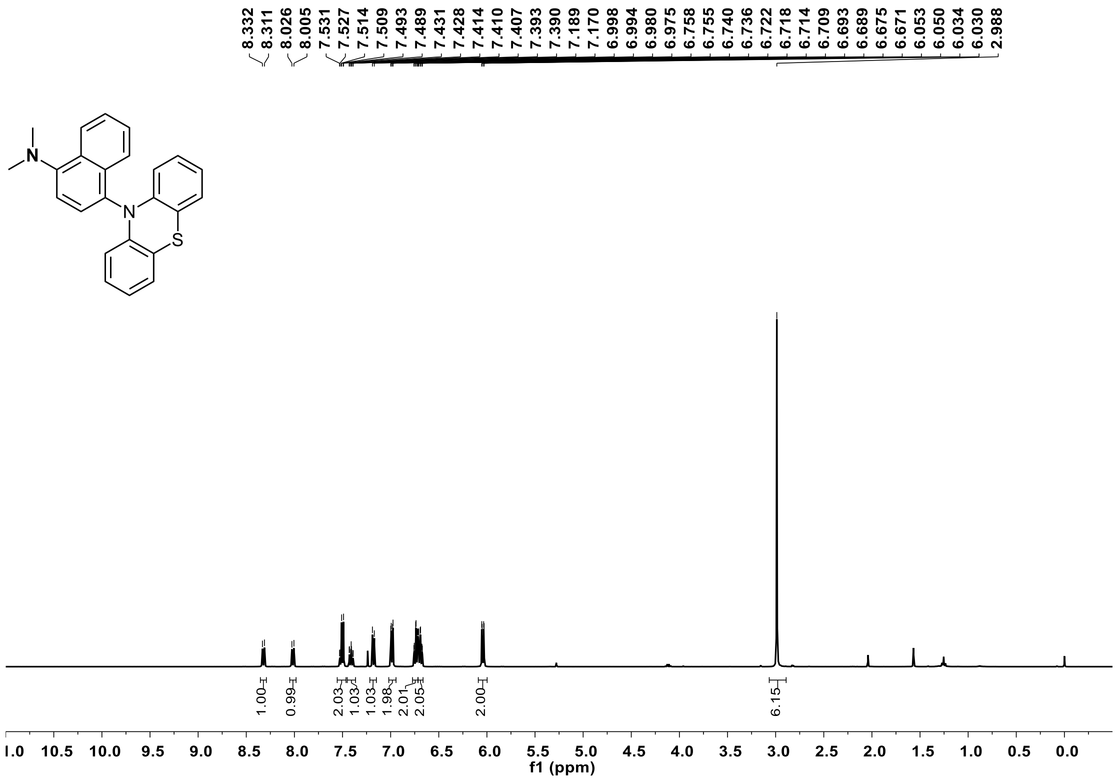

Supplementary Figure 44. ^{1}H NMR (400 MHz, $CDCl_3$) spectrum of 3ja

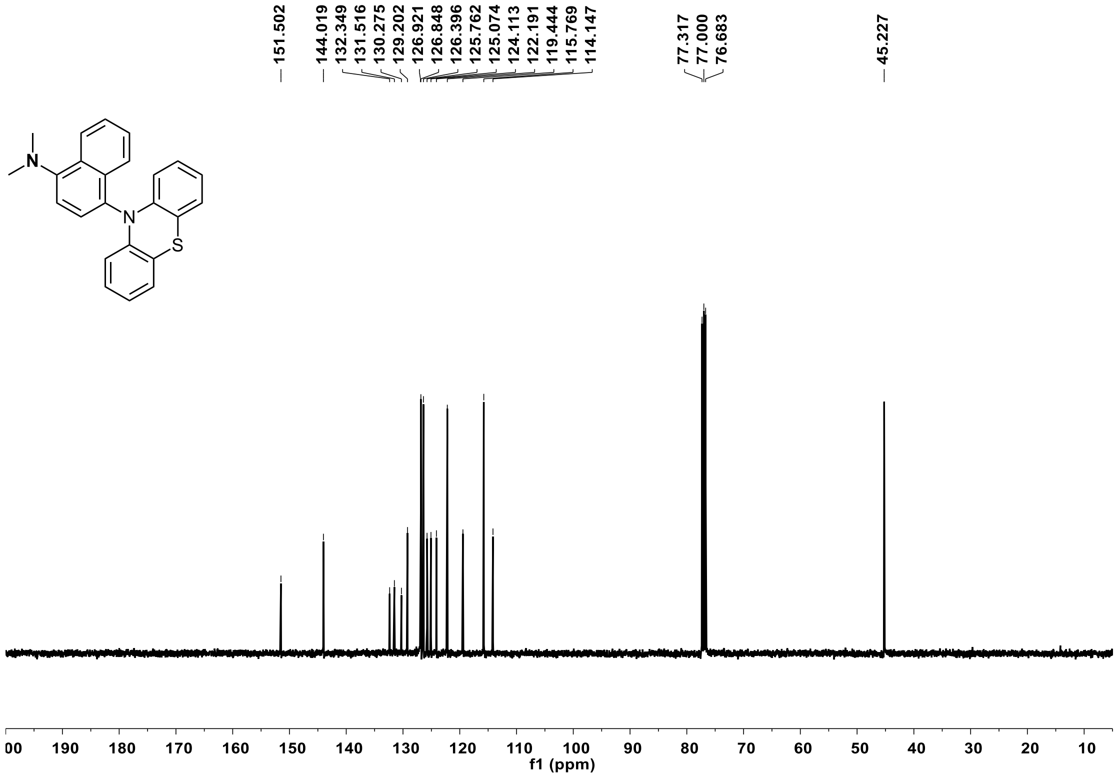

Supplementary Figure 45. ^{13}C NMR (101 MHz, $CDCl_3$) spectrum of 3ja

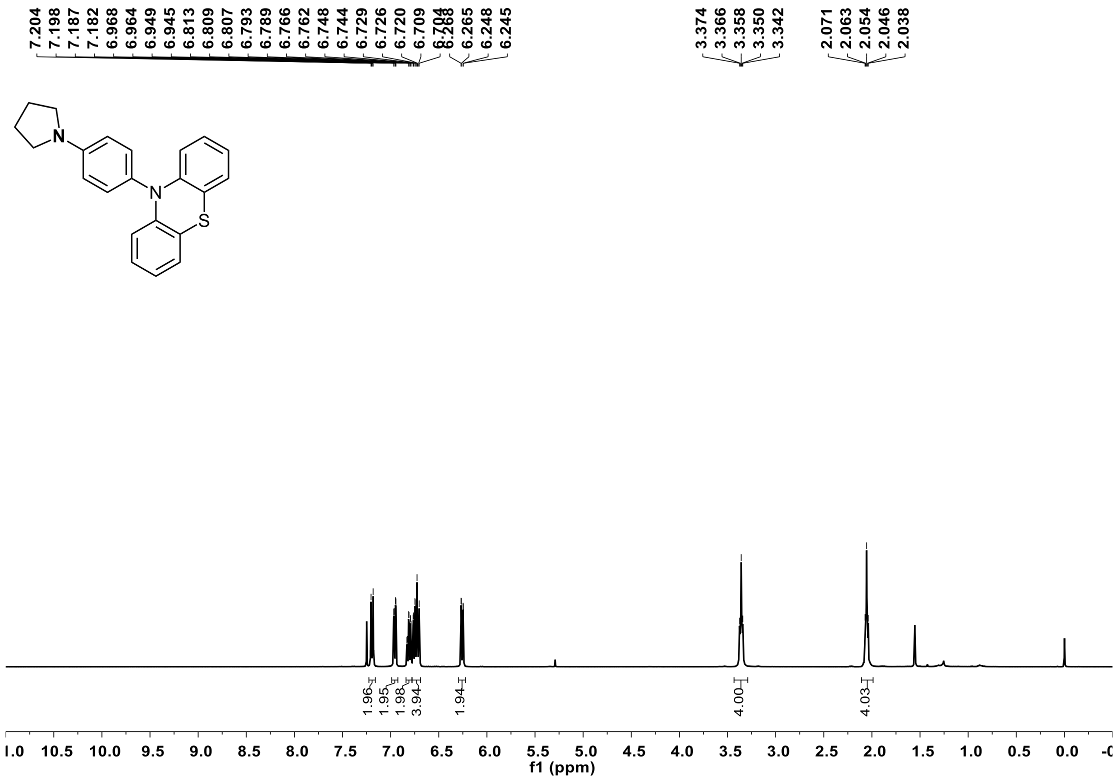

Supplementary Figure 46. ^{1}H NMR (400 MHz, $CDCl_3$) spectrum of 3ka

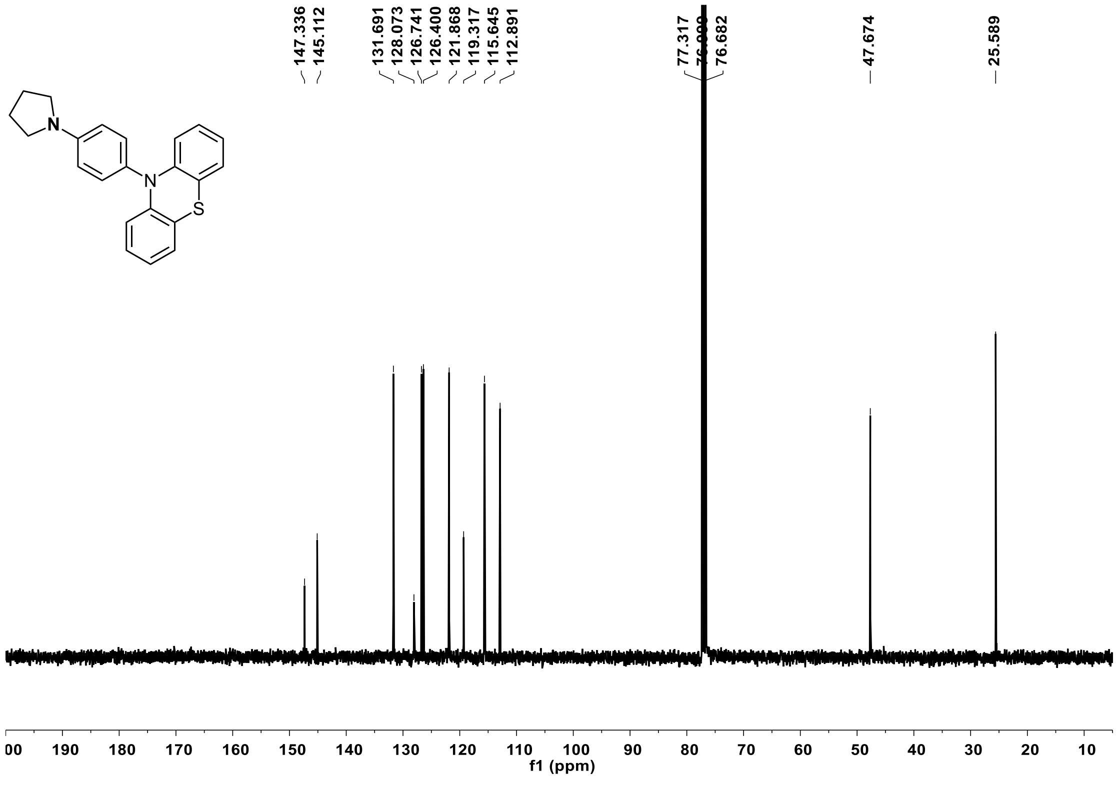

Supplementary Figure 47. ^{13}C NMR (101 MHz, $CDCl_3$) spectrum of 3ka

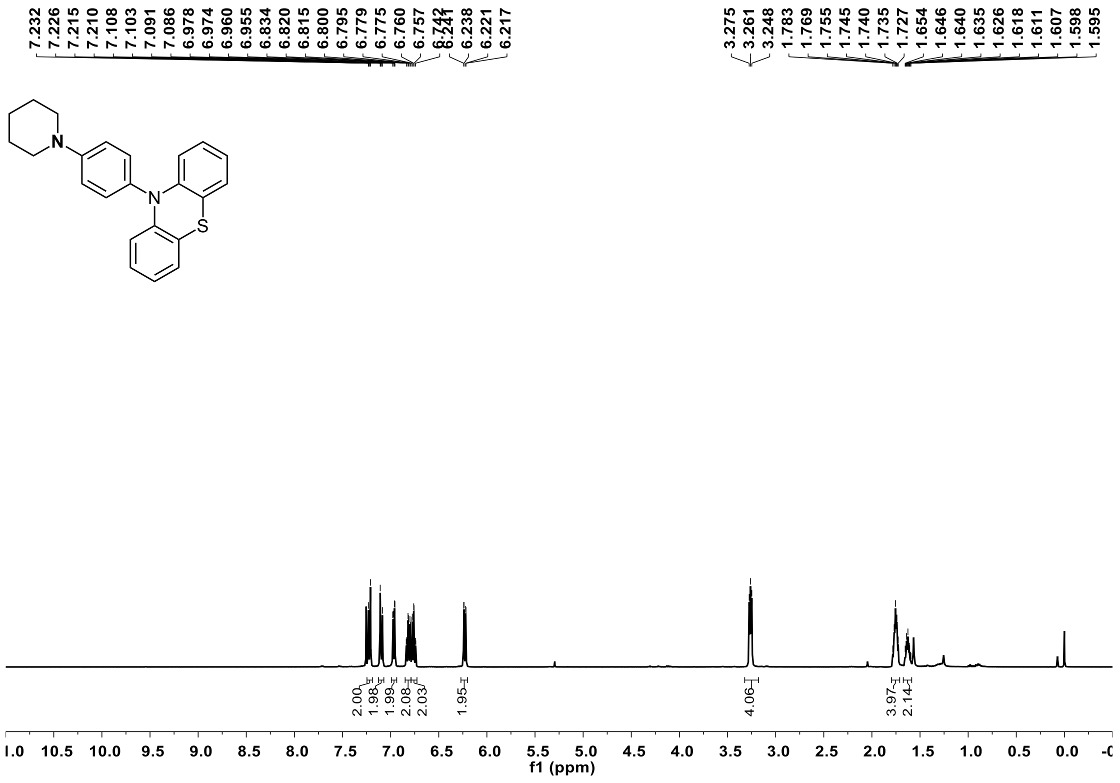

Supplementary Figure 48. ^{1}H NMR (400 MHz, $CDCl_3$) spectrum of 3la

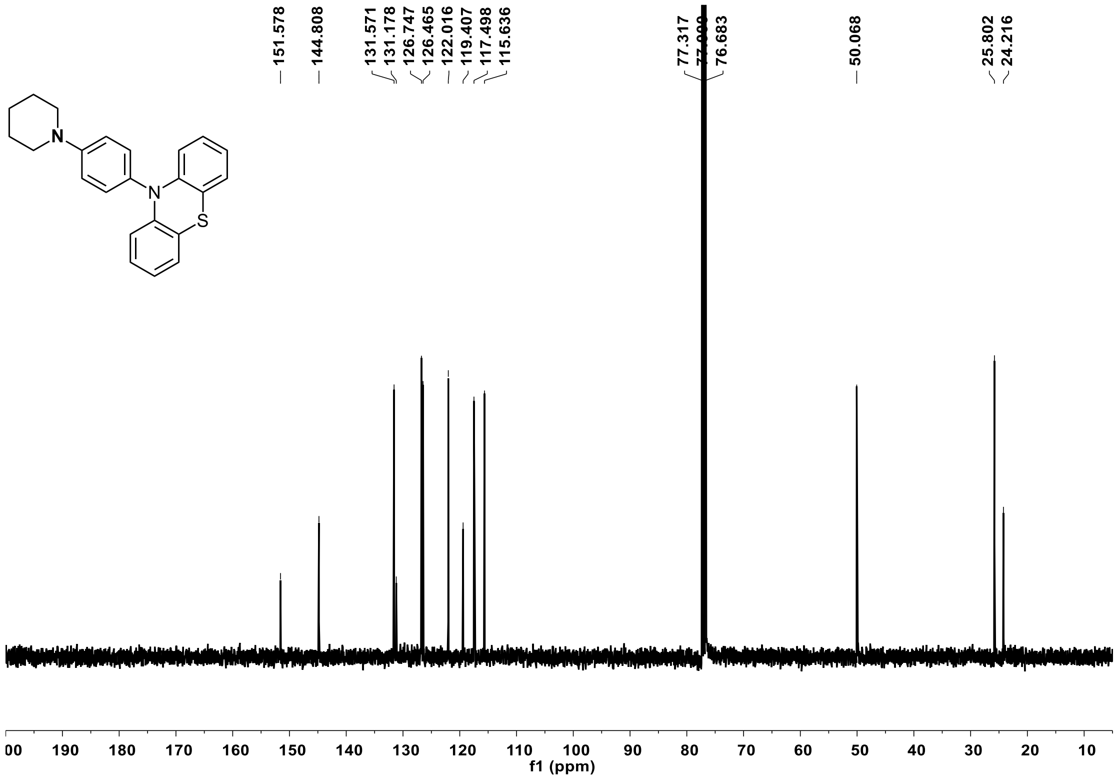

Supplementary Figure 49. ^{13}C NMR (101 MHz, $CDCl_3$) spectrum of 3la

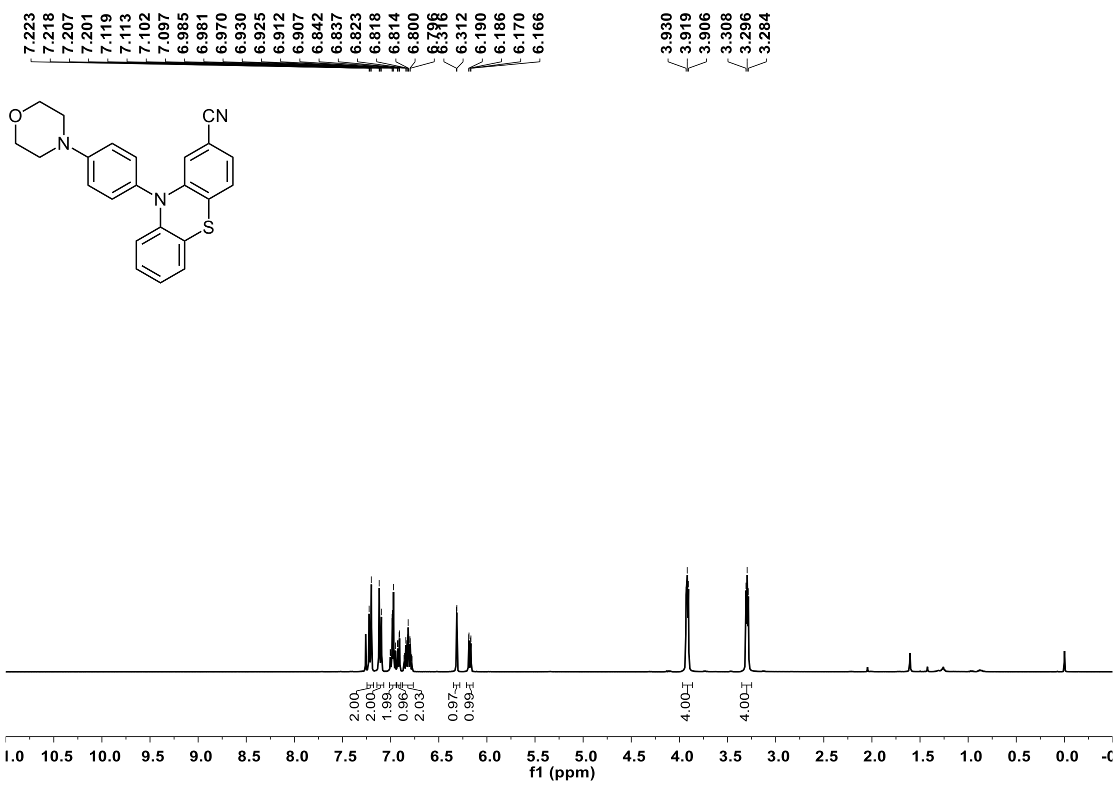

Supplementary Figure 50. ^{1}H NMR (400 MHz, $CDCl_3$) spectrum of 3md

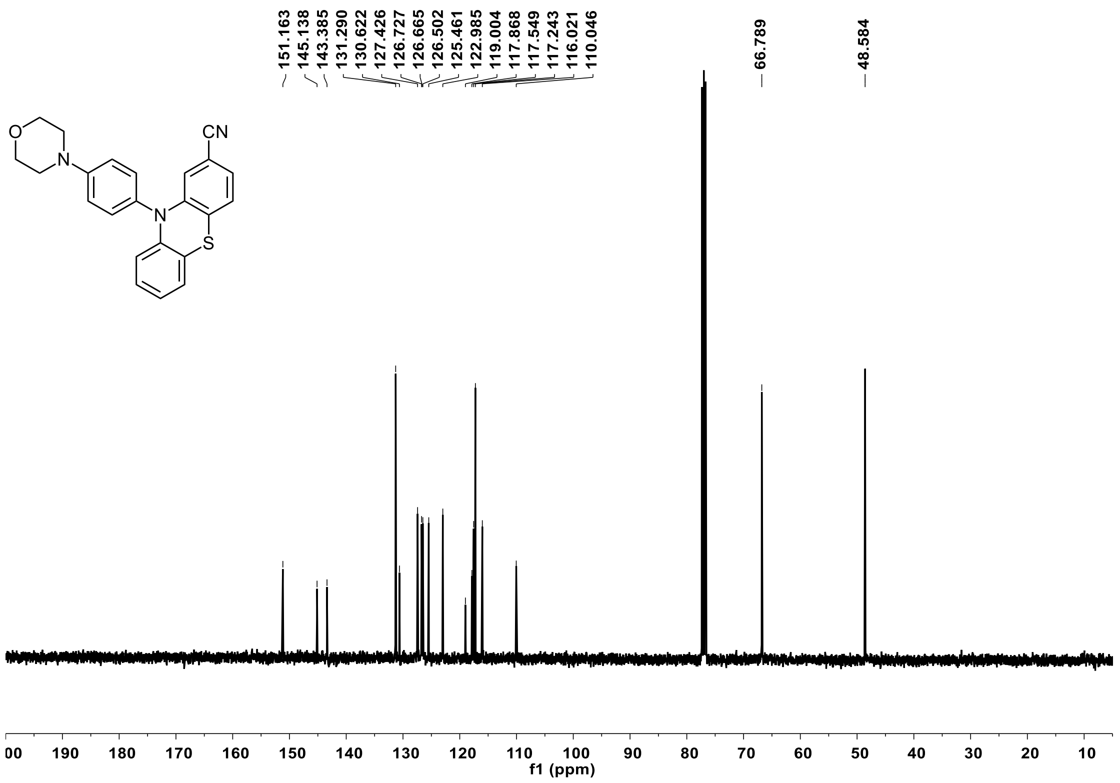

Supplementary Figure 51. ^{13}C NMR (101 MHz, $CDCl_3$) spectrum of 3md

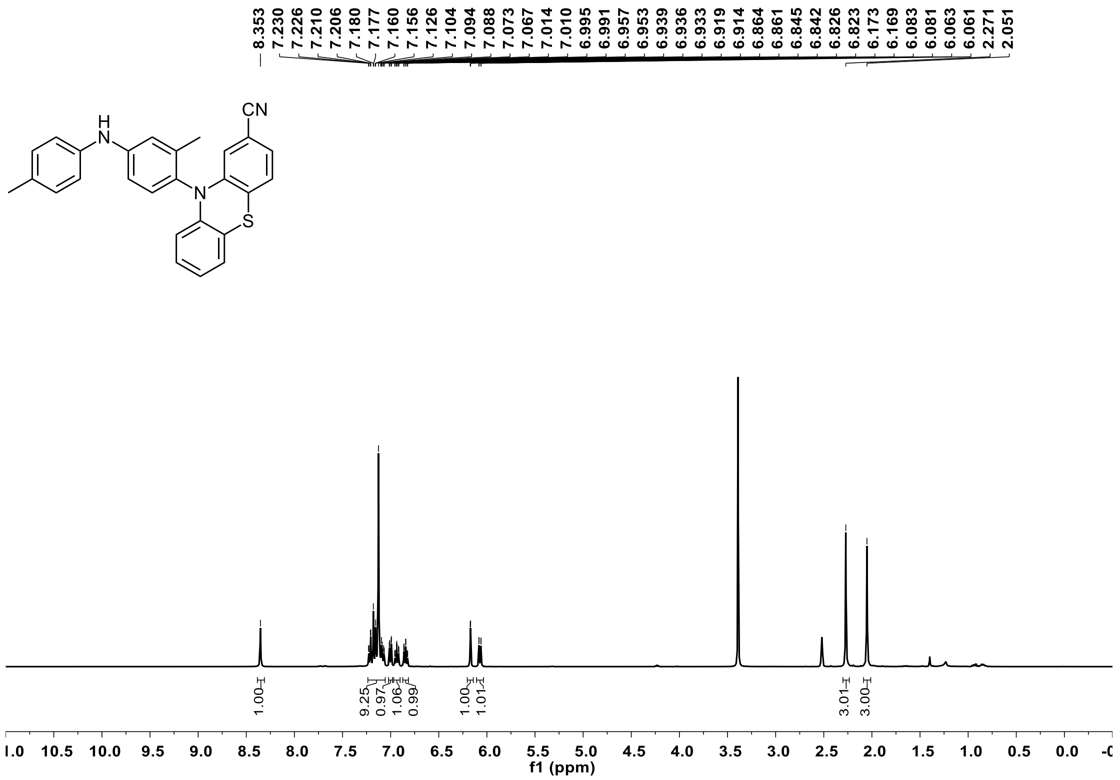

Supplementary Figure 52. ^{1}H NMR (400 MHz, DMSO-d_6) spectrum of 4a

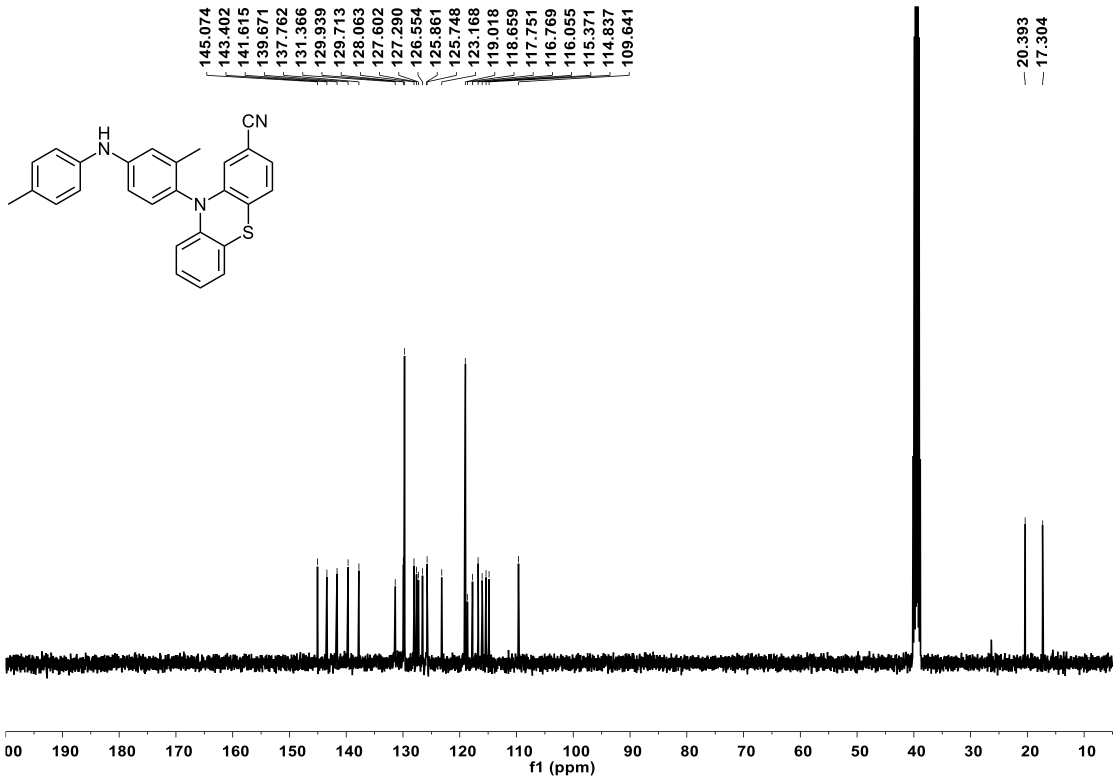

Supplementary Figure 53. ^{13}C NMR (101 MHz, DMSO-d_6) spectrum of 4a

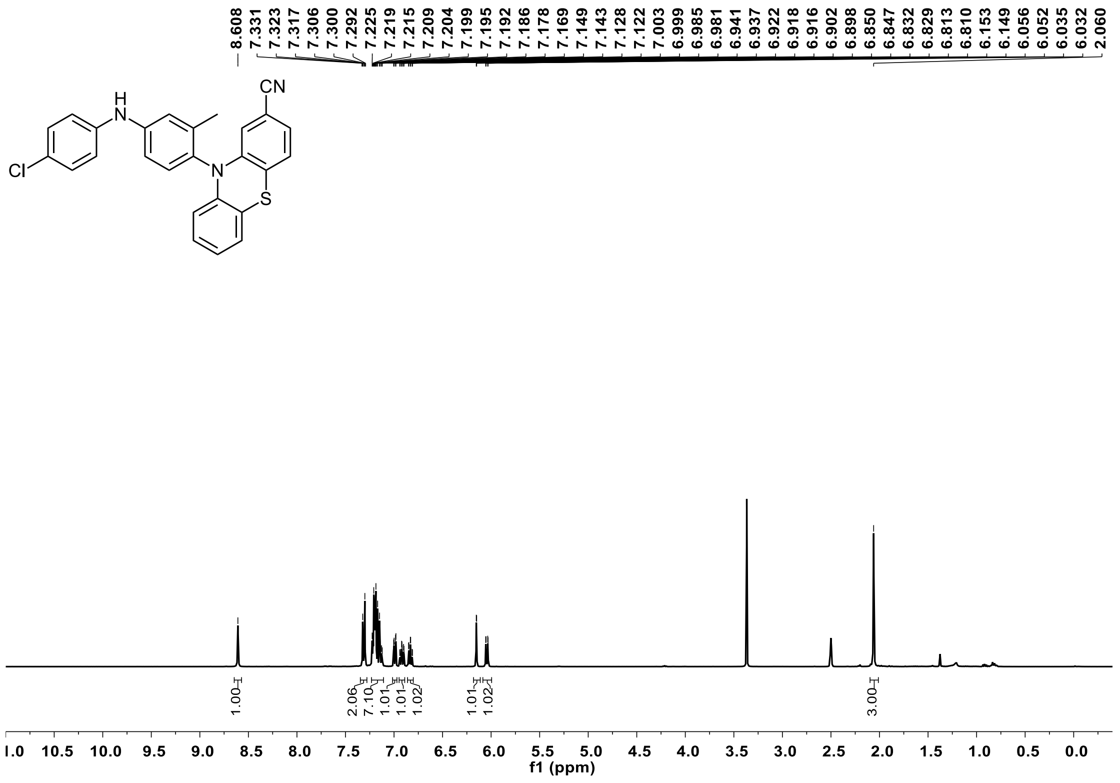

Supplementary Figure 54. ^{1}**H NMR (400 MHz, DMSO-d$_6$) spectrum of 4b**

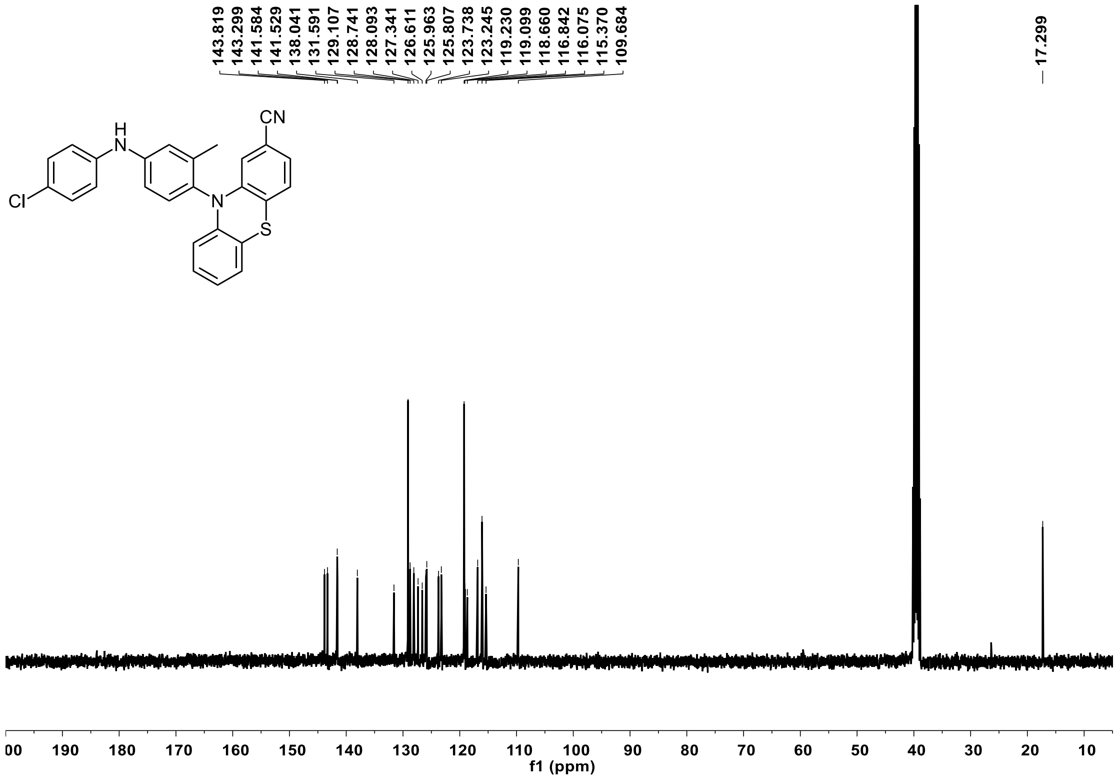

Supplementary Figure 55. ^{13}**C NMR (101 MHz, DMSO-d$_6$) spectrum of 4b**

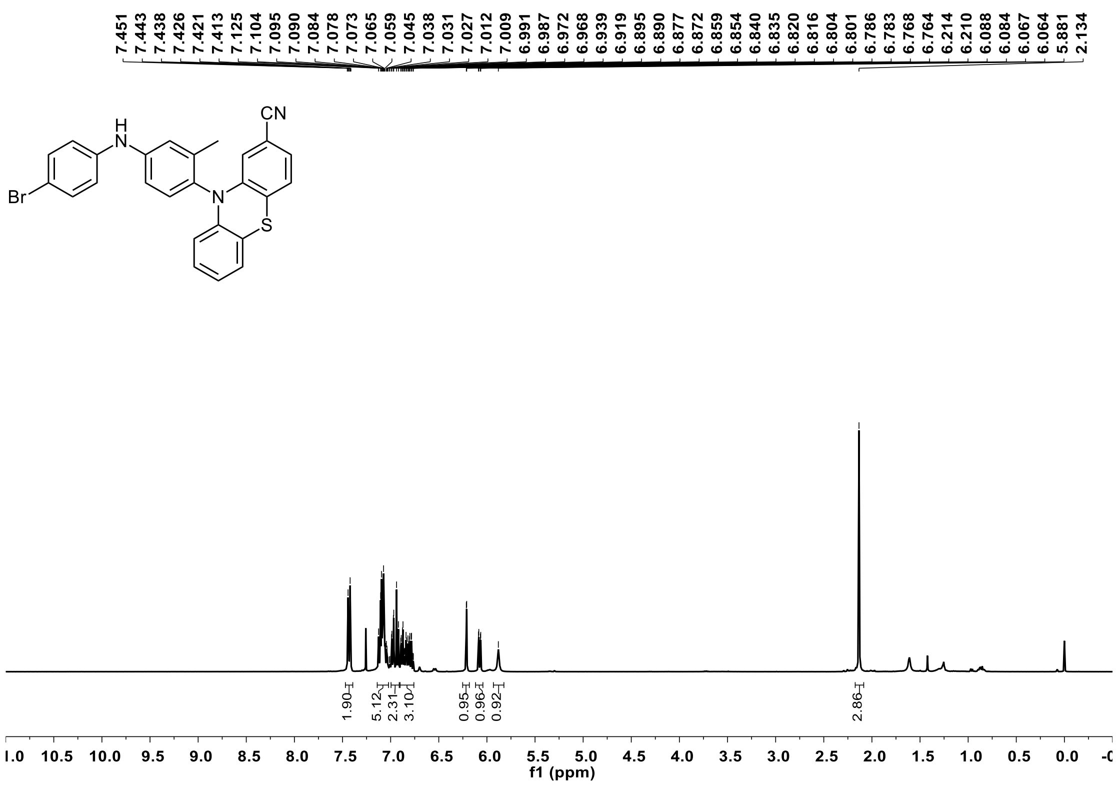

Supplementary Figure 56. ^{1}H NMR (400 MHz, $CDCl_3$) spectrum of 4c

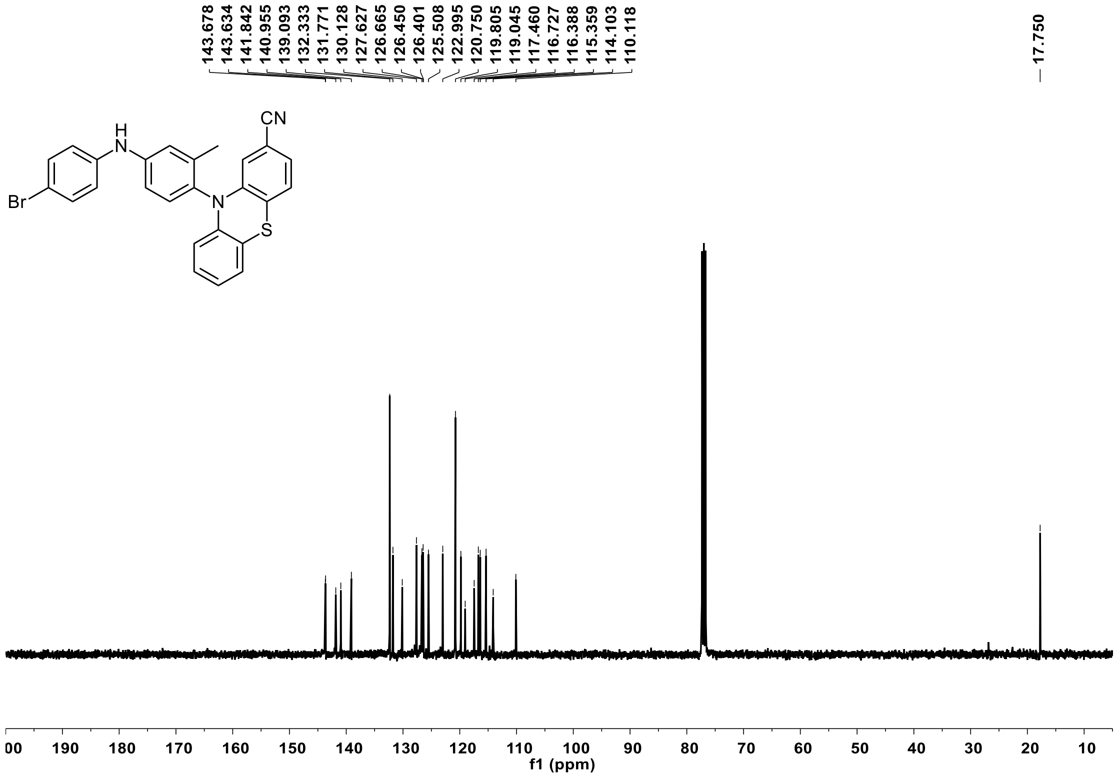

Supplementary Figure 57. ^{13}C NMR (101 MHz, $CDCl_3$) spectrum of 4c

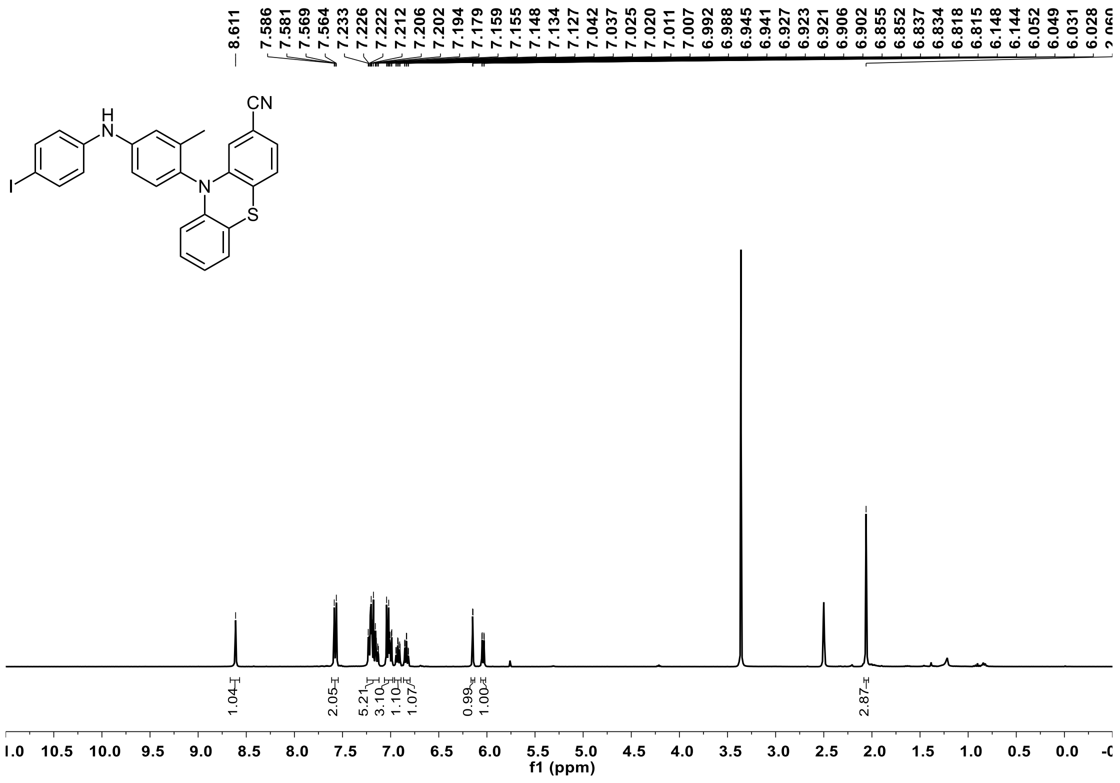

Supplementary Figure 58. ^{1}H NMR (400 MHz, DMSO-d$_6$) spectrum of 4d

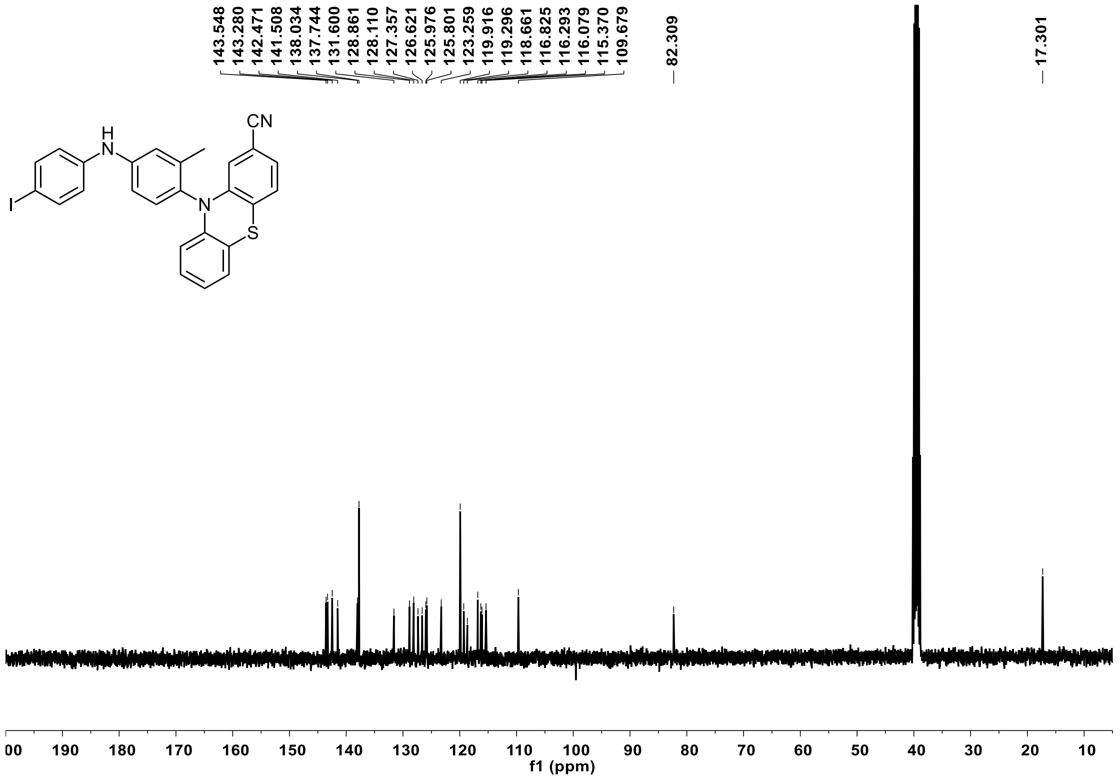

Supplementary Figure 59. ^{13}C NMR (101 MHz, DMSO-d$_6$) spectrum of 4d

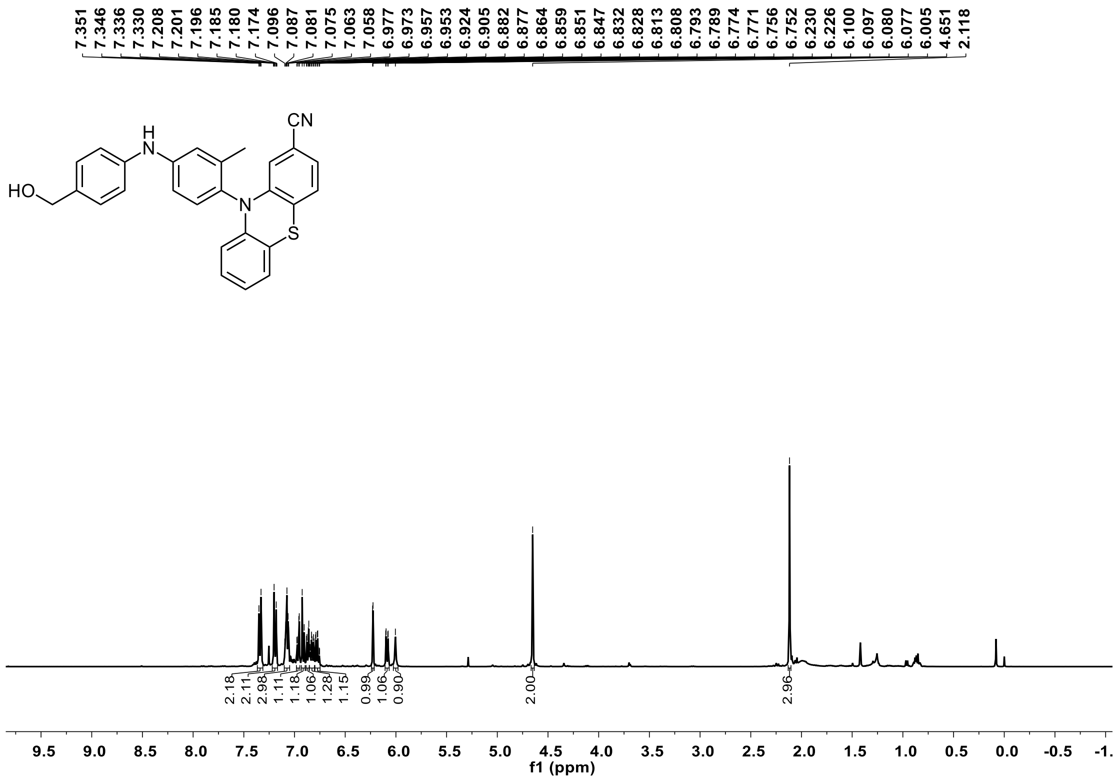

Supplementary Figure 60. ^{1}H NMR (400 MHz, $CDCl_3$) spectrum of 4e

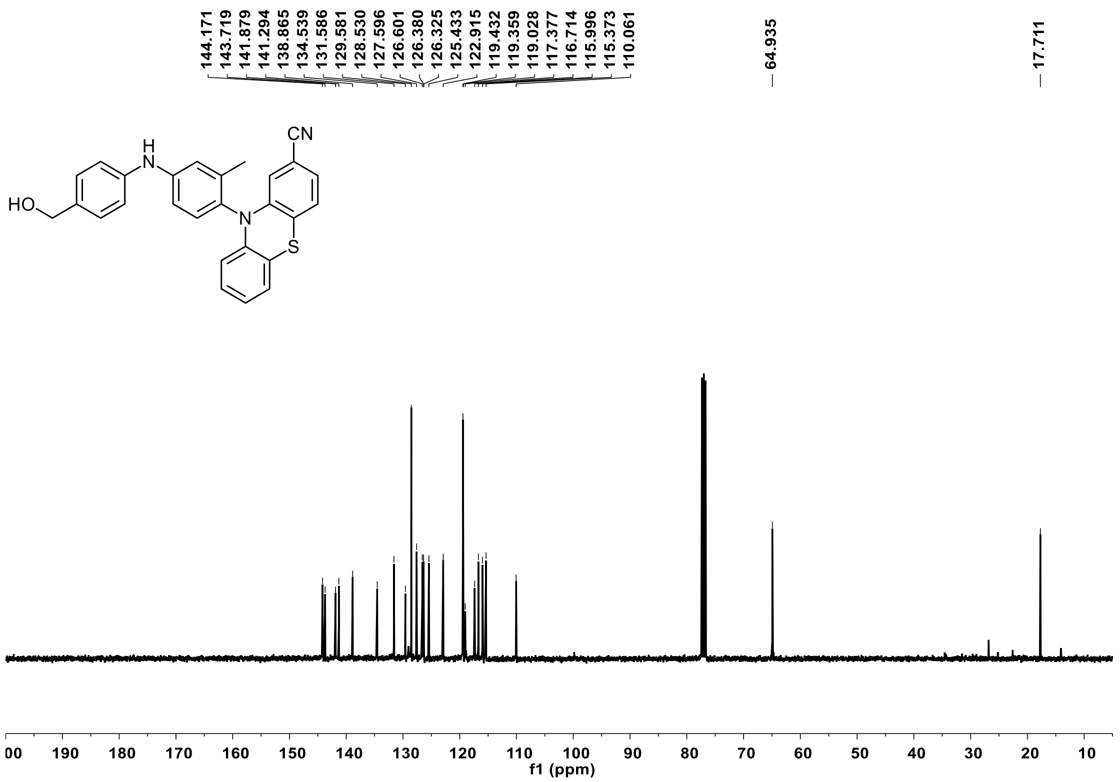

Supplementary Figure 61. ^{13}C NMR (101 MHz, $CDCl_3$) spectrum of 4e

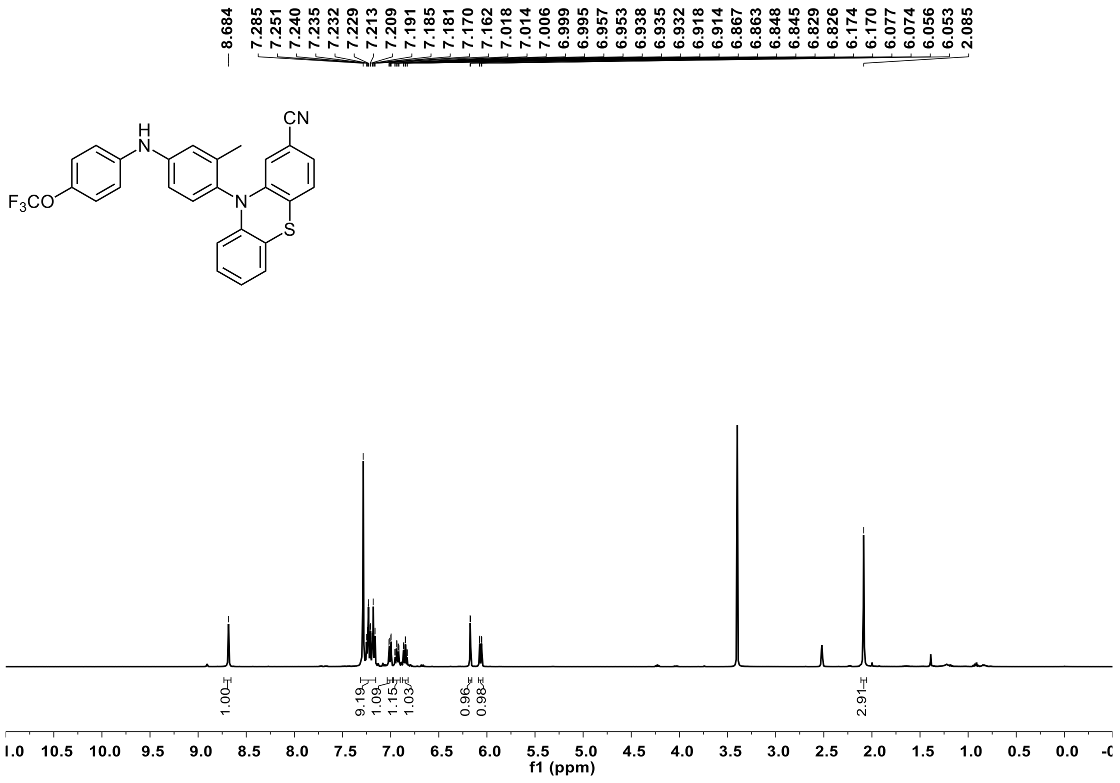

Supplementary Figure 62. ^{1}H NMR (400 MHz, DMSO-d_6) spectrum of 4f

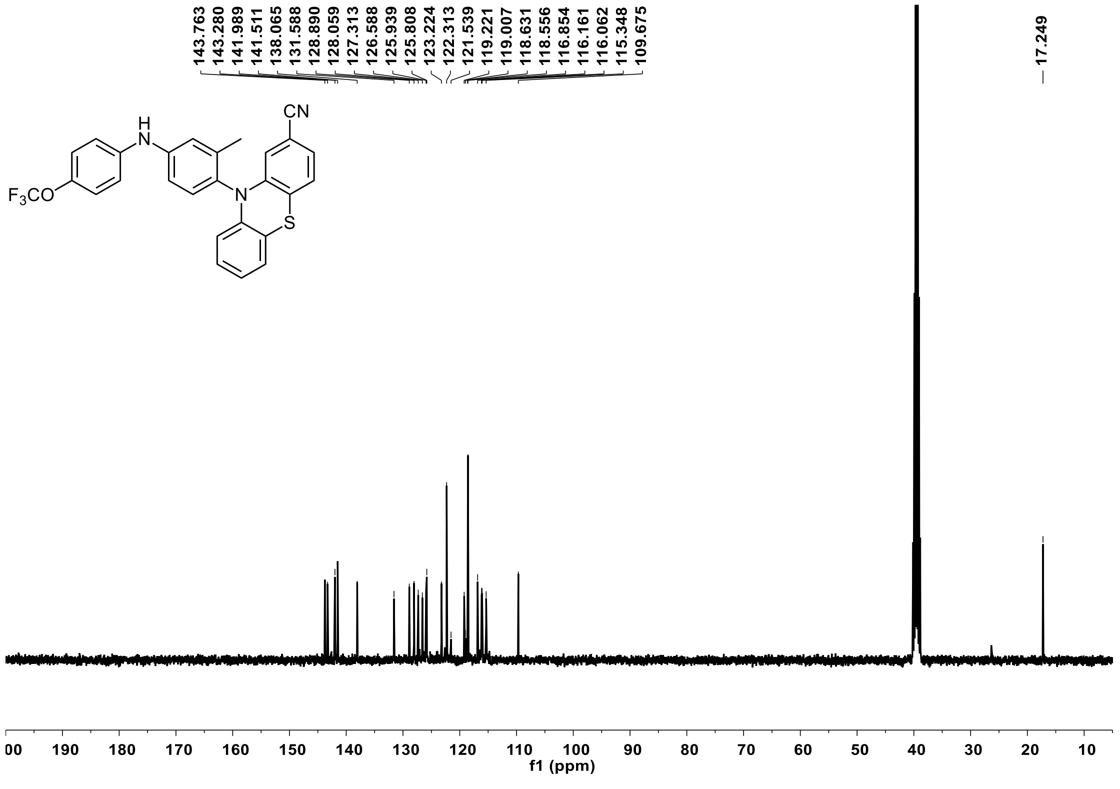

Supplementary Figure 63. ^{13}C NMR (101 MHz, DMSO-d_6) spectrum of 4f

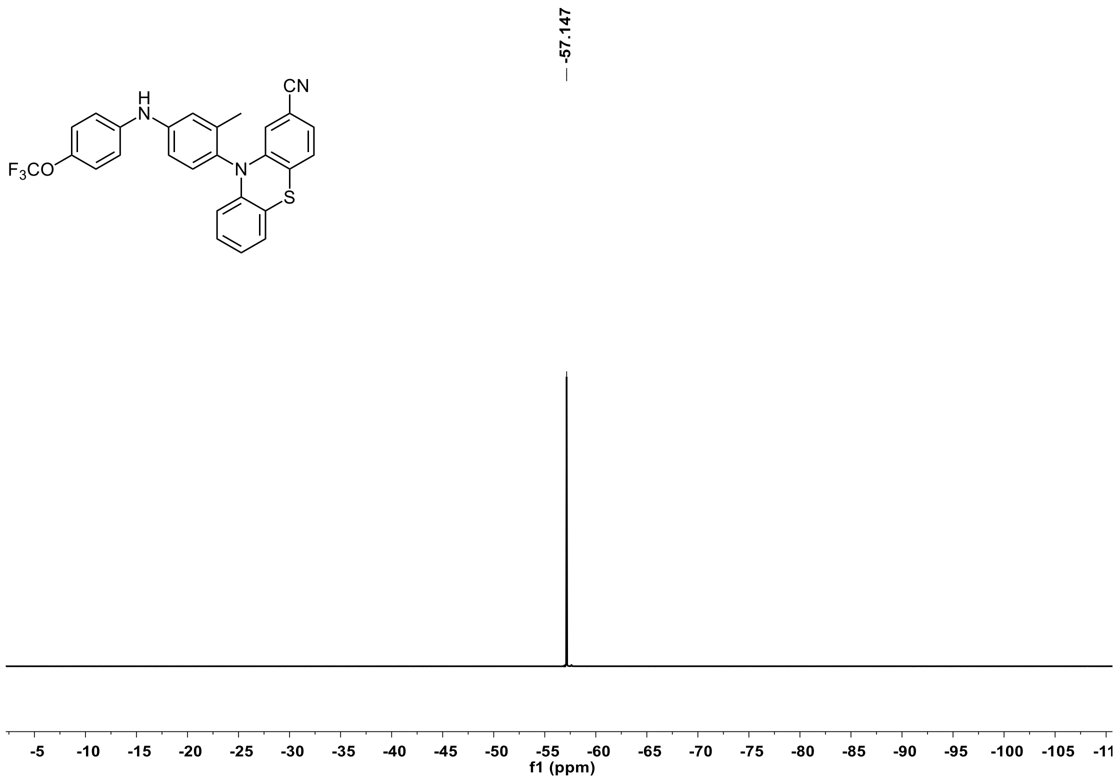

Supplementary Figure 64. ^{19}F NMR (377 MHz, DMSO-d$_6$) spectrum of 4f

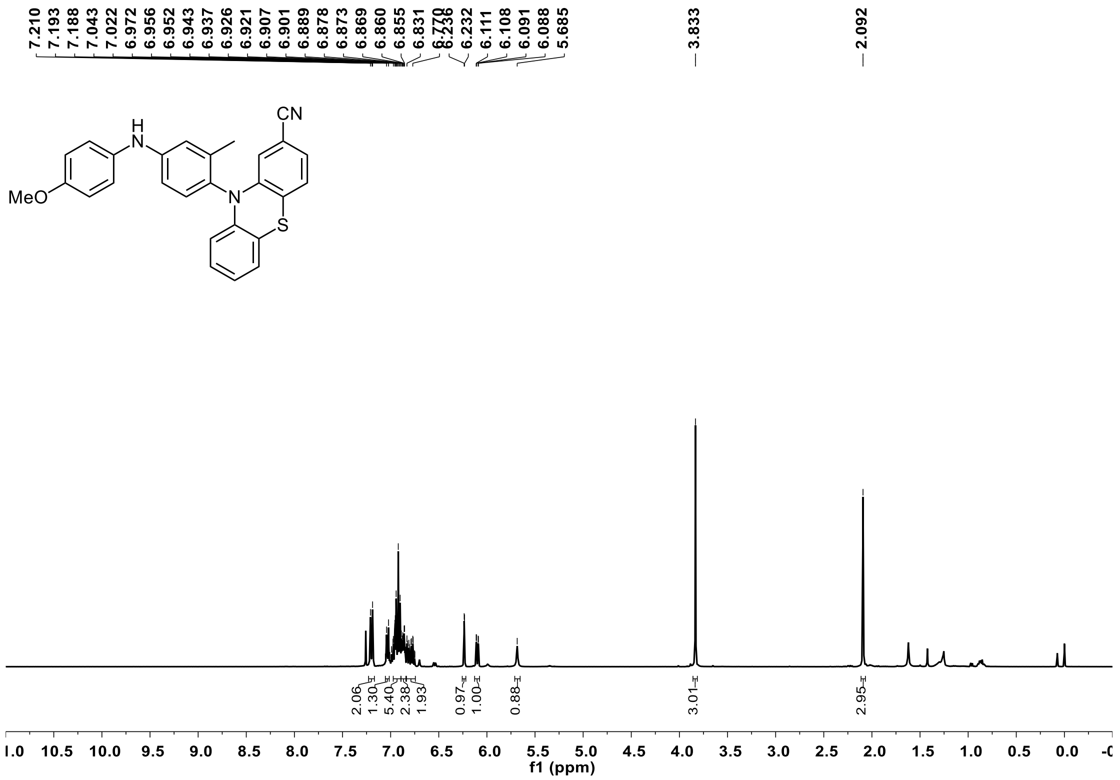

Supplementary Figure 65. ^{1}H NMR (400 MHz, $CDCl_3$) spectrum of 4g

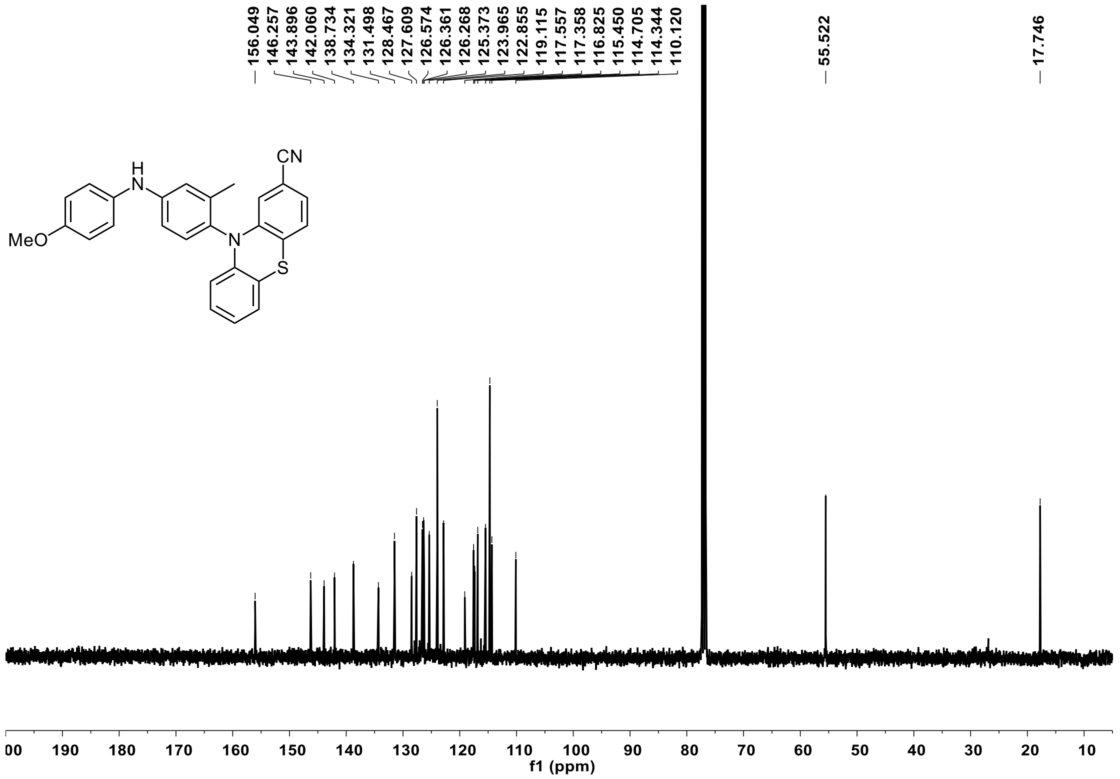

Supplementary Figure 66. ^{13}C NMR (101 MHz, $CDCl_3$) spectrum of 4g

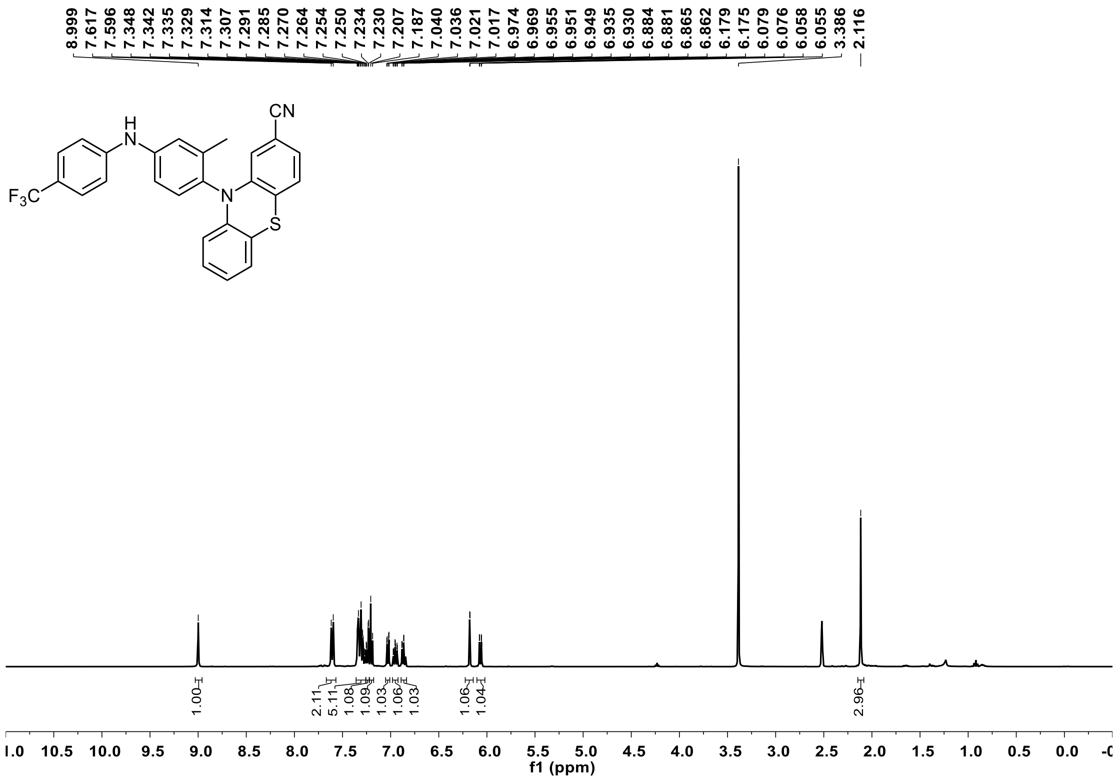

Supplementary Figure 67. ^{1}H NMR (400 MHz, DMSO-d_6) spectrum of 4h

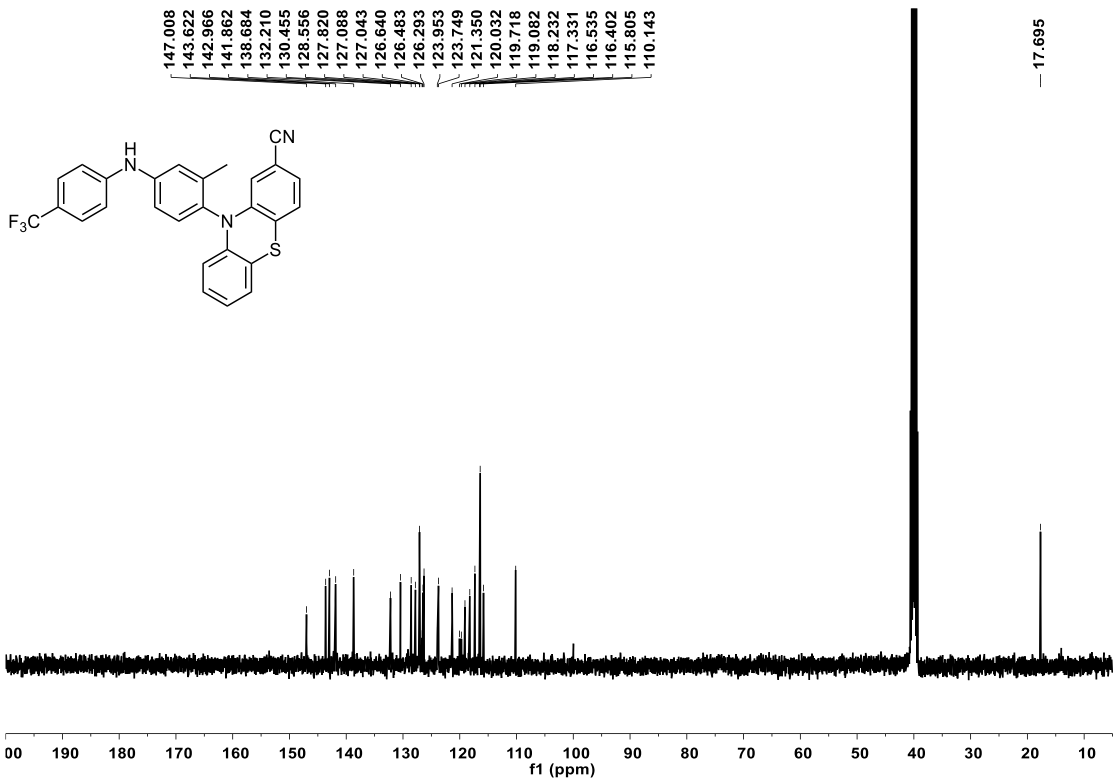

Supplementary Figure 68. ^{13}C NMR (101 MHz, DMSO-d_6) spectrum of 4h

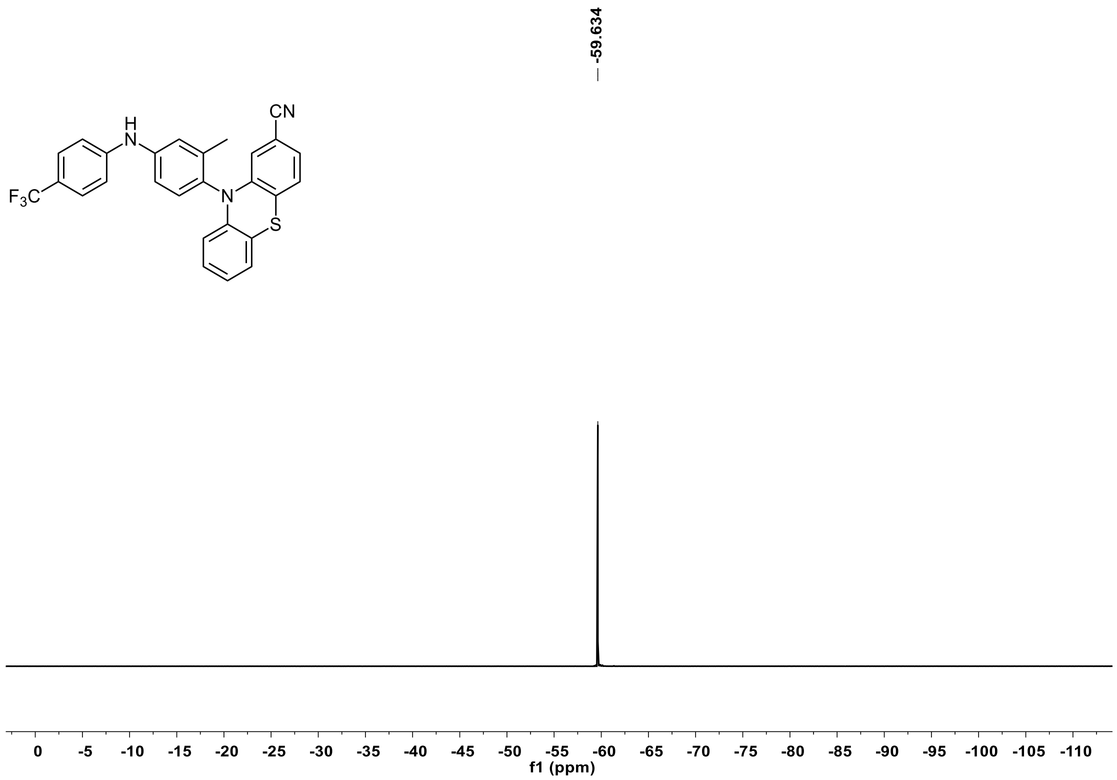

Supplementary Figure 69. ^{19}F NMR (377 MHz, DMSO-d_6) spectrum of 4h

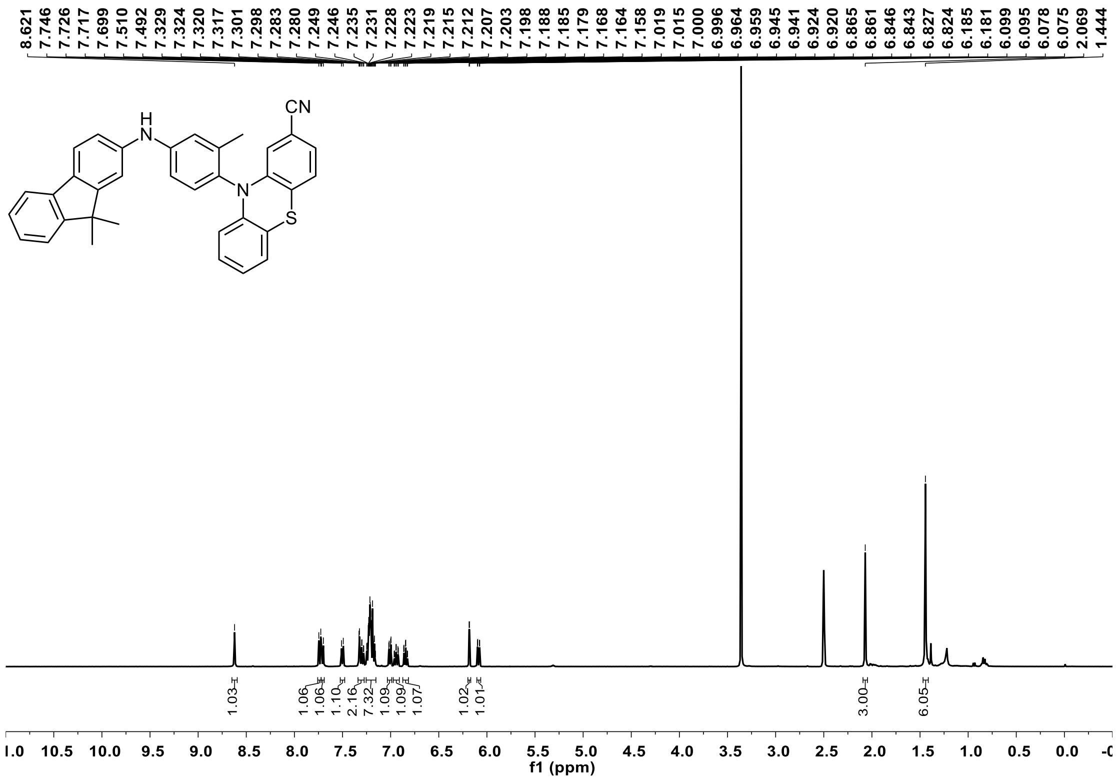

Supplementary Figure 70. ^{1}H **NMR (400 MHz, DMSO-d$_6$) spectrum of 4i**

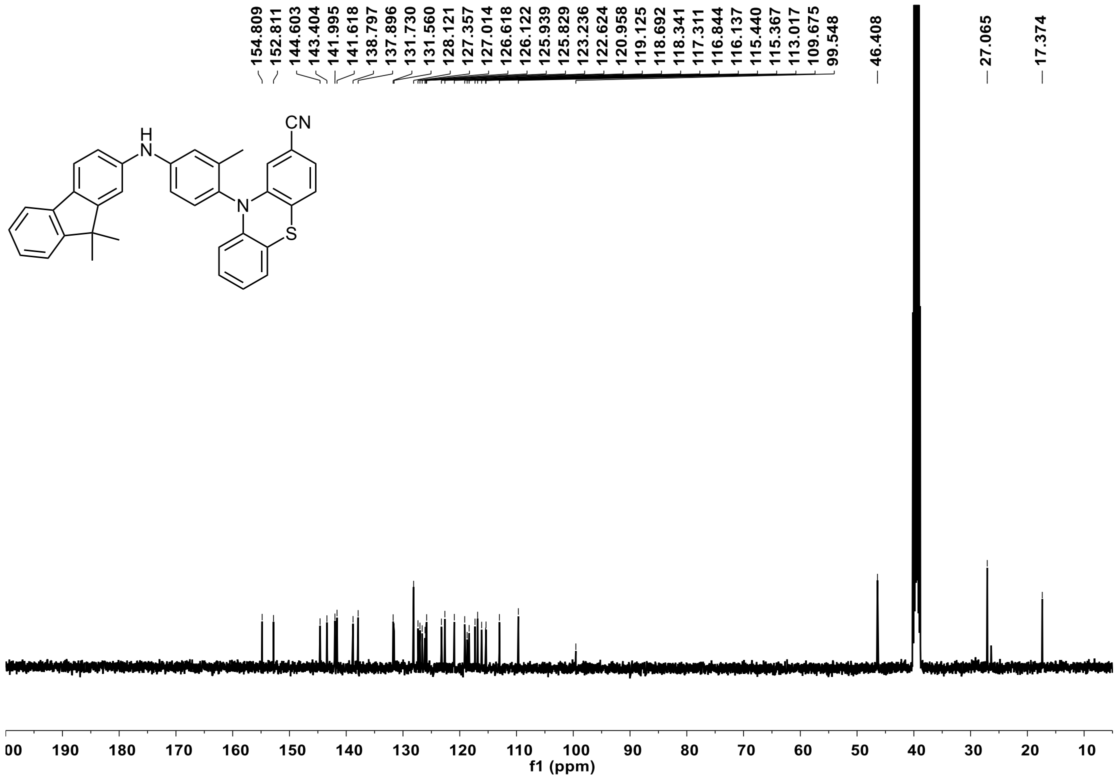

Supplementary Figure 71. ^{13}C **NMR (101 MHz, DMSO-d$_6$) spectrum of 4i**

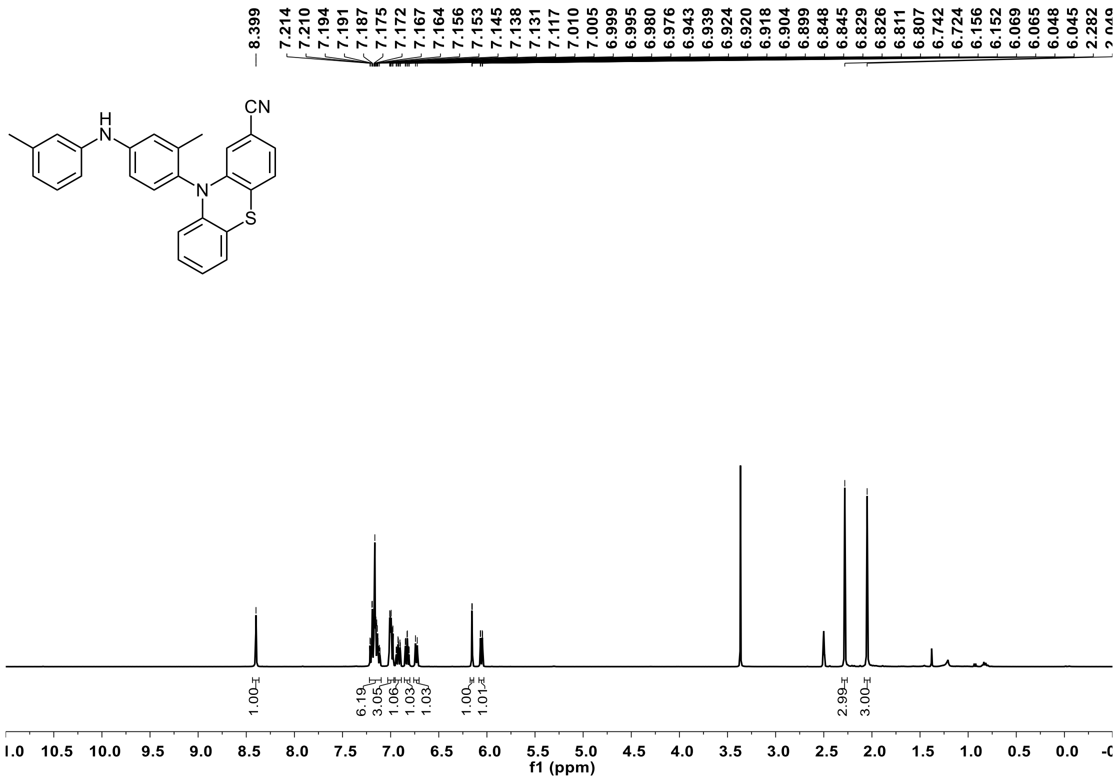

Supplementary Figure 72. ^{1}H NMR (400 MHz, DMSO-d_6) spectrum of 4j

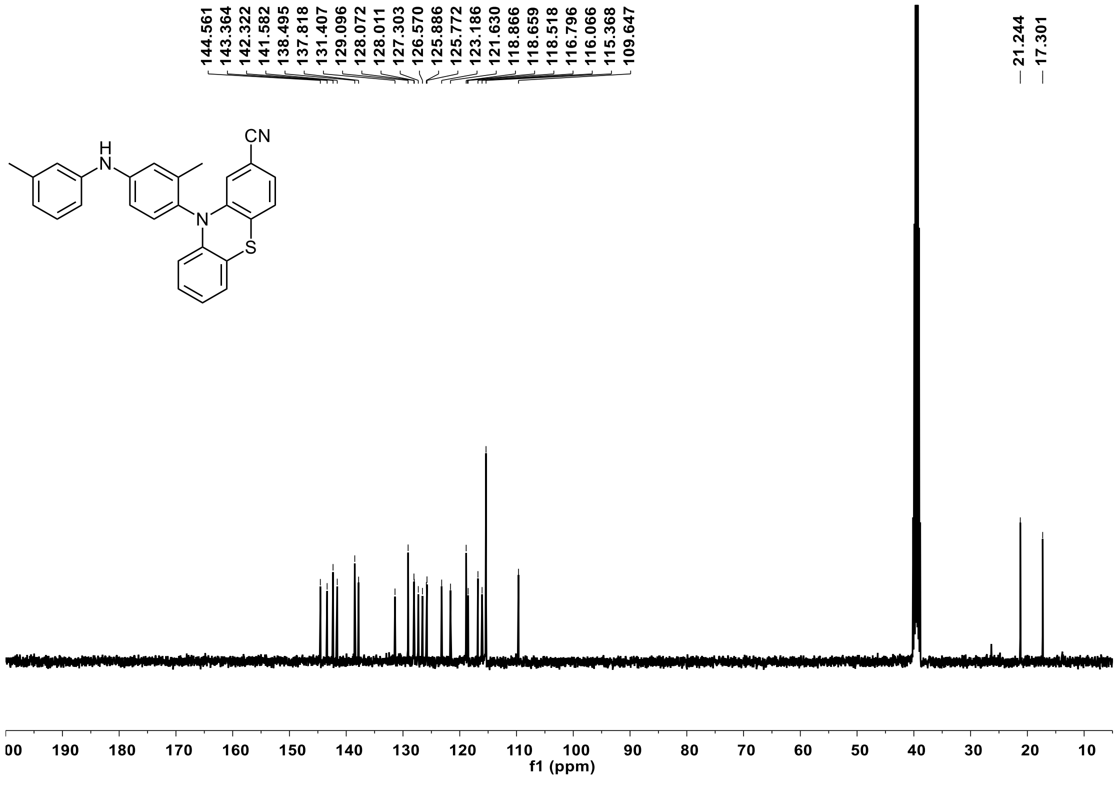

Supplementary Figure 73. ^{13}C NMR (101 MHz, DMSO-d_6) spectrum of 4j

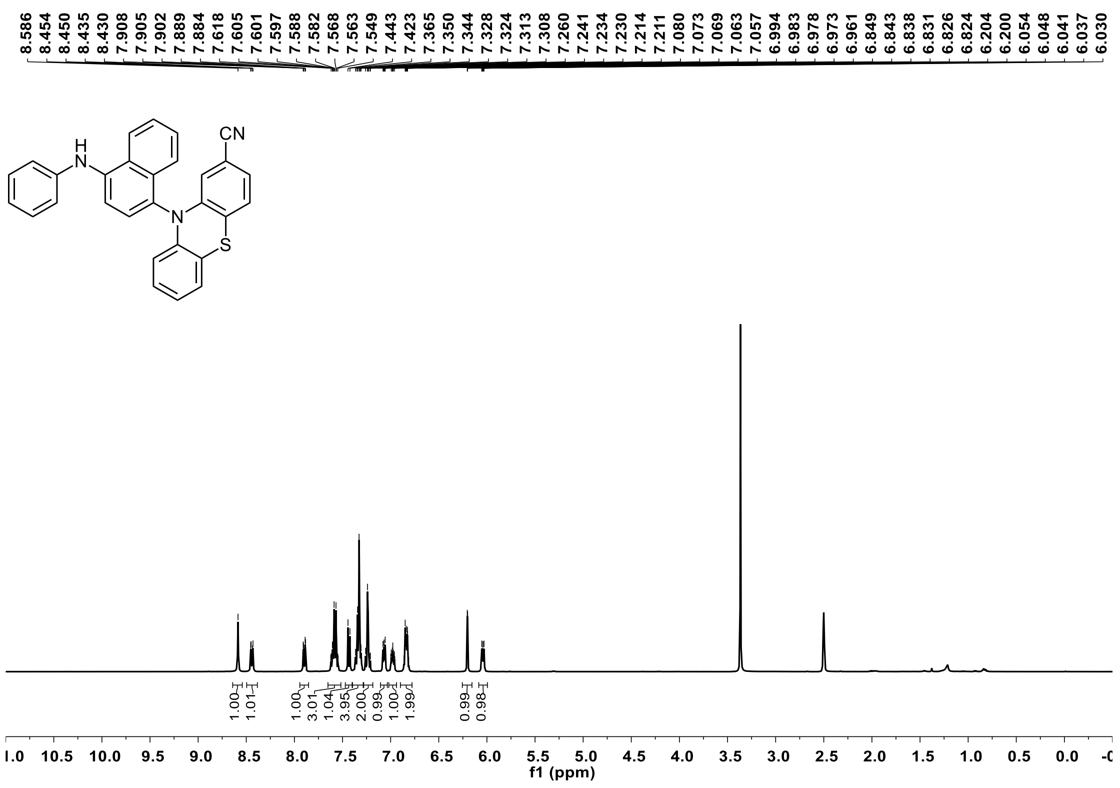

Supplementary Figure 74. ^{1}H NMR (400 MHz, DMSO-d_6) spectrum of 4k

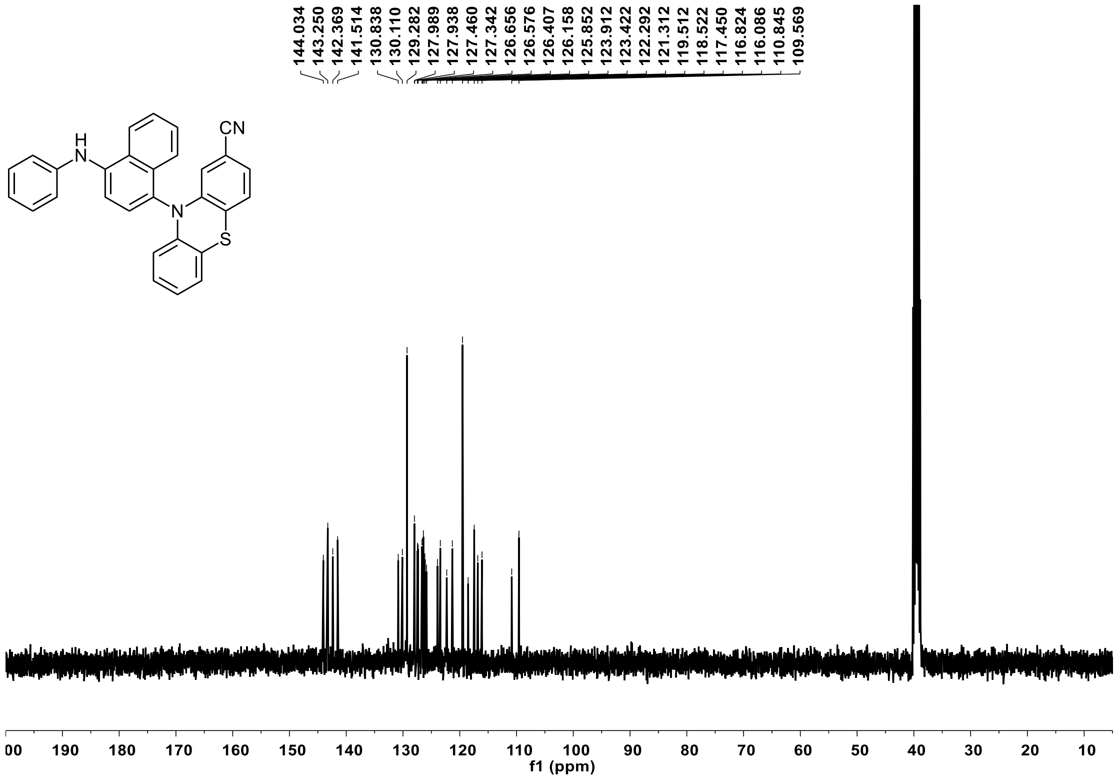

Supplementary Figure 75. ^{13}C NMR (101 MHz, DMSO-d_6) spectrum of 4k

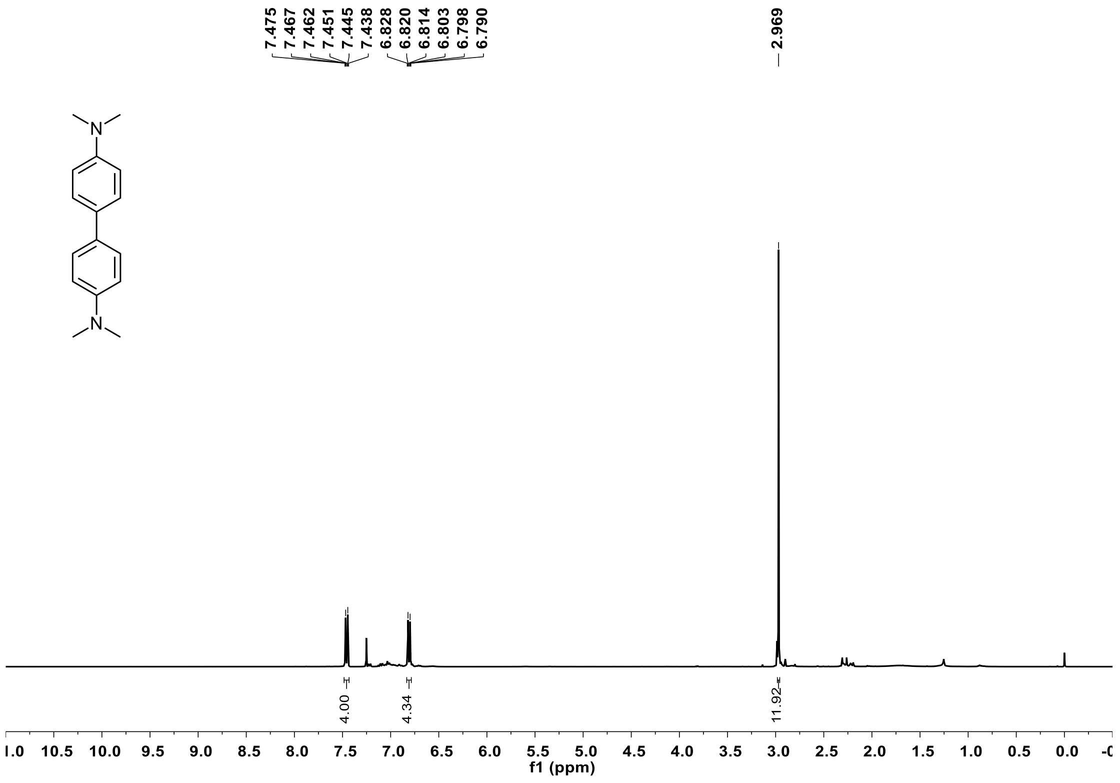

Supplementary Figure 76. ^{1}H NMR (400 MHz, $CDCl_3$) spectrum of 5a

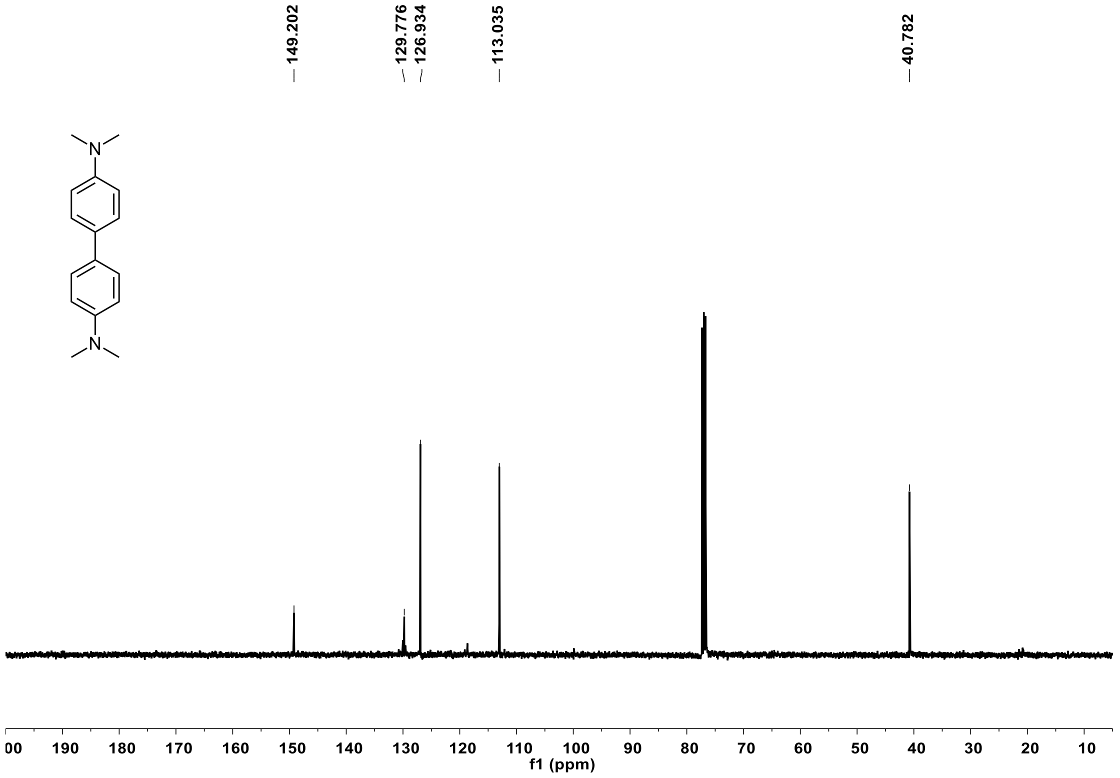

Supplementary Figure 77. ^{13}C NMR (101 MHz, $CDCl_3$) spectrum of 5a

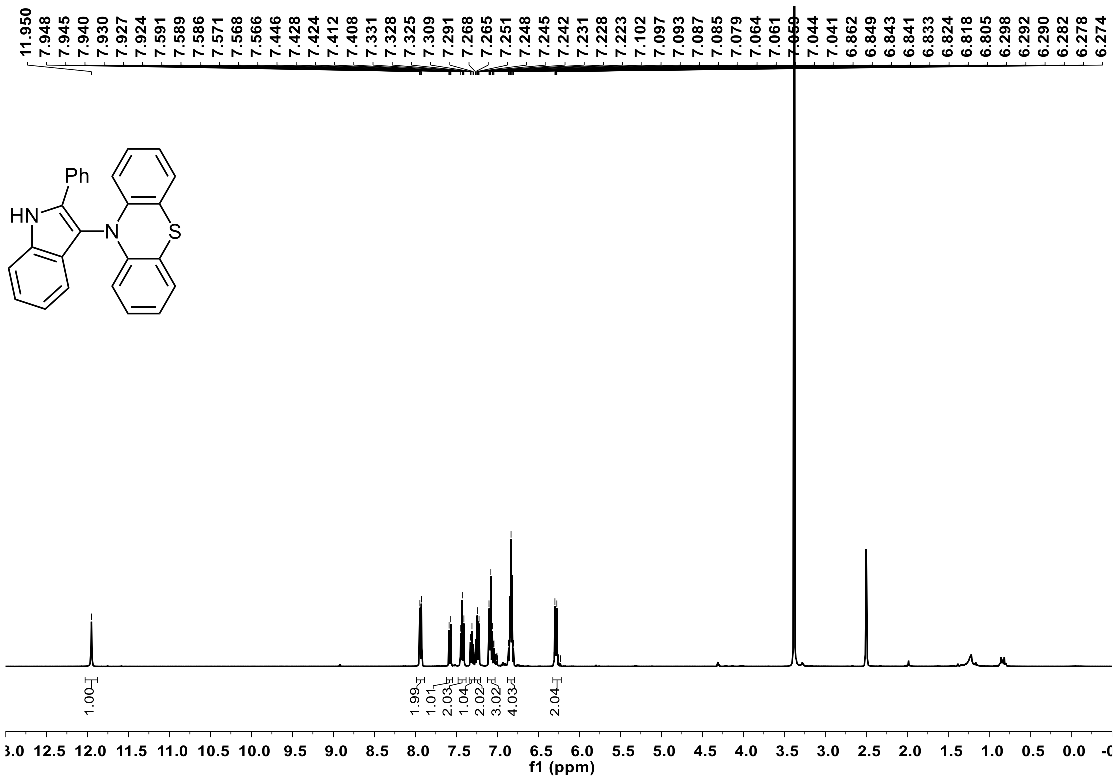

Supplementary Figure 78. ^{1}H NMR (400 MHz, DMSO-d_6) spectrum of 6a

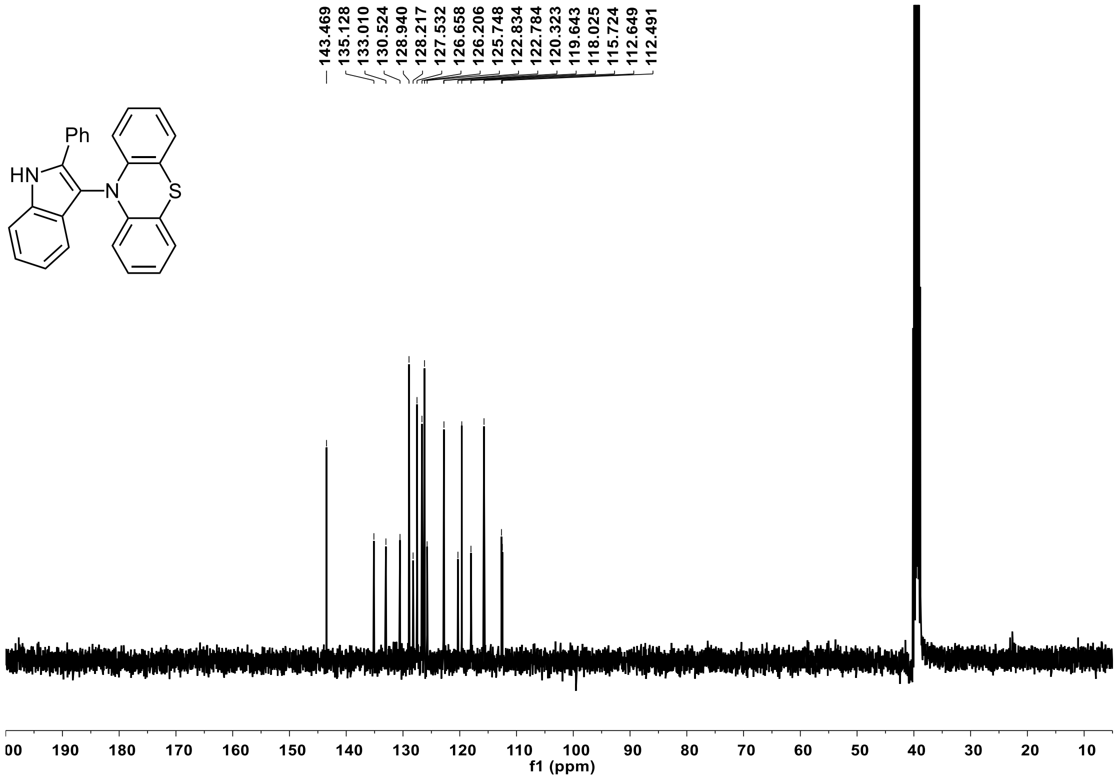

Supplementary Figure 79. ^{13}C NMR (101 MHz, DMSO-d_6) spectrum of 6a

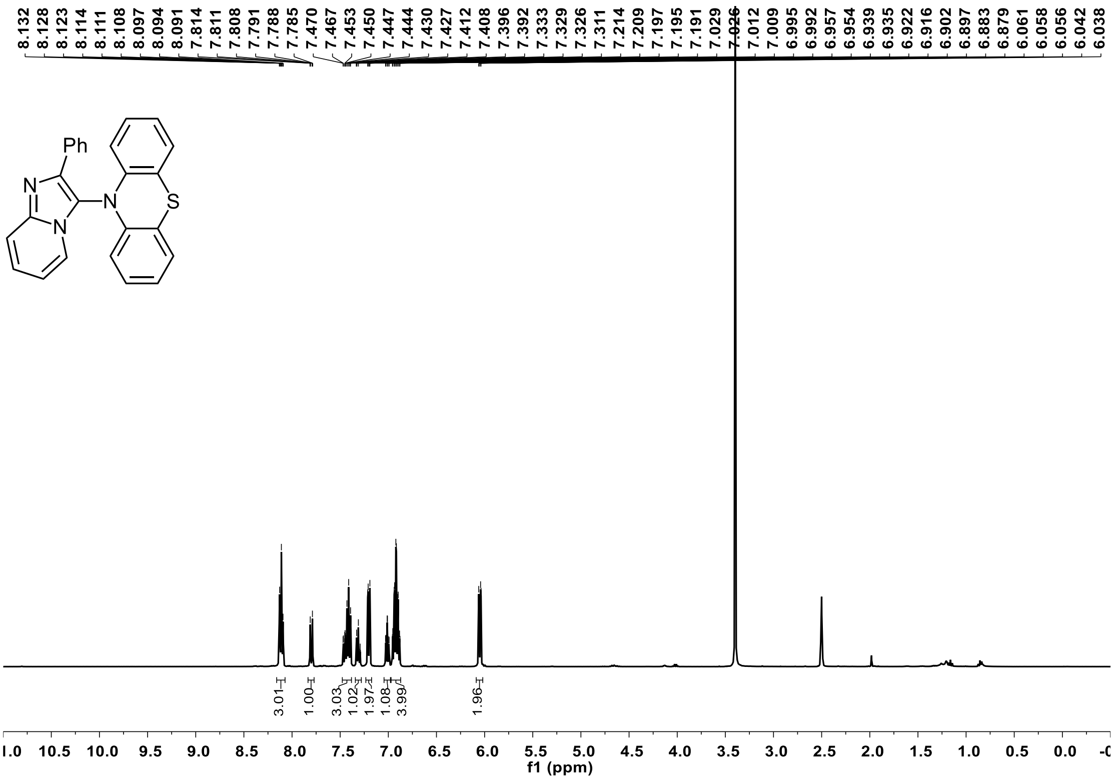

Supplementary Figure 80. **^{1}H NMR (400 MHz, DMSO-d_6) spectrum of 6b**

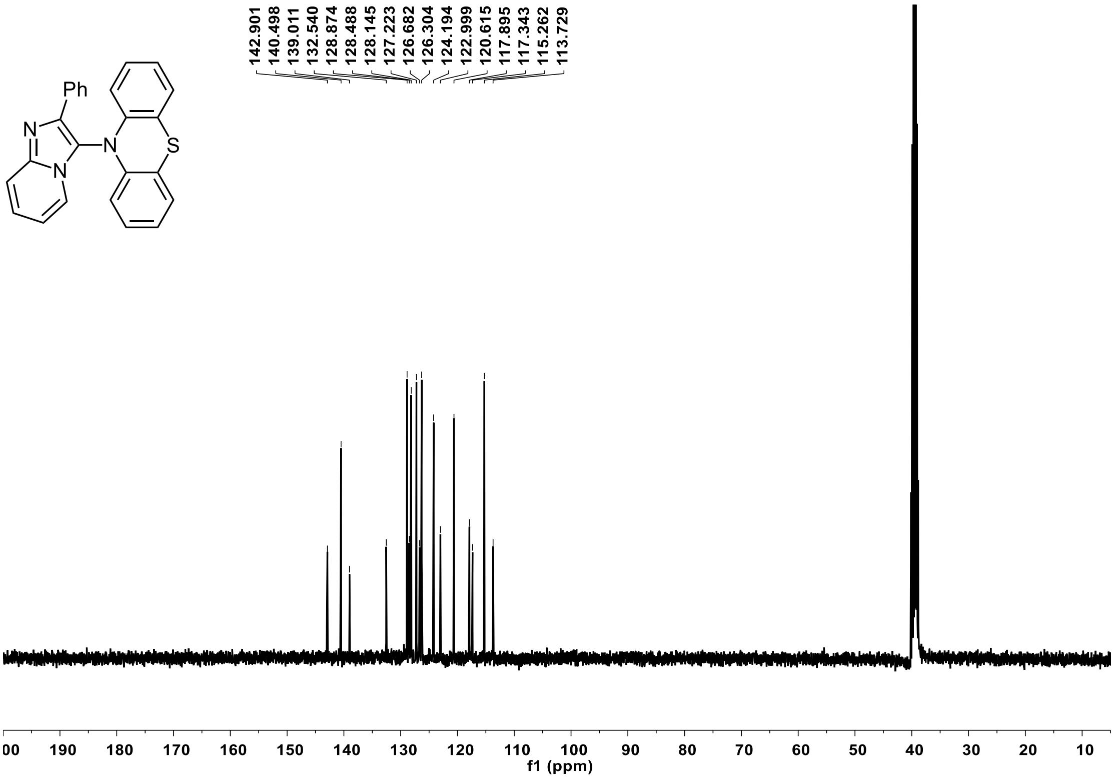

Supplementary Figure 81. **^{13}C NMR (101 MHz, DMSO-d_6) spectrum of 6b**

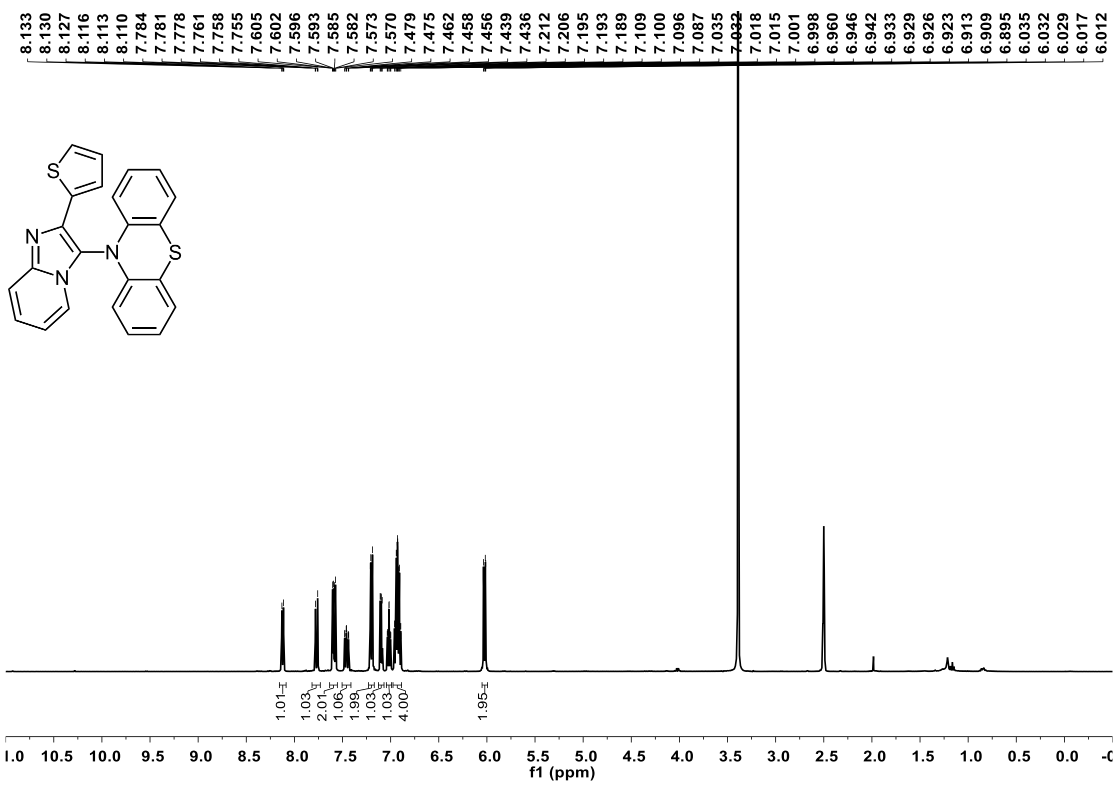

Supplementary Figure 82. ^{1}H NMR (400 MHz, DMSO-d_6) spectrum of 6c

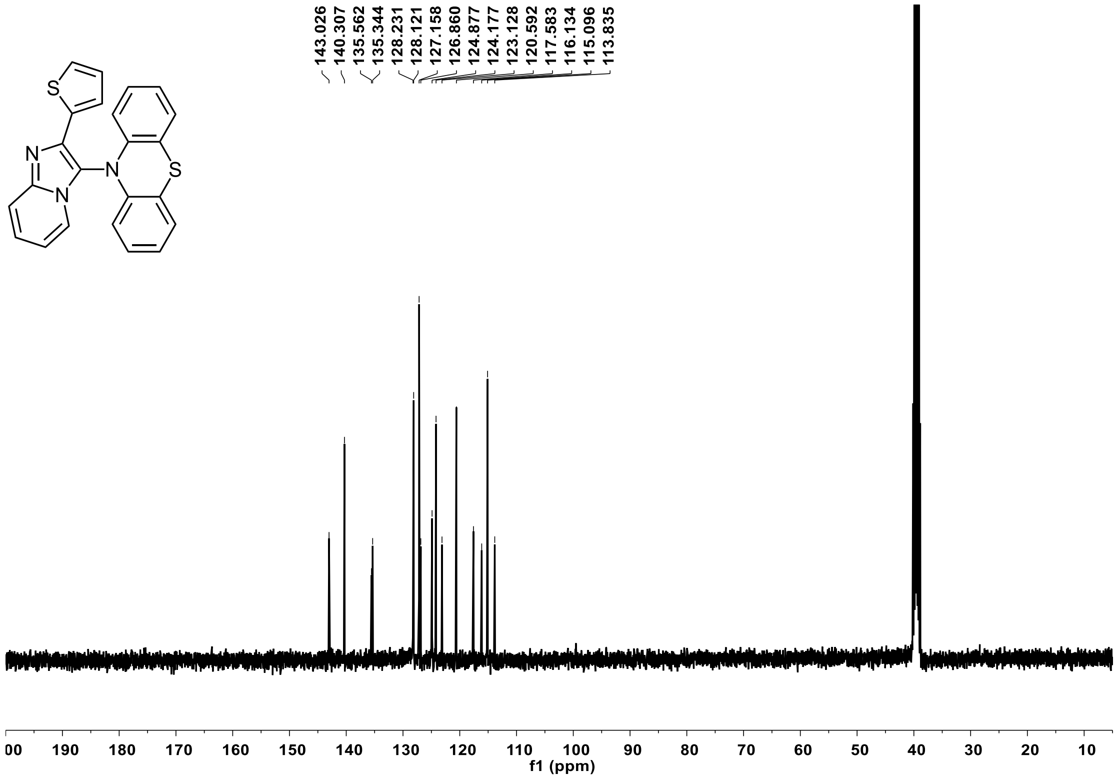

Supplementary Figure 83. ^{13}C NMR (101 MHz, DMSO-d_6) spectrum of 6c

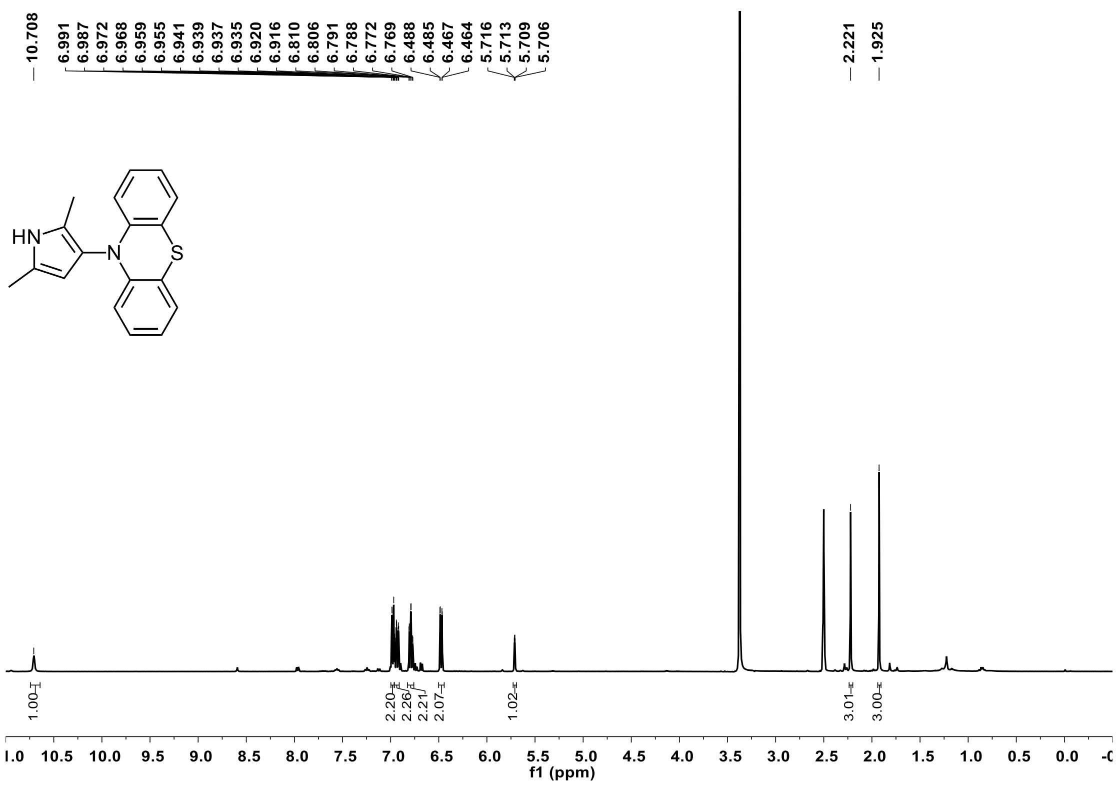

Supplementary Figure 84. ^{1}H NMR (400 MHz, DMSO-d_6) spectrum of 6d

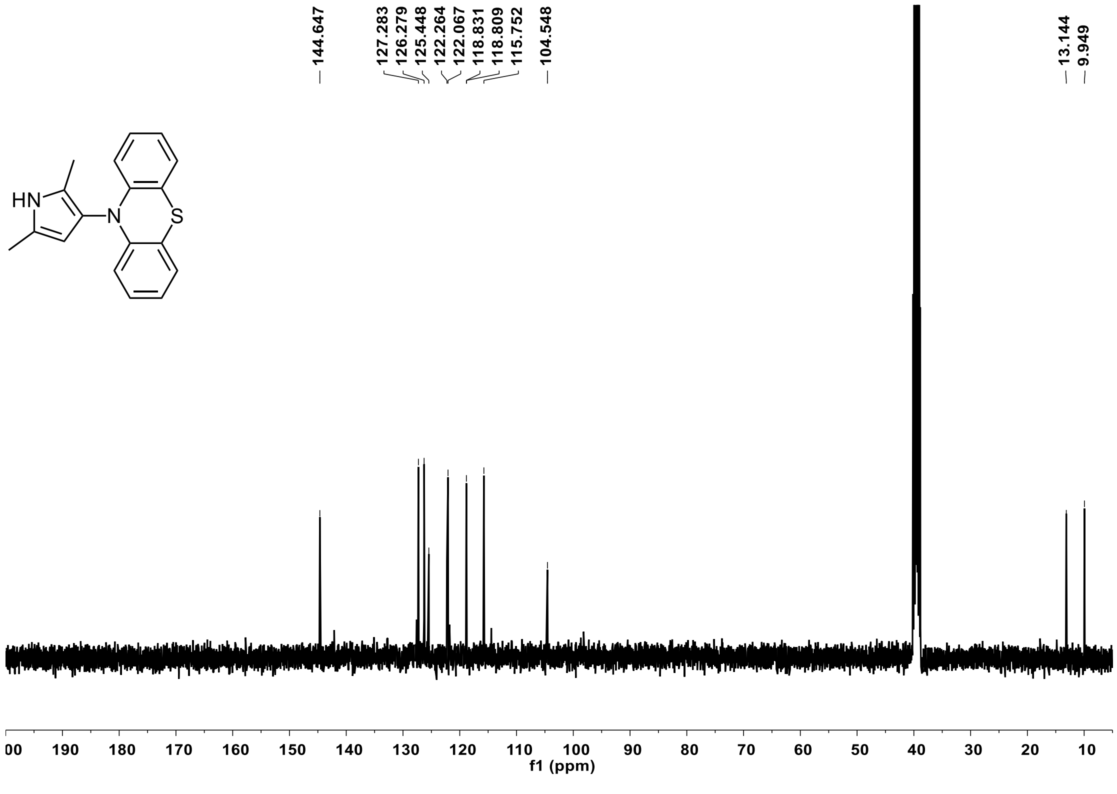

Supplementary Figure 85. ^{13}C NMR (101 MHz, DMSO-d_6) spectrum of 6d

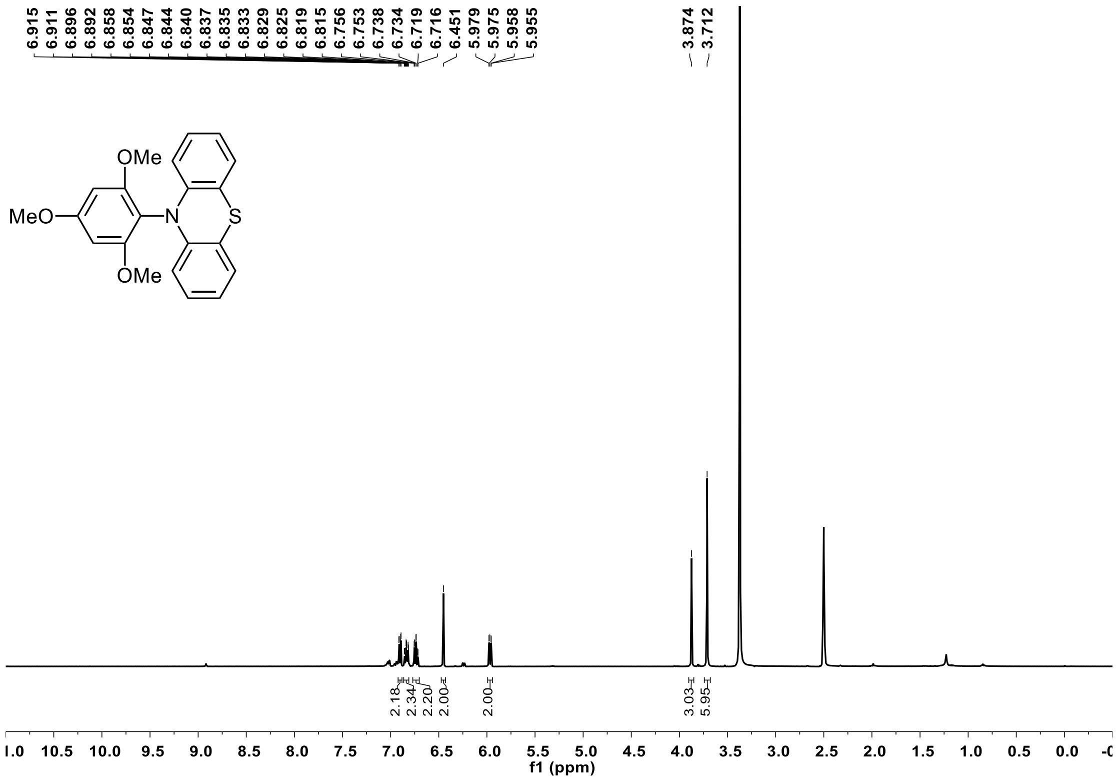

Supplementary Figure 86. ^{1}H NMR (400 MHz, DMSO-d_6) spectrum of 6e

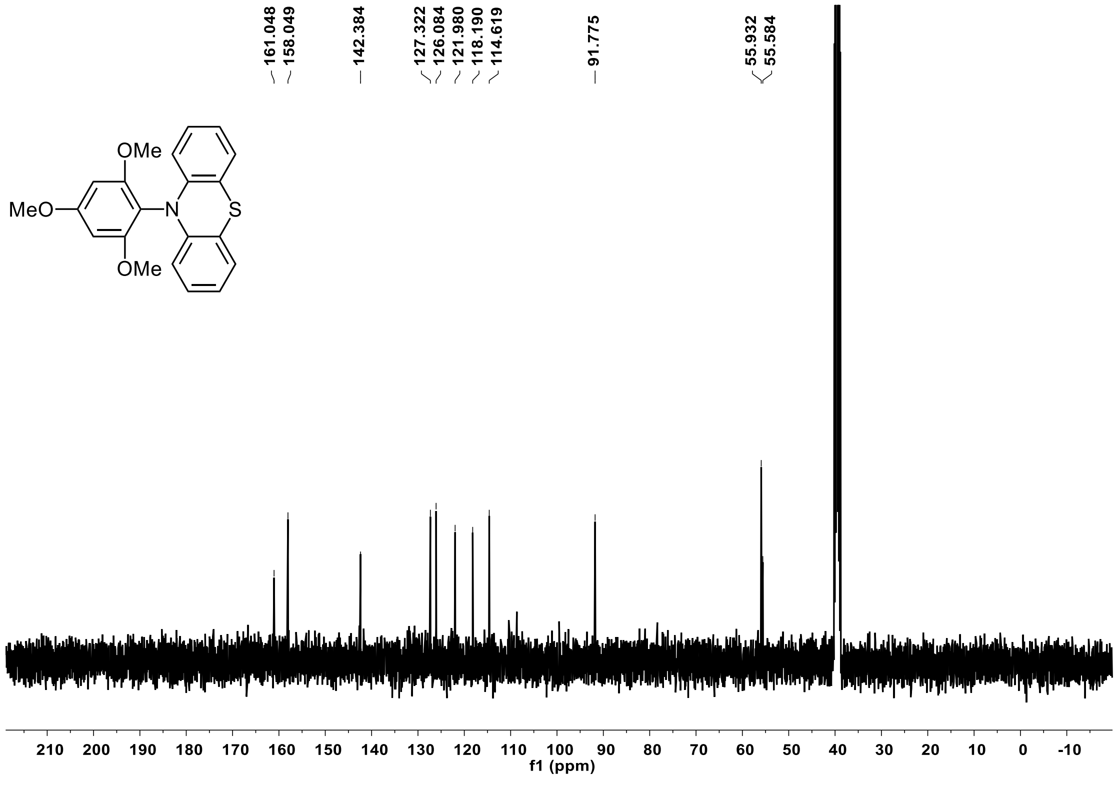

Supplementary Figure 87. ^{13}C NMR (101 MHz, DMSO-d_6) spectrum of 6e

Supplementary References

1. Kinashi K., Lee K.-P., Matsumoto S., Ishida K., Ueda. Y. Alkyl substituent effects on J- or H-aggregate formation of bisazomethine dyes. *Dyes and Pigments* **92**, 783-788 (2012).
2. Cano R., Ramon D. J., Yus M. Transition-Metal-Free O-, S-, and N-Arylation of Alcohols, Thiols, Amides, Amines, and Related Heterocycles. *J. Org. Chem.* **76**, 654-660 (2011).
3. Tzschucke C., Murphy J., Hartwig J. F. Arenes to Anilines and Aryl Ethers by Sequential Iridium-Catalyzed Borylation and Copper-Catalyzed Coupling. *Org. Lett.* **9**, 761−764 (2007).
4. Louie J., Driver M. S., Hamann B. C. and Hartwig J. F. Palladium-Catalyzed Amination of Aryl Triflates and Importance of Triflate Addition Rate. *J. Org. Chem.* **62**, 1268-1273 (1997).
5. Zhang H., Cai Q., Ma D. Amino Acid Promoted CuI-Catalyzed C−N Bond Formation between Aryl Halides and Amines or N-Containing Heterocycles. *J. Org. Chem.* **70**, 5164−5173 (2005).
6. Toru A., Riyo S., Toshikazu H. Quinonediimine-Induced Oxidative Coupling of Organomagnesium Reagents. *Chem. Eur. J.* **20**, 653-656 (2014).
7. Zhao Y., Huang B., Yang C., Xia W. Visible-Light-Promoted Direct Amination of Phenols via Oxidative Cross-Dehydrogenative Coupling Reaction. *Org. Lett.* **18**, 3326-3329 (2016).
